# α‑C(sp^3^)–H Acetalization of Cyclic and Aliphatic Ethers Mediated by *N*‑Bromoamides

**DOI:** 10.1021/acs.orglett.6c02125

**Published:** 2026-06-24

**Authors:** Qingyu Zhang, Yat-Long Cheng, Ying-Yeung Yeung

**Affiliations:** Department of Chemistry and State Key Laboratory of Synthetic Chemistry, 407605 The Chinese University of Hong Kong, Shatin, NT, Hong Kong, 999077, China

## Abstract

A facile and catalyst-free method for the α-C­(sp^3^)–H acetalization of cyclic and aliphatic ethers mediated by *N*-bromoamide DBDMH is reported. The reaction proceeds under mild conditions and exhibits a broad substrate scope. Common ethers, such as THF and Et_2_O, as well as a variety of functionalized alcohols, were efficiently transformed, affording the corresponding acetals in high yields. The developed protocol was further applied to the synthesis of the fragrances Cyanophyll and Efetaal, highlighting its practical utility. Mechanistic studies indicate that the impurities in commercial DBDMH such as molecular bromine and acids were crucial for the high reaction efficiency.

## Graphical abstract

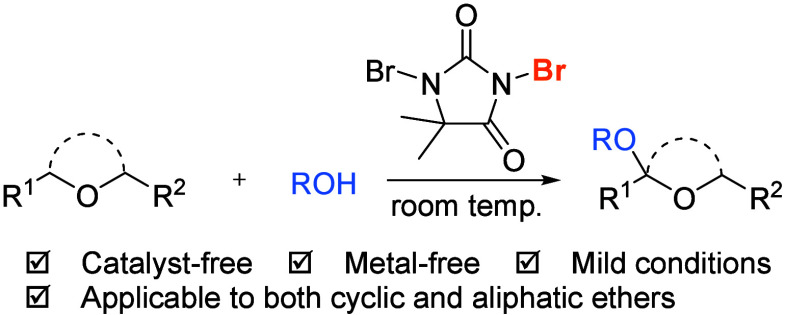

Acetal compounds are widely employed across diverse fields, including pharmaceuticals,^1^ polymeric materials,^2^ and the fragrance industry,^3^ owing to their distinctive chemical properties. Examples such as the PAF receptor antagonist (**1**),^4^ cyanophyll (**2**), and efetaal (**3**) are well-documented for their roles in drug discovery and as fragrances (Scheme 1A).^1,3^ Furthermore, acetals are valued in organic synthesis as important protecting groups for alcohols, owing to their straightforward installation and removal under mild conditions, as exemplified by cyclic acetals such as tetrahydrofuranyl ether.^5^ As a result, the development of efficient and direct acetalization methodologies remains in high demand due to the importance of acetal motifs.

**Scheme 1:**
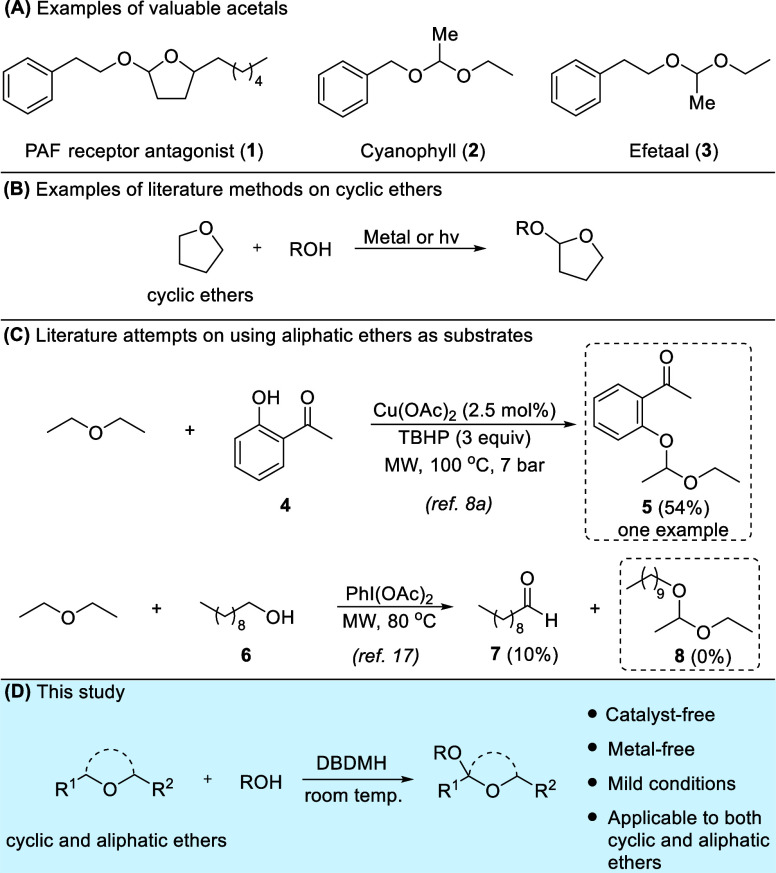
C–H Acetalization of Ethers. (A) Examples of Bioactive Acetals. (B) Examples of Literature Methods on Cyclic Ethers. (C) Examples of Attempts at the C–H Acetalization of Aliphatic Ethers in the Literature. (D) Our Study on the Catalyst-Free C–H Acetalization of Ethers Mediated by DBDMH

Although simple ethers like THF and Et_2_O are typically used as solvents owing to the relative inertness of their C­(sp^3^)–H bonds, they can participate in dehydrogenative coupling with alcohols through C–H activation, a particularly attractive strategy that provides rapid, direct access to acetals from simple building blocks. This methodology exemplifies the broader paradigm of C­(sp^3^)–H functionalization,^6^ a transformative approach in modern organic synthesis that enables traditionally inert C–H bonds to function as direct reactive sites.

This has spurred significant research efforts aimed at developing efficient methodologies that unlock the synthetic potential of simple ethers via C–H bond activation. These approaches focus on the selective activation of traditionally unreactive C–H bonds to facilitate the formation of new C–C and C–X (where X = N or O) bonds.^7^ The installation of THF-acetal protecting groups on alcohols is most commonly achieved through metal-catalyzed processes employing copper,^8^ iron,^9^ rhenium,^10^ cerium,^11^ palladium,^12^ titanium,^13^ vanadium,^14^ or aluminum^15^ catalysts (Scheme 1B). Light-promoted procedures have also been reported.^16^ While these methods cover a range of substrates, they also suffer from some limitations, including the use of expensive reagents and harsh reaction conditions (e.g., elevated temperatures and strongly acidic environments).

Moreover, these methods are generally ineffective for aliphatic ethers, despite their appeal as synthetic targets. A rare example has been reported involving the reaction of diethyl ether with phenol **4**, employing a catalytic amount of copper­(II) acetate and a superstoichiometric quantity of TBHP as the oxidant.^8a^ Under thermal conditions, no reaction was observed; however, employing microwave irradiation at high pressure enabled the formation of acetal product **5** in moderate yields (Scheme 1C). In another case, a stoichiometric hypervalent iodine oxidant proved effective for the C–H acetalization of cyclic ethers. However, when the same reaction system was applied to diethyl ether and decanol **6**, the oxidant proved incompatible with the alcohol. This led to the oxidation of decanol **6** to aldehyde **7**, and the desired acetal product **8** was not observed.^17^ The difficulty in achieving C–H functionalization of aliphatic ethers relative to their cyclic counterparts could be attributed to the unfavorable stereoelectronic effects, which impede both the formation and stabilization of the necessary radical intermediates.^18^

Therefore, the development of a metal-free strategy that operates under mild conditions and exhibits functional group tolerance is highly desirable. As an ongoing research interest in halogenation in our team,^19^ we were intrigued by the possibility of employing halogen sources as both radical initiators and oxidants to access acetals in a single operation. Herein, we disclose a catalyst-free, metal-free and operationally simple strategy for α-C­(sp^3^)–H acetalization of ethers that proceeds under ambient air and moisture and is amenable to gram-scale synthesis (Scheme 1D). This unified method was found to be applicable to both cyclic and aliphatic ethers under ambient conditions. In addition, we demonstrated the facile synthesis of natural compounds cyanophyll (**2**), and efetaal (**3**).

The reaction between 4-nitrobenzyl alcohol (**9a**) and THF was used in our initial study, which was carried out in the absence of light. Various commercially available brominating sources were investigated to evaluate their ability to activate the THF (Table 1). No desired product was detected when using 2,4,4,6-tetrabromo-2,5-cyclohexadienone (TBCO) (entry 1). *N*-bromoacetamide (NBA) gave the desired product **10a** in 10% yield (entry 2). The yield was further improved when using *N*-bromosuccinimide (NBS) or *N*-bromophthalimide (NBP) as the Br source (entries 3–4). To our delight, a 90% yield of **10a** was obtained when the more active Br source 1,3-dibromo-5,5-dimethylhydantoin (DBDMH) was used (entry 5). Poorer yield was obtained when a large excess of DBDMH was used; however, no diacetalization was observed (entry 6). The yield was diminished when reducing the amount of DBDMH (entry 7).

**Table 1:**
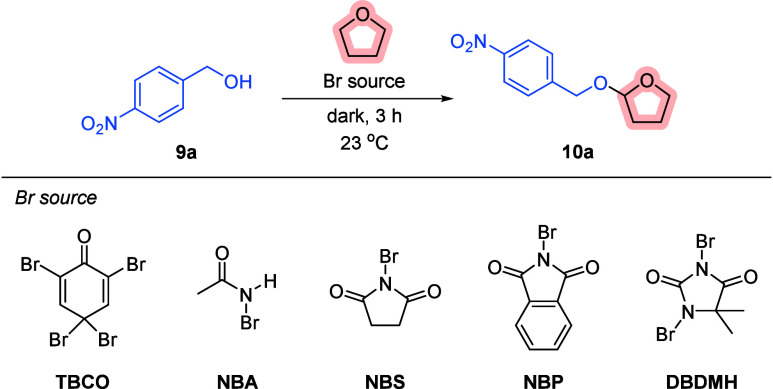
Reaction Optimization^a^

entry	Br source	equivalence	yield (%)
1	TBCO	2.0	0
2	NBA	2.0	10
3	NBS	2.0	25
4	NBP	2.0	30
5	DBDMH	2.0	90 (86)^b^
6	DBDMH	4.0	65
7	DBDMH	1.5	58

a Reactions were carried out with **9a** (0.2 mmol), commercial Br source (0.4 mmol) in stabilizer-free THF (4.0 mL) for 3 h at 23 °C in dark. The yields were measured using ^1^H NMR with CH_2_Br_2_ as the internal standard.

b Isolated yield.

With optimized conditions in hand, we next sought to determine the scope of this reaction (Scheme 2). In general, monosubstituted primary benzyl alcohols performed well and delivered their respective products **10a**–**10j** in good yields. Multisubstituted benzyl alcohols **9k**–**9m** were also examined and the corresponding acetals **10k**–**10m** were obtained smoothly. Next, some bulkier alcohols including secondary (**9n**–**9p**) and tertiary (**9q**) benzylic alcohols were studied. To our delight, the reaction system was found to be compatible, and the respective products **10n**–**10q** were furnished in good-to-excellent yields. Other aliphatic alcohols with longer hydrocarbon chains were then studied, and acetals **10r**–**10t** were synthesized successfully. Under this mild reaction protocol, not only aliphatic alcohols but also cycloalkyl alcohols (**9u**–**9v**) reacted efficiently, affording **10u** and **10v** in 66% and 72% yields, respectively. Diol **9w** was subjected to the optimized conditions and the corresponding monoacetal **10w** was obtained selectively in 92% yield. In addition, when doubling the amount of DBDMH, the diacetal **10x** was furnished in 74% yield. When 2-methyltetrahydrofuran was used instead of THF, the reaction proceeded readily to give the acetal product, as exemplified by the reaction with nonanol to afford product **10y**. Replacing the alcohol with *N*-tosylamine as the nucleophile also allowed the reaction to proceed smoothly, affording **10z** in 80% yield.

**Scheme 2:**
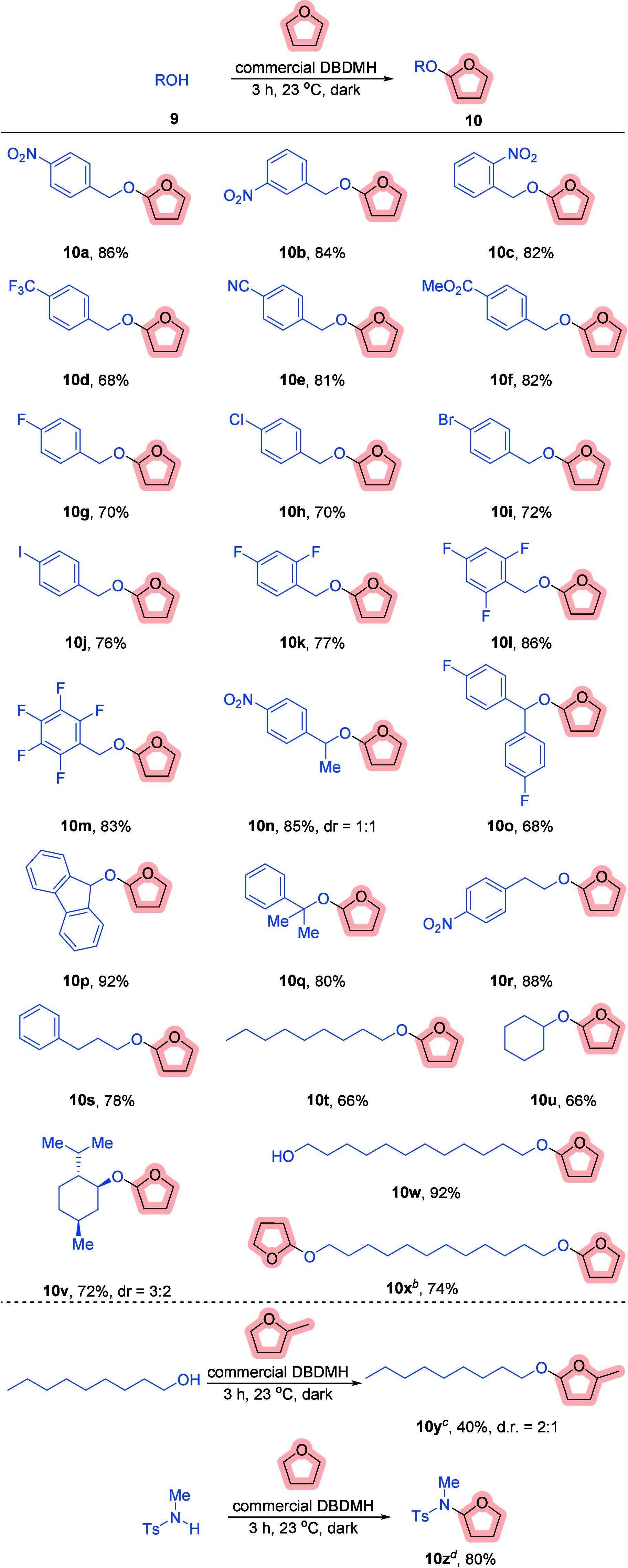
Reaction Scope of Cyclic Ethers^a^

a Reactions were carried out with **9** (0.2 mmol), commercial DBDMH (0.4 mmol) in stabilizer-free THF (4.0 mL) for 3 h at 23 °C in dark. The yields are isolated yields.
b 0.8 mmol of DBDMH was used.
c 2-Methyltetrahydrofuran was used instead of THF.
d TsMeNH was used instead of alcohol.

Next, reactions between diethyl ether and various alcohols such as substituted benzyl alcohol, secondary and tertiary benzylic alcohols, and aliphatic and cyclic alkyl alcohols were examined using the protocol (Scheme 3). Much to our delight, the reactions generally performed well and delivered the products **11a**–**11e** in satisfying yields. It is worth-mentioning that cyanophyll (**2**) and efetaal (**3**) were successfully obtained using this method. They can be used in daily chemical essence formula, especially in soap, detergent, perfume and cosmetics essence formula.^1–4^ To demonstrate the scalability of the present synthetic methodology, a gram-scale experiment was carried out with phenylethanol in diethyl ether, affording efetaal (**3**) in 60% yield.

**Scheme 3:**
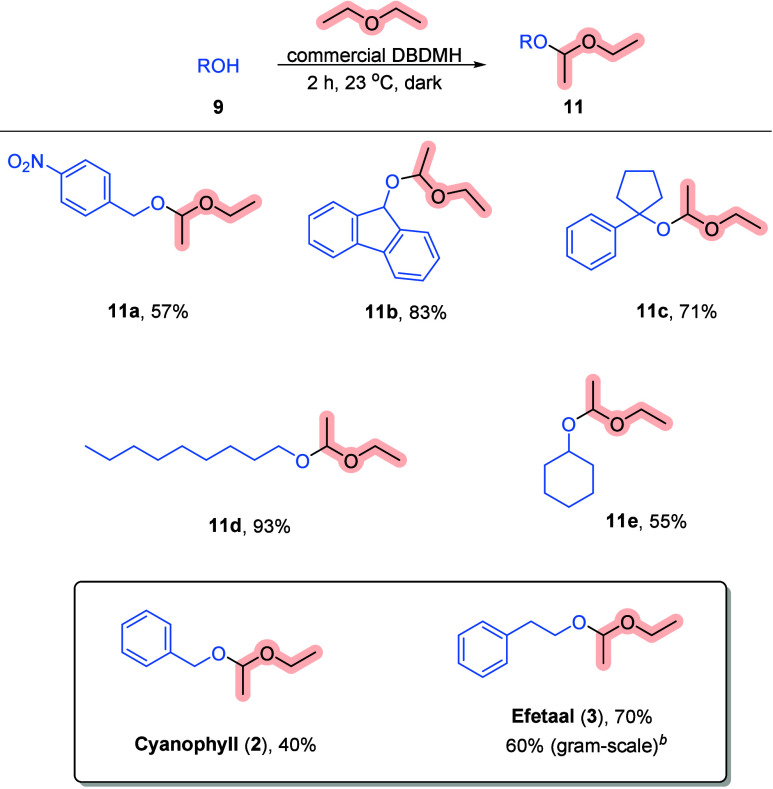
Reaction Scope of Aliphatic Ethers and Synthetic Utilities^a^

a Reactions were carried out with **9** (0.2 mmol), commercial DBDMH (0.4 mmol) in stabilizer-free Et_2_O (4.0 mL) for 2 h at 23 °C in dark. The yields are isolated yields.
b 1.0 g of 2-phenylethanol was used.

Some control experiments were carried out to provide mechanistic insight (Scheme 4). The addition of 2,2,6,6-tetramethylpiperidinyloxy (TEMPO) or butylated hydroxytoluene (BHT) suppressed the formation of the desired product **10a**. However, the BHT-THF adduct **11** was detected by HRMS (SI, Figure S1). These results suggest the involvement of a radical pathway in the DBDMH-promoted acetalization (Scheme 4A).

**Scheme 4:**
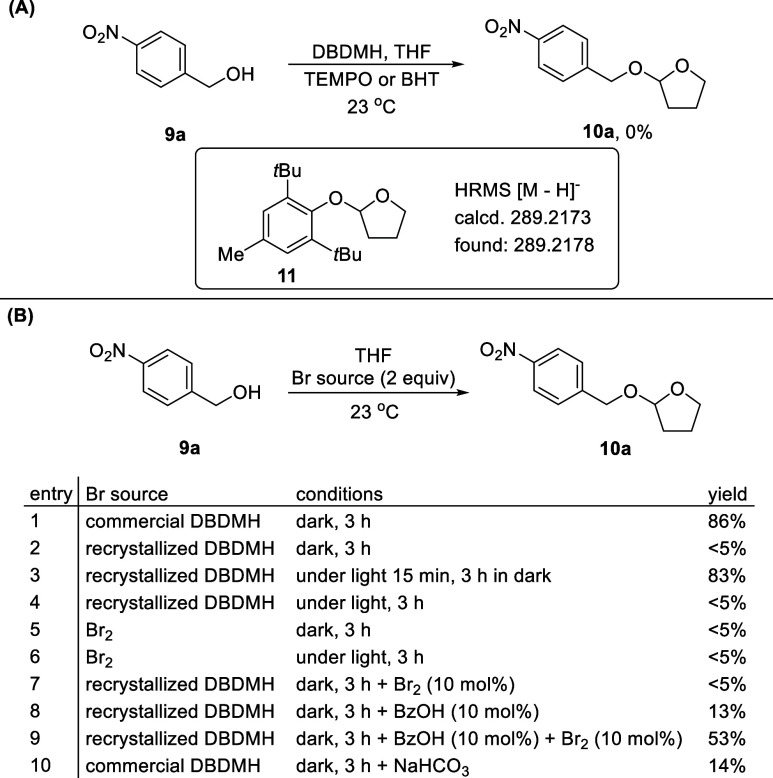
Mechanistic Studies. (A) Study on the Effect of Radical Scavengers. (B) Study on the Effect of Potential Impurities

During the study, it was unexpected to realize that the reaction became sluggish when using recrystallized DBDMH (Scheme 4B, entry 2). However, irradiating the reaction for 15 min under a household fluorescence lamp (15 W), followed by 3 h reaction in the dark, gave **10a** with a yield comparable to that obtained with the commercial bottle of DBDMH (entry 1 vs 3). Prolonging the light irradiation period led to a complex mixture and a very low yield of **10a** (entry 4). We suspected that contamination in the commercial bottle of DBDMH might play an important role in the reaction, and that briefly irradiating recrystallized DBDMH could generate an appropriate amount of the same contamination. *N*-haloamide reagents might decompose to give molecular halogen, which could further react with moisture to give acid impurities.^20^ Indeed, HRMS analysis of the commercial DBDMH revealed the presence of impurities, including HBr, HOBr, and Br_2_ (SI, Figure S2). Thus, control experiments were performed to study the effect of the potential impurities. Employing pure bromine as the Br source resulted in a low product yield, no matter in the absence or presence of light (Scheme 4B, entries 5–6). The reaction with 10 mol % Br_2_ or BzOH (as a mimic of the acid impurity) together with recrystallized DBDMH in the dark was examined, but the desired product was obtained only in low yield (Scheme 4B, entries 7–8). However, using recrystallized DBDMH in combination with the additives (10 mol % Br_2_ and BzOH) led to a significantly improved reaction (Scheme 4B, entry 9 vs 2). On the other hand, the addition of solid NaHCO_3_ as an acid scavenger with commercial DBDMH led to significant yield diminishment (Scheme 4B, entry 10 vs 1). These results indicate that both molecular bromine and acids might be useful in the DBDMH-mediated C­(sp^3^)–H acetalization.

While a more detailed investigation is required to determine the exact reaction mechanism, a plausible mechanism is proposed based on the mechanistic studies described above (Scheme 5). Since both molecular bromine and acid might be involved, one of the possible explanations is that the molecular bromine and acid could form a halogen bond (XB)^21^ and a hydrogen bond complex with DBDMH to give species **A**, and such an interaction could also enhance the reactivity of DBDMH. We believe that the reaction might involve radical abstraction of the α-C­(sp^3^)–H in the ether to give species **B**, together with the generation of hydantoin radical **C**. Radical **C** might also abstract α-C­(sp^3^)–H in the ether to give **B**. Subsequently, radical halogenation of **B** by bromine or DBDMH could yield species **D**.^22^ Next, elimination of bromide in species **D** might generate oxocarbenium **E**, which might then be trapped by an alcohol to give species **F**. Finally, elimination of HBr could give the desired acetal product. HBr might react with DBDMH to regenerate molecular bromine, which could explain why only a catalytic amount of bromine was sufficient.

**Scheme 5:**
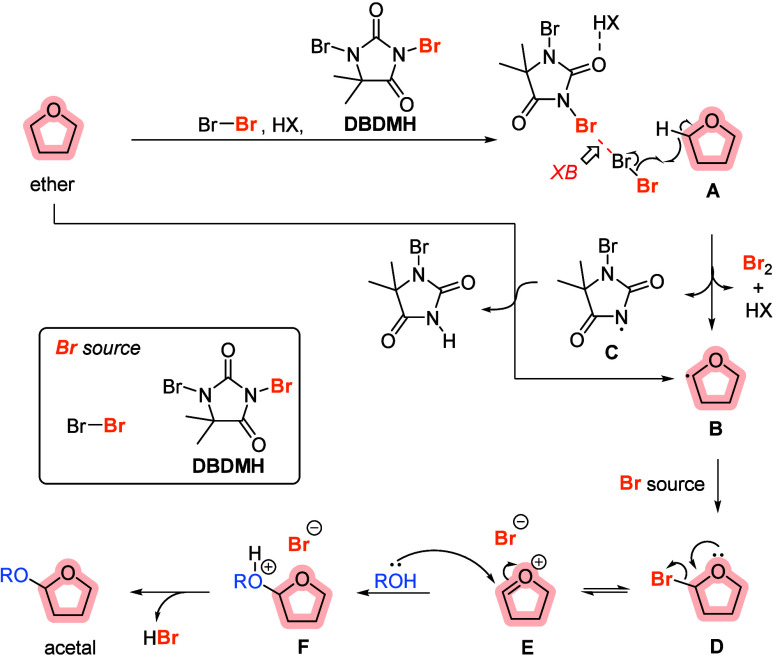
A Plausible Mechanism

In summary, we have developed a catalyst-free, DBDMH-promoted acetalization between ethers and alcohols. The reactions were operated under mild and metal-free conditions. Based upon its operational simplicity and the mild reaction conditions, the current method opens up a new and efficient access to an array of valuable acetals.

## Supplementary Material



## Data Availability

The data underlying this study are available in the published article and its Supporting Information.

## Notes

All data supporting the findings of this study are available within the article, including experimental methods, characterization data and NMR spectra of new compounds (PDF)
