# Supplementary material for: α‑C(sp3)–H Acetalization of Cyclic and Aliphatic Ethers Mediated by N‑Bromoamides
Source: Org Lett. 2026 Jun 24;28(26):8407–12. doi: 10.1021/acs.orglett.6c02125 (PMC13339770; doi:10.1021/acs.orglett.6c02125)
Supplement: Supplementary file 1 [file ol6c02125_si_001.pdf]

Supporting Information

α-C(sp^3)−H Acetalization of Cyclic and Aliphatic Ethers Mediated by *N*-bromoamides

Qingyu Zhang, Yat-Long Cheng, Ying-Yeung Yeung*

Department of Chemistry and State Key Laboratory of Synthetic Chemistry, The Chinese University of Hong Kong, Shatin, NT, Hong Kong, China

Email: yyyeung@cuhk.edu.hk;

Table of Contents

1. General methods and materials S2
2. Mechanistic studies S3
3. General procedure and physical data S5
4. NMR Spectra S17

1. General methods and materials

Commercially available reagents were used as received. The solvents were dried by distillation over the appropriate drying reagents or solvent purification system (Inert PS-MD-7). NMR spectra were recorded on a Bruker AMX500 (500MHz) spectrometer or a Bruker AMX400 (400 MHz) spectrometer in $CDCl_3$, acetone-d_6 or methanol-d_4. Chemical shifts are reported as δ values relative to internal chloroform (δ 7.26 for ^1H NMR and 77.00 for ^{13}C NMR), acetone-d_6 (δ 2.05 for ^1H NMR and 29.84 for ^{13}C NMR), methanol-d_4 (δ 3.31 for ^1H NMR and 49.00 for ^{13}C NMR). High resolution mass spectra were obtained on a Finnigan/MAT 95XL-T spectrometer (ionization mode: ESI or APCI). Analytical thin layer chromatography (TLC) was performed with Merck pre-coated TLC plates, silica gel 60F-254, layer thickness 0.25 mm. Column chromatography was performed on Merck 60 (0.040-0.063 mm) mesh silica gel. All reactions sensitive to air or moisture were carried out under an argon atmosphere in dry and freshly distilled solvents under anhydrous conditions, unless otherwise noted

2. Mechanistic studies

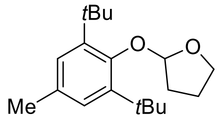

HRMS (APCI) calcd for $C_{19}H_{29}O_2^-$ [M-H]$^-$: 289.2173; found: 289.2178.

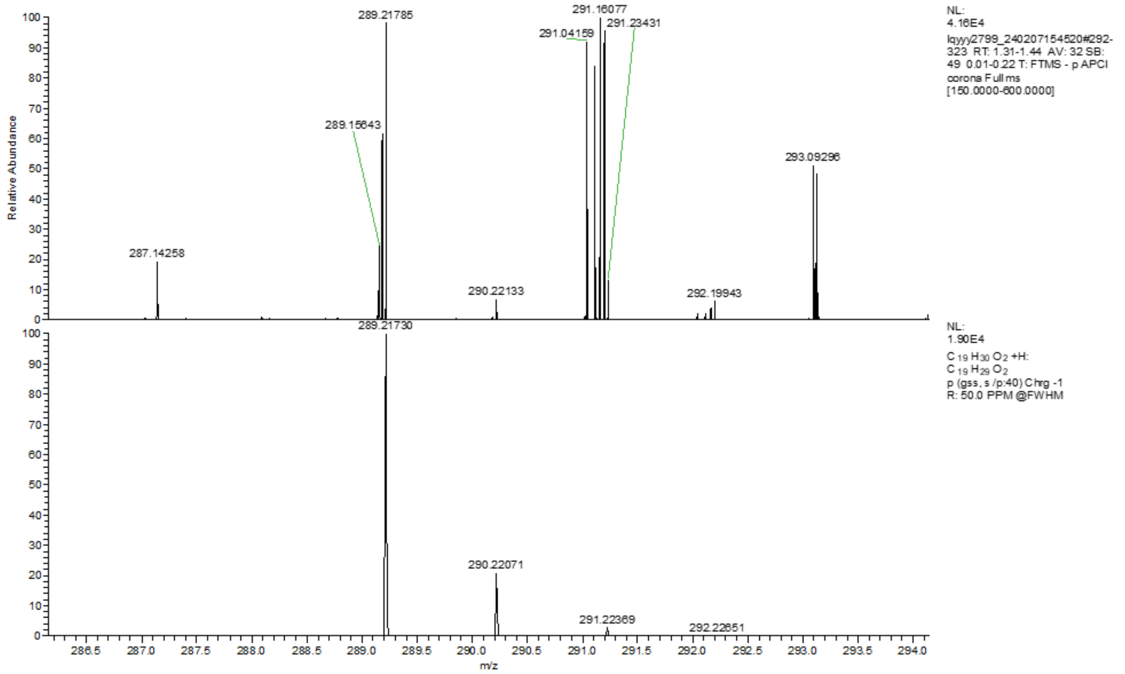

Figure S1. HRMS analysis of the BHT-THF adduct **11**.

HBr: **HRMS (ESI)** calcd for Br^- [M-H]$^-$: 78.9189; found: 78.9189.

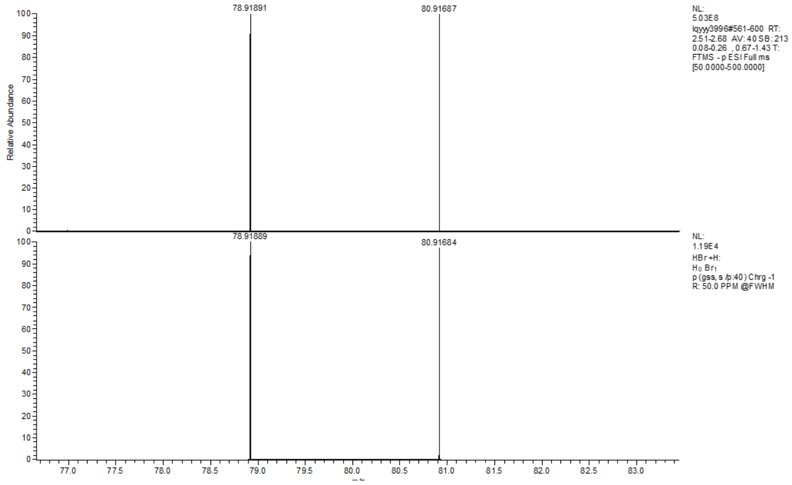

HOBr: **HRMS (ESI)** calcd for OBr^- [M-H]$^-$: 94.9138; found: 94.9138.

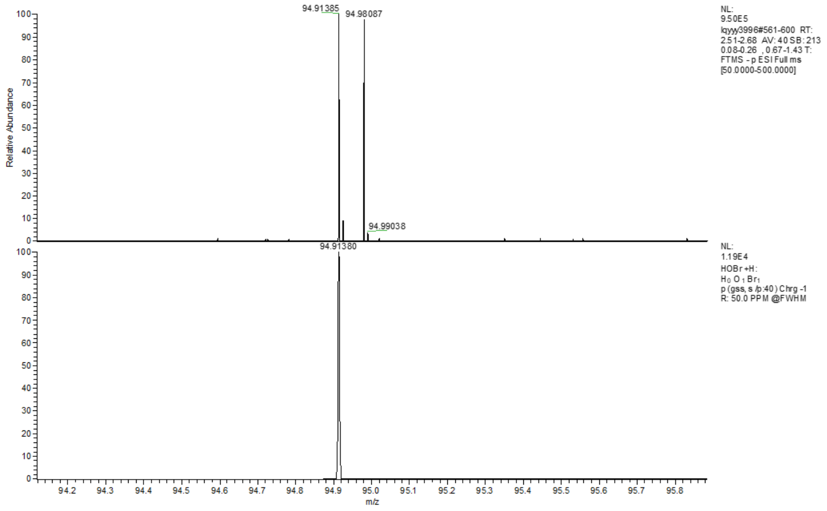

Br_2: **HRMS (ESI)** calcd for Br_2^- [M]$^-$: 159.8352; found: 159.8352.

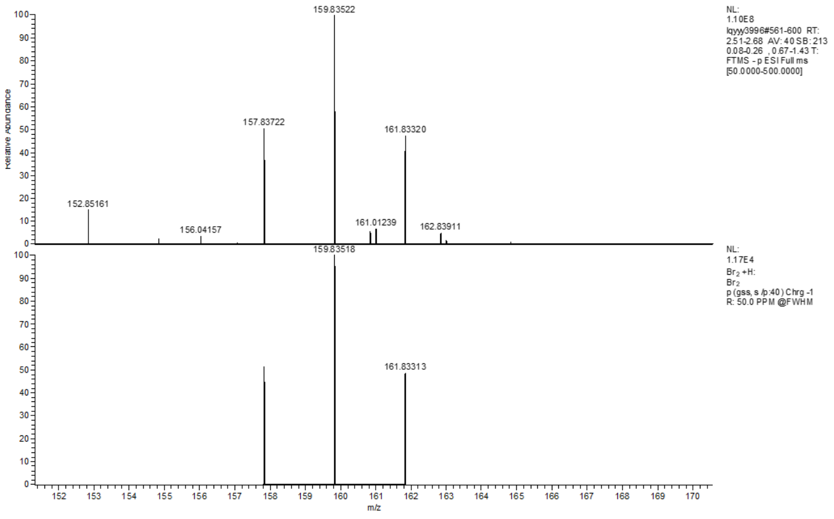

Figure S2. HRMS analysis on the impurities in the commercial sample of DBDMH.

3. General procedure and physical data

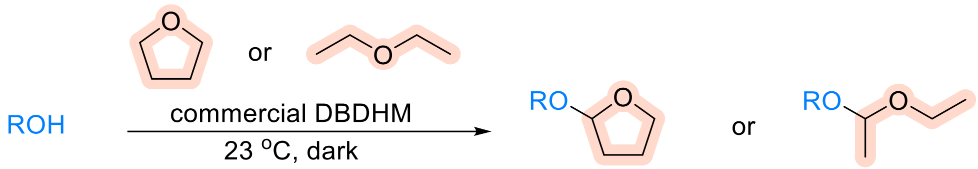

9 10 11

General Procedure A: To a solution of alcohol **9** (0.2 mmol, 1.0 equiv) in stabilizer-free THF or Et_2O (4.0 mL, 0.05 M) was added commercial DBDMH (0.4 mmol, 2.0 equiv) at 23 °C in the absence of light for 3 h. Saturated aqueous Na_2SO_3 solution was then added to the reaction. The mixture was extracted with EtOAc. The combined organic layers was dried over anhydrous Na_2SO_4, filtered, and concentrated under reduced pressure. The residue was purified by column chromatography to give the product **10** or **11**.

Note: Product yields were found to vary slightly between different commercial bottles. More reproducible results were achieved by following General Procedure B.

General Procedure B: To a solution of alcohol **9** (0.2 mmol, 1.0 equiv) in stabilizer-free THF or Et_2O (4.0 mL, 0.05 M) was added recrystallized DBDMH (0.4 mmol, 2.0 equiv) at 23 °C. The solution was stirred under a household fluorescence lamp (15 W) for 15 min followed by 3 h in the dark. Saturated aqueous Na_2SO_3 solution was then added to the reaction. The mixture was extracted with EtOAc. The combined organic layers was dried over anhydrous Na_2SO_4, filtered, and concentrated under reduced pressure. The residue was purified by column chromatography to give the product **10** or **11**.

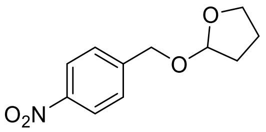

2-((4-nitrobenzyl)oxy)tetrahydrofuran (10a)
Isolated by column chromatography: Hexane/EtOAc = 10/1
Yield: 86% (38.4 mg)
Physical state: pale yellow oil
^{1}H NMR (500 MHz, Chloroform-*d*) δ 8.17 (d, *J* = 8.8 Hz, 2H), 7.48 (d, *J* = 8.6 Hz, 2H), 5.21 (dd, *J* = 4.1, 2.0 Hz, 1H), 4.79 (d, *J* = 13.4 Hz, 1H), 4.57 (d, *J* = 13.3 Hz, 1H), 3.95 – 3.86 (m, 2H), 2.09 – 1.82 (m, 4H).
^{13}C NMR (126 MHz, Chloroform-*d*) δ 147.1, 146.2, 127.7, 123.5, 103.6, 67.5, 67.2, 32.3, 23.4.
HRMS (ESI) calcd for $C_{11}H_{13}NO_4Na^+$ $[M+Na]^+$: 246.0737; found: 246.0735.

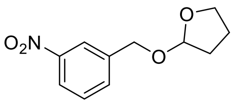

2-((3-nitrobenzyl)oxy)tetrahydrofuran (10b)
Isolated by column chromatography: Hexane/EtOAc = 10/1
Yield: 84% (37.6 mg)
Physical state: pale yellow oil
^{1}H NMR (500 MHz, Chloroform-*d*) δ 8.20 (s, 1H), 8.11 (d, *J* = 8.1 Hz, 1H), 7.64 (d, *J* = 7.5 Hz, 1H), 7.49 (t, *J* = 7.9 Hz, 1H), 5.23 (dd, *J* = 4.3, 1.9 Hz, 1H), 4.79 (d, *J* = 12.6 Hz, 1H), 4.56 (d, *J* = 12.6 Hz, 1H), 3.97 – 3.86 (m, 2H), 2.08 – 1.83 (m, 4H).
^{13}C NMR (126 MHz, Chloroform-*d*) δ 148.3, 140.8, 133.4, 129.2, 122.3, 122.3, 103.6, 67.5, 67.3, 32.4, 23.4.
HRMS (ESI) calcd for $C_{11}H_{13}NO_4Na^+$ $[M+Na]^+$: 246.0737; found: 246.0735.

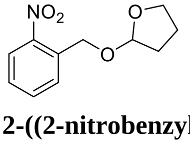

2-((2-nitrobenzyl)oxy)tetrahydrofuran (10c)
Isolated by column chromatography: Hexane/EtOAc = 10/1
Yield: 82% (36.7 mg)
Physical state: pale yellow oil
^{1}H NMR (500 MHz, Chloroform-*d*) δ 8.02 (d, *J* = 8.1 Hz, 1H), 7.72 (d, *J* = 7.7 Hz, 1H), 7.61 (t, *J* = 7.6 Hz, 1H), 7.41 (t, *J* = 7.6 Hz, 1H), 5.28 – 5.21 (m, 1H), 5.05 (d, *J* = 14.9 Hz, 1H), 4.86 (d, *J* = 14.9 Hz, 1H), 3.98 – 3.83 (m, 2H), 2.10 – 1.81 (m, 4H).
^{13}C NMR (126 MHz, Chloroform-*d*) δ 147.5, 135.0, 133.3, 128.9, 127.8, 124.5, 103.9, 67.3, 65.7, 32.3, 23.4.
HRMS (ESI) calcd for $C_{11}H_{13}NO_4Na^+$ $[M+Na]^+$: 246.0737; found: 246.0735.

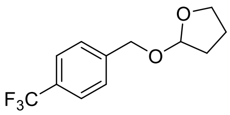

2-((4-(trifluoromethyl)benzyl)oxy)tetrahydrofuran (10d)

Isolated by column chromatography: Hexane/EtOAc = 10/1

Yield: 68% (33.6 mg)

Physical state: yellow oil

^{1}H NMR (500 MHz, Chloroform-*d*) δ 7.59 (d, *J* = 7.9 Hz, 2H), 7.45 (d, *J* = 7.9 Hz, 2H), 5.22 (dd, *J* = 4.0, 2.2 Hz, 1H), 4.76 (d, *J* = 12.6 Hz, 1H), 4.53 (d, *J* = 12.6 Hz, 1H), 3.98 – 3.88 (m, 2H), 2.09 – 1.83 (m, 4H);

^{13}C NMR (126 MHz, Chloroform-*d*) δ 142.6 (d, *J* = 1.3 Hz), 129.6 (q, *J* = 32.3 Hz), 127.7, 125.3 (q, *J* = 3.9 Hz), 103.4, 68.0, 67.2, 32.4, 23.4; ^{19}F NMR (471 MHz, Chloroform-*d*) δ -62.48;

HRMS (ESI) calcd for $C_{12}H_{13}F_3O_2Na^+$ [M+Na]$^+$: 269.0760; found: 269.0757.

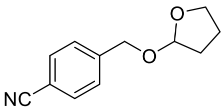

4-(((tetrahydrofuran-2-yl)oxy)methyl)benzonitrile (10e)

Isolated by column chromatography: Hexane/EtOAc = 10/1

Yield: 81% (32.9 mg)

Physical state: colorless oil

^{1}H NMR (500 MHz, Chloroform-*d*) δ 7.61 (d, *J* = 8.2 Hz, 2H), 7.43 (d, *J* = 8.0 Hz, 2H), 5.22 – 5.17 (m, 1H), 4.74 (d, *J* = 13.1 Hz, 1H), 4.52 (d, *J* = 13.2 Hz, 1H), 3.95 – 3.85 (m, 2H), 2.07 – 1.82 (m, 4H).

^{13}C NMR (126 MHz, Chloroform-*d*) δ 144.1, 132.1, 127.8, 118.8, 111.0, 103.5, 67.7, 67.2, 32.3, 23.4.

HRMS (ESI) calcd for $C_{12}H_{13}NO_2Na^+$ [M+Na]$^+$: 226.0838; found: 226.0837.

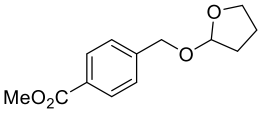

methyl 4-(((tetrahydrofuran-2-yl)oxy)methyl)benzoate (10f)

Isolated by column chromatography: Hexane/EtOAc = 10/1

Yield: 82% (38.8 mg)

Physical state: colorless oil

^{1}H NMR (500 MHz, Chloroform-*d*) δ 8.00 (d, *J* = 8.3 Hz, 2H), 7.39 (d, *J* = 8.3 Hz, 2H), 5.21 (dd, *J* = 4.4, 1.9 Hz, 1H), 4.75 (d, *J* = 12.7 Hz, 1H), 4.53 (d, *J* = 12.8 Hz, 1H), 3.96 – 3.86 (m, 5H), 2.08 – 1.82 (m, 4H).

^{13}C NMR (126 MHz, Chloroform-*d*) δ 166.9, 143.8, 129.6, 129.1, 127.2, 103.4, 68.1, 67.1, 52.0, 32.3, 23.4.

HRMS (ESI) calcd for $C_{13}H_{16}O_4Na^+$ [M+Na]$^+$: 259.0941; found: 259.0939.

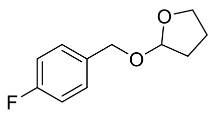

2-((4-fluorobenzyl)oxy)tetrahydrofuran (10g)

Isolated by column chromatography: Hexane/EtOAc = 10/1

Yield: 70% (27.5 mg)

Physical state: pale yellow oil;

^{1}H NMR (500 MHz, Chloroform-*d*) δ 7.34 – 7.28 (m, 2H), 7.06 – 6.98 (m, 2H), 5.20 (t, *J* = 3.1 Hz, 1H), 4.67 (d, *J* = 11.7 Hz, 1H), 4.44 (d, *J* = 11.7 Hz, 1H), 3.97 – 3.87 (m, 2H), 2.07 – 1.80 (m, 4H);

^{13}C NMR (126 MHz, Chloroform-*d*) δ 162.2 (d, *J* = 245.3 Hz), 134.1 (d, *J* = 3.2 Hz), 129.5 (d, *J* = 8.1 Hz), 115.1 (d, *J* = 21.4 Hz), 103.0, 68.0, 67.0, 32.3, 23.4.

^{19}F NMR (471 MHz, Chloroform-*d*) δ -115.12 – -115.23 (m).

HRMS (ESI) calcd for $C_{11}H_{13}FO_2Na^+$ $[M+Na]^+$: 219.0792; found: 219.0791.

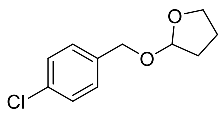

2-((4-chlorobenzyl)oxy)tetrahydrofuran (10h)

Isolated by column chromatography: Hexane/EtOAc = 10/1

Yield: 70% (29.6 mg)

Physical state: colorless oil

^{1}H NMR (500 MHz, Chloroform-*d*) δ 7.35 – 7.27 (m, 4H), 5.22 (t, *J* = 3.1 Hz, 1H), 4.69 (d, *J* = 12.0 Hz, 1H), 4.46 (d, *J* = 12.1 Hz, 1H), 3.99 – 3.89 (m, 2H), 2.10 – 1.84 (m, 4H).

^{13}C NMR (126 MHz, Chloroform-*d*) δ 136.9, 133.2, 129.1, 128.4, 103.1, 67.9, 67.1, 32.3, 23.4.

HRMS (ESI) calcd for $C_{11}H_{13}ClO_2Na^+$ $[M+Na]^+$: 235.0496; found: 235.0496.

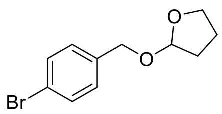

2-((4-bromobenzyl)oxy)tetrahydrofuran (10i)

Isolated by column chromatography: Hexane/EtOAc = 10/1

Yield: 72% (40.1 mg)

Physical state: pale yellow oil

^{1}H NMR (500 MHz, Chloroform-*d*) δ 7.45 (d, *J* = 8.0 Hz, 2H), 7.21 (d, *J* = 8.0 Hz, 2H), 5.19 (t, *J* = 3.1 Hz, 1H), 4.65 (d, *J* = 12.1 Hz, 1H), 4.42 (d, *J* = 12.2 Hz, 1H), 3.98 – 3.85 (m, 2H), 2.07 – 1.79 (m, 4H).

^{13}C NMR (126 MHz, Chloroform-*d*) δ 137.4, 131.4, 129.4, 121.3, 103.1, 67.9, 67.1, 32.3, 23.4.

HRMS (ESI) calcd for $C_{11}H_{13}BrO_2Na^+$ $[M+Na]^+$: 278.9991; found: 278.9991.

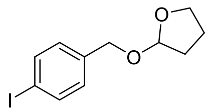

2-((4-iodobenzyl)oxy)tetrahydrofuran (10j)

Isolated by column chromatography: Hexane/EtOAc = 10/1

Yield: 76% (46.3 mg)

Physical state: colorless oil

^{1}H NMR (500 MHz, Chloroform-*d*) δ 7.66 (d, *J* = 8.2 Hz, 2H), 7.08 (d, *J* = 8.3 Hz, 2H), 5.19 (t, *J* = 3.0 Hz, 1H), 4.64 (d, *J* = 12.2 Hz, 1H), 4.41 (d, *J* = 12.1 Hz, 1H), 3.96 – 3.86 (m, 2H), 2.07 – 1.81 (m, 4H).

^{13}C NMR (126 MHz, Chloroform-*d*) δ 138.0, 137.3, 129.6, 103.1, 92.8, 68.0, 67.1, 32.3, 23.4.

HRMS (ESI) calcd for $C_{11}H_{13}IO_2Na^+$ $[M+Na]^+$: 326.9852; found: 326.9853.

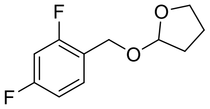

2-((2,4-difluorobenzyl)oxy)tetrahydrofuran (10k)

Isolated by column chromatography: Hexane/EtOAc = 10/1

Yield: 77% (33.0 mg)

Physical state: colorless oil;

^{1}H NMR (500 MHz, Chloroform-*d*) δ 7.36 (td, *J* = 8.4, 6.5 Hz, 1H), 6.85 (td, *J* = 8.2, 1.8 Hz, 1H), 6.79 (td, *J* = 9.5, 2.6 Hz, 1H), 5.21 (t, *J* = 3.1 Hz, 1H), 4.70 (d, *J* = 11.9 Hz, 1H), 4.50 (d, *J* = 12.0 Hz, 1H), 3.98 – 3.85 (m, 2H), 2.07 – 1.80 (m, 4H);

^{13}C NMR (126 MHz, Chloroform-*d*) δ 162.6 (dd, *J* = 207.8, 11.9 Hz), 160.7 (dd, *J* = 209.6, 12.0 Hz), 131.1 (dd, *J* = 9.7, 6.0 Hz), 121.4 (dd, *J* = 15.0, 3.7 Hz), 111.0 (dd, *J* = 21.0, 3.7 Hz), 103.7 (t, *J* = 25.4 Hz), 103.3, 67.1, 61.9 (d, *J* = 3.3 Hz), 32.3, 23.3;

^{19}F NMR (471 MHz, Chloroform-*d*) δ -111.17 (p, *J* = 7.8 Hz), -114.49 (q, *J* = 8.5 Hz);

HRMS (ESI) calcd for $C_{11}H_{12}F_2O_2Na^+$ $[M+Na]^+$: 237.0698; found: 237.0697.

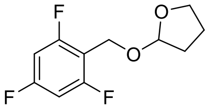

2-((2,4,6-trifluorobenzyl)oxy)tetrahydrofuran (10l)

Isolated by column chromatography: Hexane/EtOAc = 10/1

Yield: 86% (39.9 mg)

Physical state: colorless oil

^{1}H NMR (500 MHz, Chloroform-*d*) δ 6.70 – 6.60 (m, 2H), 5.21 (dd, *J* = 4.5, 1.5 Hz, 1H), 4.70 (dt, *J* = 11.1, 1.6 Hz, 1H), 4.48 (dt, *J* = 11.1, 1.4 Hz, 1H), 3.96 – 3.85 (m, 2H), 2.03 – 1.78 (m, 4H).

^{13}C NMR (126 MHz, Chloroform-*d*) δ 162.6 (dt, *J* = 250.7, 15.1 Hz), 162.2 (ddd, *J* = 252.0, 15.0, 10.9 Hz), 110.5 (td, *J* = 19.9, 4.6 Hz), 103.3 (d, *J* = 17.4 Hz), 100.8 – 99.2 (m), 66.9 (t), 55.5 (t, *J* = 3.4 Hz), 32.3 (t, *J* = 2.8 Hz), 23.2 (t).

^{19}F NMR (471 MHz, Chloroform-*d*) δ -107.73 – -107.84 (m), -112.10 (t, *J* = 7.1 Hz).

HRMS (ESI) calcd for $C_{11}H_{11}F_3O_2Na^+$ $[M+Na]^+$: 255.0603; found: 255.0603.

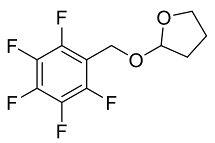

2-((perfluorophenyl)methoxy)tetrahydrofuran (10m)

Isolated by column chromatography: Hexane/EtOAc = 10/1

Yield: 83% (44.5 mg)

Physical state: colorless oil

^{1}H NMR (500 MHz, Chloroform-*d*) δ 5.21 (d, *J* = 4.5 Hz, 1H), 4.76 (d, *J* = 11.2 Hz, 1H), 4.54 (d, *J* = 11.2 Hz, 1H), 3.90 (t, *J* = 6.9 Hz, 2H), 2.06 – 1.76 (m, 4H);

^{13}C NMR (126 MHz, Chloroform-*d*) δ 146.9 – 144.2 (m), 142.6 – 139.7 (m), 138.7 – 135.9 (m), 112.9 – 110.0 (m), 103.7, 67.2, 55.5, 32.4, 23.1;

^{19}F NMR (471 MHz, Chloroform-*d*) δ -143.16 (dd, *J* = 22.6, 8.7 Hz), -154.47 (t, *J* = 20.7 Hz), -162.39 (td, *J* = 22.0, 8.7 Hz);

HRMS (ESI) calcd for $C_{11}H_9F_5O_2Na^+$ $[M+Na]^+$: 291.0415; found: 291.0414.

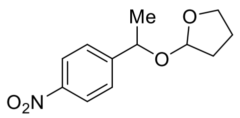

2-(1-(4-nitrophenyl)ethoxy)tetrahydrofuran (10n)

Isolated by column chromatography: Hexane/EtOAc = 10/1

Yield: 85% (40.3 mg)

Physical state: yellow oil

^{1}H NMR (500 MHz, Chloroform-*d*) δ 8.18 (dd, *J* = 10.1, 8.7 Hz, 2H), 7.47 (d, *J* = 8.4 Hz, 2H), 5.37 – 5.32 (m, 0.5H), 4.95 – 4.85 (m, 1H), 4.79 (q, *J* = 6.6 Hz, 0.5H), 4.00 – 3.84 (m, 1H), 3.75 – 3.56 (m, 1H), 2.10 – 1.79 (m, 4H), 1.42 (dd, *J* = 6.6, 2.2 Hz, 3H).

^{13}C NMR (126 MHz, Chloroform-*d*) δ 152.9, 151.5, 147.2, 146.8, 127.1, 126.4, 123.7, 123.5, 102.9, 101.4, 73.4, 72.4, 67.0, 67.0, 32.6, 32.3, 24.3, 23.4, 23.4, 22.8.

HRMS (ESI) calcd for $C_{12}H_{15}NO_4Na^+$ $[M+Na]^+$: 260.0893; found: 260.0893.

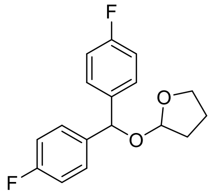

2-(bis(4-fluorophenyl)methoxy)tetrahydrofuran (10o)

Isolated by column chromatography: Hexane/EtOAc = 10/1

Yield: 68% (39.3 mg)

Physical state: colorless oil

^{1}H NMR (500 MHz, Chloroform-*d*) δ 7.31 (dd, *J* = 8.5, 5.5 Hz, 2H), 7.23 (dd, *J* = 8.5, 5.5 Hz, 2H), 7.04 (t, *J* = 8.7 Hz, 2H), 6.98 (t, *J* = 8.7 Hz, 2H), 5.74 (s, 1H), 5.14 (d, *J* = 4.4 Hz, 1H), 3.97 – 3.83 (m, 2H), 2.12 – 1.99 (m, 2H), 1.95 – 1.82 (m, 2H).

^{13}C NMR (126 MHz, Chloroform-*d*) δ 162.1 (dd, *J* = 245.8, 48.4 Hz), 137.8 (dd, *J* = 195.7, 3.1 Hz), 128.8 (dd, *J* = 113.6, 8.1 Hz), 115.2 (dd, *J* = 53.3, 21.4 Hz), 101.1, 77.1, 67.1, 32.3, 23.4.
^{19}F NMR (471 MHz, Chloroform-*d*) δ -114.62 – -114.74 (m), -115.62 – -115.74 (m).
HRMS (ESI) calcd for $C_{17}H_{16}F_2O_2Na^+$ [M+Na]$^+$: 313.1011; found: 313.1009.

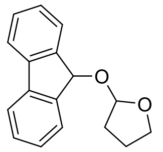

2-((9H-fluoren-9-yl)oxy)tetrahydrofuran (10p)
Isolated by column chromatography: Hexane/EtOAc = 10/1
Yield: 92% (46.4 mg)
Physical state: pale yellow oil
^{1}H NMR (500 MHz, Chloroform-*d*) δ 7.72 – 7.60 (m, 4H), 7.39 (t, *J* = 7.5 Hz, 2H), 7.36 – 7.28 (m, 2H), 5.74 – 5.67 (m, 2H), 4.16 – 4.08 (m, 1H), 4.04 – 3.95 (m, 1H), 2.16 – 1.85 (m, 4H).
^{13}C NMR (126 MHz, Chloroform-*d*) δ 144.1 (d, *J* = 36.0 Hz), 140.4 (d, *J* = 13.9 Hz), 129.0 – 128.5 (m), 127.4 (dd, *J* = 23.7, 5.1 Hz), 125.6 (dd, *J* = 85.9, 21.6 Hz), 119.8 (dd, *J* = 21.6, 17.2 Hz), 104.1 (d, *J* = 8.7 Hz), 78.9 (d, *J* = 7.8 Hz), 67.1 (dd, *J* = 36.5, 32.8 Hz), 33.2 – 32.3 (m), 23.8 – 22.8 (m).
HRMS (ESI) calcd for $C_{17}H_{16}O_2Na^+$ [M+Na]$^+$: 275.1042; found: 275.1039.

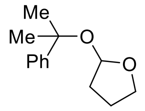

2-((2-phenylpropan-2-yl)oxy)tetrahydrofuran (10q)
Isolated by column chromatography: Hexane/EtOAc = 20/1
Yield: 80% (32.9 mg)
Physical state: pale yellow oil
^{1}H NMR (500 MHz, Acetone-*d*$_6$) δ 7.47 – 7.42 (m, 2H), 7.36 – 7.30 (m, 2H), 7.25 – 7.20 (m, 1H), 5.11 (dd, *J* = 4.5, 2.6 Hz, 1H), 3.84 (td, *J* = 7.9, 6.2 Hz, 1H), 3.69 (td, *J* = 7.8, 5.8 Hz, 1H), 2.02 – 1.92 (m, 1H), 1.88 – 1.81 (m, 2H), 1.78 – 1.69 (m, 1H), 1.62 (s, 3H), 1.46 (s, 3H).
^{13}C NMR (126 MHz, Acetone-*d*$_6$) δ 148.6, 128.8, 127.4, 126.5, 100.3, 77.7, 67.1, 34.0, 31.9, 27.9, 24.4.
HRMS (ESI) calcd for $C_{13}H_{18}O_2Na^+$ [M+Na]$^+$: 229.1199; found: 229.1198.

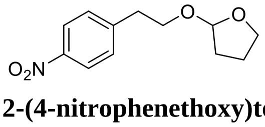

2-(4-nitrophenethoxy)tetrahydrofuran (10r)
Isolated by column chromatography: Hexane/EtOAc = 10/1
Yield: 88% (41.8 mg)
Physical state: pale yellow oil
^{1}H NMR (500 MHz, Chloroform-*d*) δ 8.13 (d, *J* = 8.7 Hz, 2H), 7.37 (d, *J* = 8.8 Hz, 2H), 5.11 – 5.04 (m, 1H), 3.91 (dt, *J* = 9.9, 6.7 Hz, 1H), 3.85 – 3.77 (m, 1H), 3.77 – 3.70 (m, 1H), 3.63 (dt, *J* = 9.9, 6.5 Hz, 1H), 2.96 (t, *J* = 6.6 Hz, 2H), 1.96 – 1.75 (m, 4H).
^{13}C NMR (126 MHz, Chloroform-*d*) δ 147.4, 146.5, 129.7, 123.4, 103.8, 66.9, 66.6, 36.1, 32.3, 23.4.
HRMS (ESI) calcd for $C_{12}H_{15}NO_4Na^+$ [M+Na]$^+$: 260.0893; found: 260.0893.

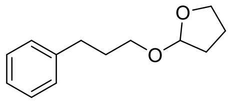

2-(3-phenylpropoxy)tetrahydrofuran (10s)

Isolated by column chromatography: Hexane/EtOAc = 10/1

Yield: 78% (32.2 mg)

Physical state: pale yellow oil

^{1}H NMR (500 MHz, Chloroform-*d*) δ 7.31 – 7.27 (m, 2H), 7.22 – 7.18 (m, 3H), 5.12 (dd, *J* = 4.5, 1.9 Hz, 1H), 3.94 – 3.85 (m, 2H), 3.70 (dt, *J* = 9.7, 6.6 Hz, 1H), 3.40 (dt, *J* = 9.7, 6.5 Hz, 1H), 2.69 (dd, *J* = 8.8, 6.7 Hz, 2H), 2.05 – 1.97 (m, 1H), 1.96 – 1.87 (m, 4H), 1.86 – 1.78 (m, 1H).

^{13}C NMR (126 MHz, Chloroform-*d*) δ 142.0, 128.4, 128.2, 125.7, 103.8, 66.8, 66.4, 32.4, 32.3, 31.3, 23.5.

HRMS (ESI) calcd for $C_{13}H_{18}O_2Na^+$ [M+Na]$^+$: 229.1199; found: 229.1198.

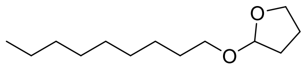

2-(nonyloxy)tetrahydrofuran (10t)

Isolated by column chromatography: Hexane/EtOAc = 20/1

Yield: 66% (28.3 mg)

Physical state: colorless oil

^{1}H NMR (500 MHz, Acetone-*d*$_6$) δ 5.04 (dd, *J* = 4.7, 1.3 Hz, 1H), 3.83 – 3.73 (m, 2H), 3.59 (dt, *J* = 9.5, 6.7 Hz, 1H), 3.31 (dt, *J* = 9.5, 6.5 Hz, 1H), 1.94 – 1.81 (m, 2H), 1.82 – 1.73 (m, 2H), 1.55 – 1.46 (m, 2H), 1.34 – 1.27 (m, 12H), 0.88 (t, *J* = 6.5 Hz, 3H).

^{13}C NMR (126 MHz, Acetone-*d*$_6$) δ 104.3, 67.4, 66.9, 32.9, 32.6, 30.6, 30.3, 30.2, 30.0, 27.0, 24.2, 23.3, 14.4.

HRMS (ESI) calcd for $C_{13}H_{26}O_2Na^+$ [M+Na]$^+$: 237.1825; found: 237.1824.

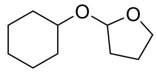

2-(cyclohexyloxy)tetrahydrofuran (10u)

Isolated by column chromatography: Hexane/EtOAc = 20/1

Yield: 66% (22.5 mg)

Physical state: colorless oil

^{1}H NMR (500 MHz, Acetone-*d*$_6$) δ 5.25 – 5.19 (m, 1H), 3.82 – 3.71 (m, 2H), 3.55 – 3.45 (m, 1H), 1.95 – 1.88 (m, 1H), 1.86 – 1.79 (m, 3H), 1.78 – 1.72 (m, 2H), 1.70 – 1.64 (m, 2H), 1.53 – 1.46 (m, 1H), 1.32 – 1.18 (m, 5H).

^{13}C NMR (126 MHz, Acetone-*d*$_6$) δ 102.4, 74.6, 66.7, 34.6, 33.2, 32.7, 26.5, 24.8, 24.7, 24.2.

HRMS (ESI) calcd for $C_{10}H_{18}O_2Na^+$ [M+Na]$^+$: 193.1199; found: 193.1199.

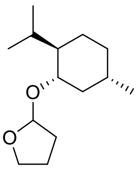

2-(((1S,2R,5S)-2-isopropyl-5-methylcyclohexyl)oxy)tetrahydrofuran (10v)

Isolated by column chromatography: Hexane/EtOAc = 20/1

Yield: 72% (32.6 mg)

Physical state: pale yellow oil

^{1}H NMR (500 MHz, Acetone-d_6) δ 5.30 – 5.13 (m, 1H), 3.88 – 3.71 (m, 2H), 3.45 – 3.21 (m, 1H), 2.22 – 2.07 (m, 2H), 1.99 – 1.72 (m, 4H), 1.69 – 1.56 (m, 2H), 1.45 – 1.31 (m, 1H), 1.17 – 1.08 (m, 1H), 1.06 – 0.95 (m, 1H), 0.92 – 0.84 (m, 7H), 0.80 – 0.70 (m, 4H).

^{13}C NMR (126 MHz, Acetone-d_6) δ 106.0, 100.1, 79.2, 74.0, 67.0, 66.7, 49.7, 49.0, 44.4, 40.8, 35.4, 35.3, 33.3, 33.2, 32.3, 32.2, 26.4, 26.1, 24.3, 24.2, 24.1, 23.9, 22.7, 21.4, 21.4, 16.7, 16.0.

HRMS (ESI) calcd for $C_{14}H_{26}O_2Na^+$ [M+Na]$^+$: 249.1825; found: 249.1824.

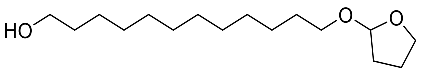

12-((tetrahydrofuran-2-yl)oxy)dodecan-1-ol (10w)

Isolated by column chromatography: Hexane/EtOAc = 5/1

Yield: 92% (50.1 mg)

Physical state: yellow oil

^{1}H NMR (500 MHz, Acetone-d_6) δ 5.06 – 5.02 (m, 1H), 3.77 (t, J = 6.7 Hz, 2H), 3.59 (dt, J = 9.6, 6.7 Hz, 1H), 3.54 – 3.49 (m, 2H), 3.39 (t, J = 5.3 Hz, 1H), 3.31 (dt, J = 9.5, 6.5 Hz, 1H), 1.96 – 1.82 (m, 2H), 1.81 – 1.72 (m, 2H), 1.54 – 1.46 (m, 4H), 1.34 – 1.28 (m, 16H).

^{13}C NMR (126 MHz, Acetone-d_6) δ 104.3, 67.4, 66.9, 62.5, 33.8, 32.9, 30.5, 30.4, 30.4, 30.4, 30.3, 27.0, 26.7, 24.2.

HRMS (ESI) calcd for $C_{16}H_{32}O_3Na^+$ [M+Na]$^+$: 295.2244; found: 295.2246.

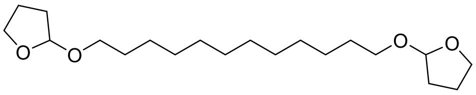

1,12-bis((tetrahydrofuran-2-yl)oxy)dodecane (10x)

Isolated by column chromatography: Hexane/EtOAc = 20/1

Yield: 74% (50.5 mg)

Physical state: yellow oil

^{1}H NMR (500 MHz, Acetone-d_6) δ 5.05 – 5.02 (m, 2H), 3.77 (t, J = 6.7 Hz, 4H), 3.59 (dt, J = 9.5, 6.6 Hz, 2H), 3.31 (dt, J = 9.5, 6.5 Hz, 2H), 1.95 – 1.83 (m, 4H), 1.81 – 1.73 (m, 4H), 1.54 – 1.48 (m, 4H), 1.34 – 1.28 (m, 16H).

^{13}C NMR (126 MHz, Acetone-d_6) δ 104.3, 67.4, 66.9, 32.9, 30.5, 30.4, 30.3, 30.2, 27.0, 24.2.

HRMS (ESI) calcd for $C_{20}H_{38}O_4Na^+$ [M+Na]$^+$: 365.2662; found: 365.2662.

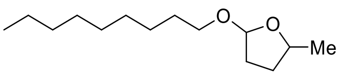

2-methyl-5-(nonyloxy)tetrahydrofuran (10y)

Isolated by column chromatography: Hexane/EtOAc = 100/1

Yield: 40% (18.2 mg)

Physical state: pale yellow oil

^{1}H NMR (400 MHz, Acetone-*d*$_6$) δ 5.04 (dd, *J* = 5.0, 2.0 Hz, 1H), 4.15 – 4.02 (m, 1H), 3.66 – 3.54 (m, 1H), 3.35 – 3.24 (m, 1H), 2.03 – 1.94 (m, 2H), 1.78 – 1.70 (m, 1H), 1.60 – 1.44 (m, 3H), 1.32 – 1.27 (m, 12H), 1.15 (d, *J* = 6.1 Hz, 3H), 0.87 (t, *J* = 7.2 Hz, 3H).

^{13}C NMR (101 MHz, Acetone-*d*$_6$) δ 104.6, 74.1, 67.5, 33.1, 32.6, 32.0, 30.6, 30.3, 30.2, 27.0, 23.3, 21.2, 14.4.

HRMS (ESI) calcd for $C_{14}H_{28}O_2Na^+$ [M+Na]$^+$: 251.1982; found: 251.1980.

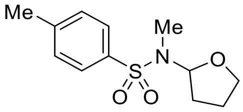

2N,4-dimethyl-N-(tetrahydrofuran-2-yl)benzenesulfonamide (10z)

Isolated by column chromatography: Hexane/EtOAc = 5/1

Yield: 80% (40.6 mg)

Physical state: colorless oil

^{1}H NMR (400 MHz, Acetone-*d*$_6$) δ 7.74 (d, *J* = 8.4 Hz, 2H), 7.38 (d, *J* = 8.0 Hz, 2H), 5.81 (dd, *J* = 7.3, 4.6 Hz, 1H), 3.83 – 3.77 (m, 1H), 3.69 – 3.59 (m, 1H), 2.64 (s, 3H), 2.41 (s, 3H), 2.17 – 2.09 (m, 1H), 1.93 – 1.84 (m, 3H).

^{13}C NMR (101 MHz, Acetone-*d*$_6$) δ 144.0, 137.4, 130.2, 128.6, 88.8, 69.1, 29.8, 28.0, 25.8, 21.4.

HRMS (ESI) calcd for $C_{12}H_{17}NO_3SNa^+$ [M+Na]$^+$: 278.0821; found: 278.0819.

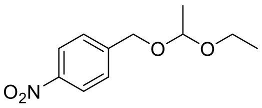

1-((1-ethoxyethoxy)methyl)-4-nitrobenzene (11a)

Isolated by column chromatography: Hexane/EtOAc = 10/1

Yield: 57% (25.7 mg)

Physical state: pale yellow oil

^{1}H NMR (500 MHz, Acetone-*d*$_6$) δ 8.25 – 8.19 (m, 2H), 7.68 – 7.62 (m, 2H), 4.87 (q, *J* = 5.3 Hz, 1H), 4.79 (d, *J* = 13.6 Hz, 1H), 4.68 (d, *J* = 13.6 Hz, 1H), 3.67 (dq, *J* = 9.5, 7.1 Hz, 1H), 3.52 (dq, *J* = 9.5, 7.0 Hz, 1H), 1.33 (d, *J* = 5.3 Hz, 3H), 1.14 (t, *J* = 7.0 Hz, 3H).

^{13}C NMR (126 MHz, Acetone-*d*$_6$) δ 148.0, 148.0, 128.7, 124.1, 100.4, 66.1, 61.6, 20.2, 15.6.

HRMS (ESI) calcd for $C_{11}H_{15}NO_4Na^+$ [M+Na]$^+$: 248.0893; found: 248.0894.

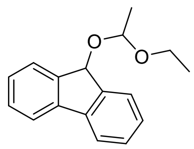

9-(1-ethoxyethoxy)-9H-fluorene (11b)

Isolated by column chromatography: Hexane/EtOAc = 10/1

Yield: 83% (42.3 mg)

Physical state: colorless oil;

^{1}H NMR (500 MHz, Acetone-*d*$_6$) δ 7.75 (d, *J* = 7.3 Hz, 2H), 7.66 (dd, *J* = 12.1, 7.4 Hz, 2H), 7.39 (t, *J* = 7.5 Hz, 2H), 7.35 – 7.29 (m, 2H), 5.69 (s, 1H), 5.23 (q, *J* = 5.3 Hz, 1H), 3.75 – 3.68 (m, 1H), 3.68 – 3.60 (m, 1H), 1.38 (d, *J* = 5.2 Hz, 3H), 1.20 (t, *J* = 7.0 Hz, 3H).

^{13}C NMR (126 MHz, Acetone-*d*$_6$) δ 145.6, 145.3, 141.2, 129.6, 129.6, 128.3, 128.3, 126.7, 126.4, 120.7, 120.6, 100.9, 79.1, 61.1, 21.2, 15.7.

HRMS (ESI) calcd for $C_{17}H_{18}O_2Na^+$ [M+Na]$^+$: 277.1199; found: 277.1198.

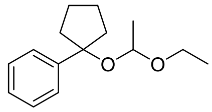

(1-(1-ethoxyethoxy)cyclopentyl)benzene (11c)

Isolated by column chromatography: Hexane/EtOAc = 10/1

Yield: 71% (33.1 mg)

Physical state: pale yellow oil;

^{1}H NMR (500 MHz, Acetone-*d*$_6$) δ 7.49 – 7.43 (m, 2H), 7.37 – 7.31 (m, 2H), 7.29 – 7.23 (m, 1H), 4.45 (q, *J* = 5.3 Hz, 1H), 3.33 – 3.19 (m, 2H), 2.46 – 2.33 (m, 1H), 2.27 – 2.15 (m, 1H), 2.01 – 1.91 (m, 2H), 1.90 – 1.81 (m, 1H), 1.78 – 1.63 (m, 3H), 1.04 (d, *J* = 5.3 Hz, 3H), 0.98 (t, *J* = 7.0 Hz, 3H).

^{13}C NMR (126 MHz, Acetone-*d*$_6$) δ 144.9, 128.8, 128.0, 127.9, 96.2, 89.1, 59.2, 40.4, 36.1, 23.5, 23.4, 21.6, 15.7.

HRMS (ESI) calcd for $C_{15}H_{22}O_2Na^+$ [M+Na]$^+$: 257.1512; found: 257.1511.

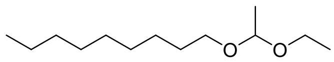

1-(1-ethoxyethoxy)nonane (11d)

Isolated by column chromatography: Hexane/EtOAc = 20/1

Yield: 93% (40.2 mg)

Physical state: colorless oil;

^{1}H NMR (500 MHz, Acetone-*d*$_6$) δ 4.64 (q, *J* = 5.3 Hz, 1H), 3.64 – 3.51 (m, 2H), 3.47 – 3.35 (m, 2H), 1.56 – 1.49 (m, 2H), 1.34 – 1.26 (m, 12H), 1.20 (d, *J* = 5.3 Hz, 3H), 1.12 (t, *J* = 7.1 Hz, 3H), 0.88 (t, *J* = 6.6 Hz, 3H).

^{13}C NMR (126 MHz, Acetone-*d*$_6$) δ 100.1, 65.6, 61.0, 32.6, 30.7, 30.3, 30.2, 30.0, 27.0, 23.3, 20.3, 15.7, 14.4.

HRMS (ESI) calcd for $C_{13}H_{28}O_2Na^+$ [M+Na]$^+$: 239.1982; found: 239.1980.

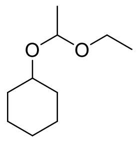

(1-ethoxyethoxy)cyclohexane (11e)

Isolated by column chromatography: Hexane/EtOAc = 20/1

Yield: 55% (19.0 mg)

Physical state: colorless oil;

^{1}H NMR (500 MHz, Acetone-*d*$_6$) δ 4.74 (q, *J* = 5.3 Hz, 1H), 3.63 – 3.55 (m, 1H), 3.54 – 3.48 (m, 1H), 3.47 – 3.38 (m, 1H), 1.84 – 1.80 (m, 2H), 1.72 – 1.68 (m, 2H), 1.31 – 1.24 (m, 6H), 1.20 (d, *J* = 5.3 Hz, 3H), 1.11 (t, *J* = 7.0 Hz, 3H).

^{13}C NMR (101 MHz, Acetone-*d*$_6$) δ 98.7, 74.4, 60.3, 34.1, 33.4, 26.5, 21.4, 15.7.

HRMS (ESI) calcd for $C_{10}H_{20}O_2Na^+$ $[M+Na]^+$: 195.1356; found: 195.1356.

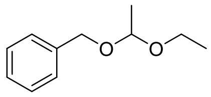

((1-ethoxyethoxy)methyl)benzene (2)

Isolated by column chromatography: Hexane/EtOAc = 10/1

Yield: 40% (14.4 mg)

Physical state: pale yellow oil

^{1}H NMR (500 MHz, Acetone-*d*$_6$) δ 7.38 – 7.31 (m, 4H), 7.29 – 7.24 (m, 1H), 4.80 (q, *J* = 5.3 Hz, 1H), 4.63 (d, *J* = 12.0 Hz, 1H), 4.51 (d, *J* = 11.9 Hz, 1H), 3.65 (dq, *J* = 9.4, 7.1 Hz, 1H), 3.50 (dq, *J* = 9.5, 7.0 Hz, 1H), 1.29 (d, *J* = 5.3 Hz, 3H), 1.15 (t, *J* = 7.0 Hz, 3H).

^{13}C NMR (126 MHz, Acetone-*d*$_6$) δ 140.0, 129.0, 128.3, 128.0, 99.9, 67.5, 61.2, 20.3, 15.7.

HRMS (ESI) calcd for $C_{11}H_{16}O_2Na^+$ $[M+Na]^+$: 203.1042; found: 203.1043.

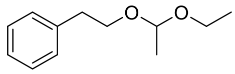

(2-(1-ethoxyethoxy)ethyl)benzene (3)

Isolated by column chromatography: Hexane/EtOAc = 10/1

Yield: 70% (27.2 mg)

For the scale-up reaction, 50 mol% of 2,6-lutidine was added. (1.0 g 2-phenylethanol was used, got **3** 948 mg, the yield was 60%)

Physical state: pale yellow oil

^{1}H NMR (500 MHz, Acetone-*d*$_6$) δ 7.30 – 7.23 (m, 4H), 7.21 – 7.17 (m, 1H), 4.65 (q, *J* = 5.3 Hz, 1H), 3.75 (dt, *J* = 9.5, 7.0 Hz, 1H), 3.62 (dt, *J* = 9.4, 7.0 Hz, 1H), 3.50 (dq, *J* = 9.4, 7.1 Hz, 1H), 3.36 (dq, *J* = 9.4, 7.0 Hz, 1H), 2.83 (t, *J* = 7.0 Hz, 2H), 1.19 (d, *J* = 5.3 Hz, 3H), 1.07 (t, *J* = 7.0 Hz, 3H).

^{13}C NMR (126 MHz, Acetone-*d*$_6$) δ 140.4, 129.8, 129.0, 126.8, 100.1, 66.6, 61.1, 37.2, 20.2, 15.6.

HRMS (ESI) calcd for $C_{12}H_{18}O_2Na^+$ $[M+Na]^+$: 217.1199; found: 217.1199.

4. NMR Spectra

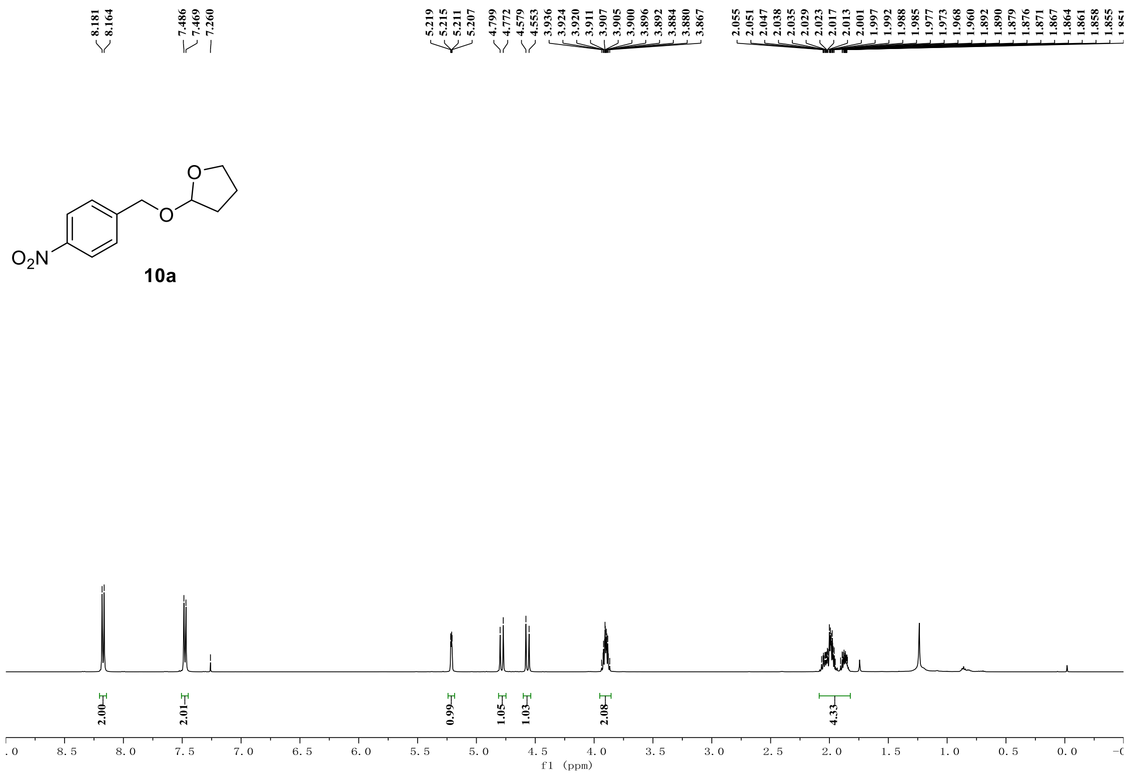

^1H NMR was recorded on Bruker 500 MHz; Solvent: $CDCl_3$

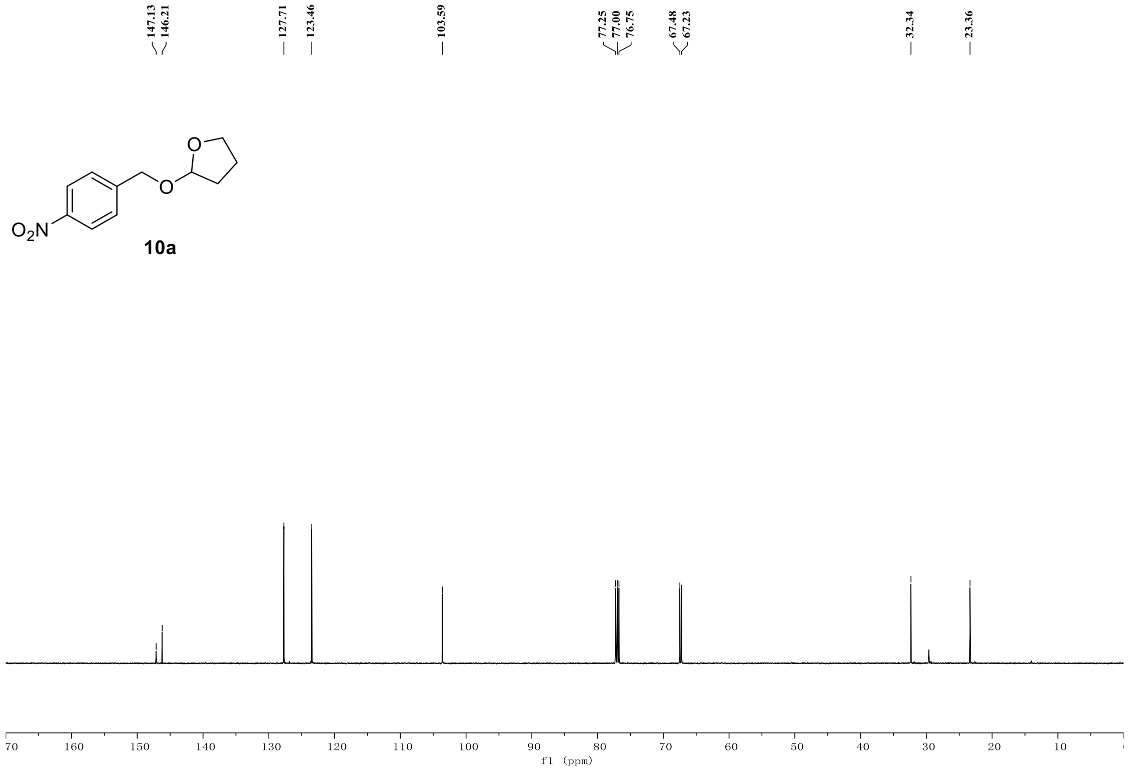

^{13}C NMR was recorded on Bruker 126 MHz; Solvent: $CDCl_3$

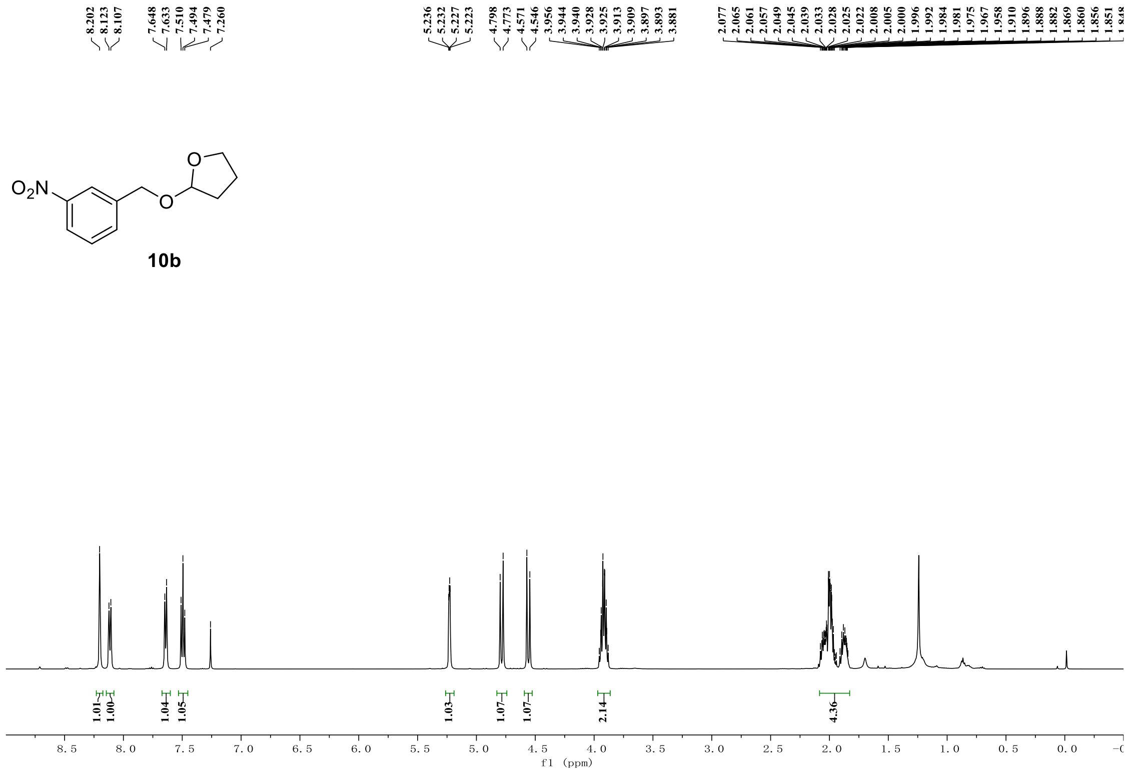

10b

^{1}H NMR was recorded on Bruker 500 MHz; Solvent: $CDCl_3$

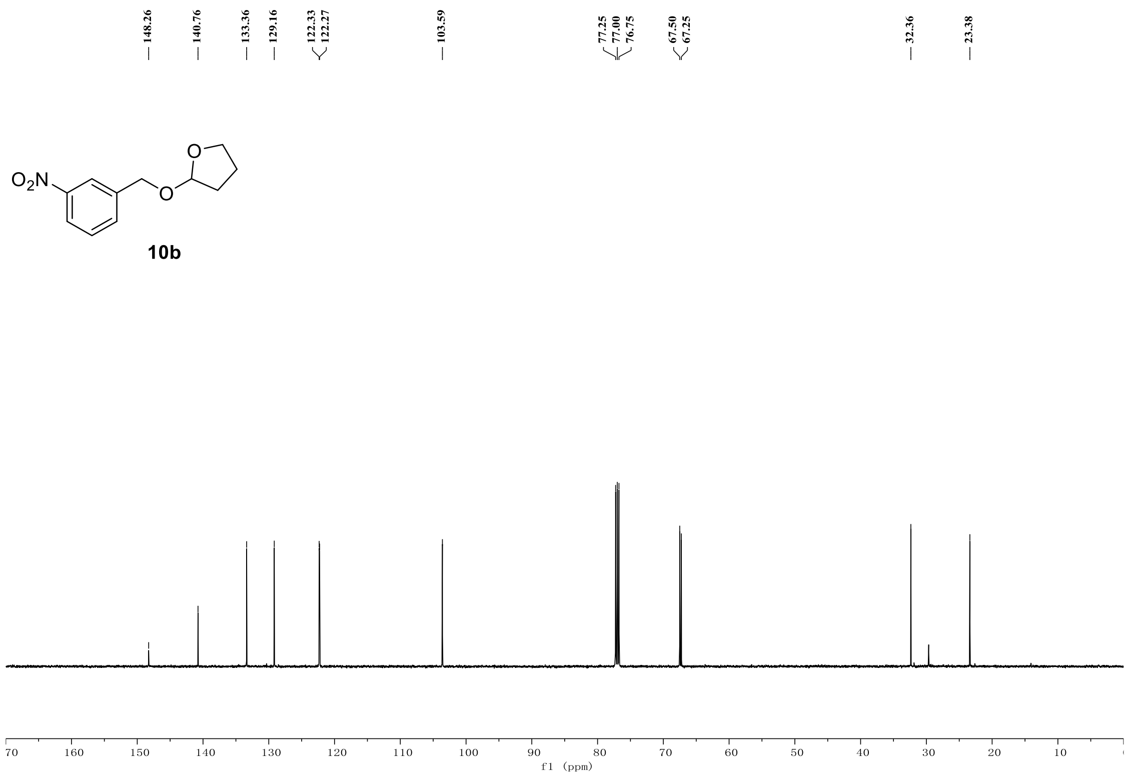

10b

^{13}C NMR was recorded on Bruker 126 MHz; Solvent: $CDCl_3$

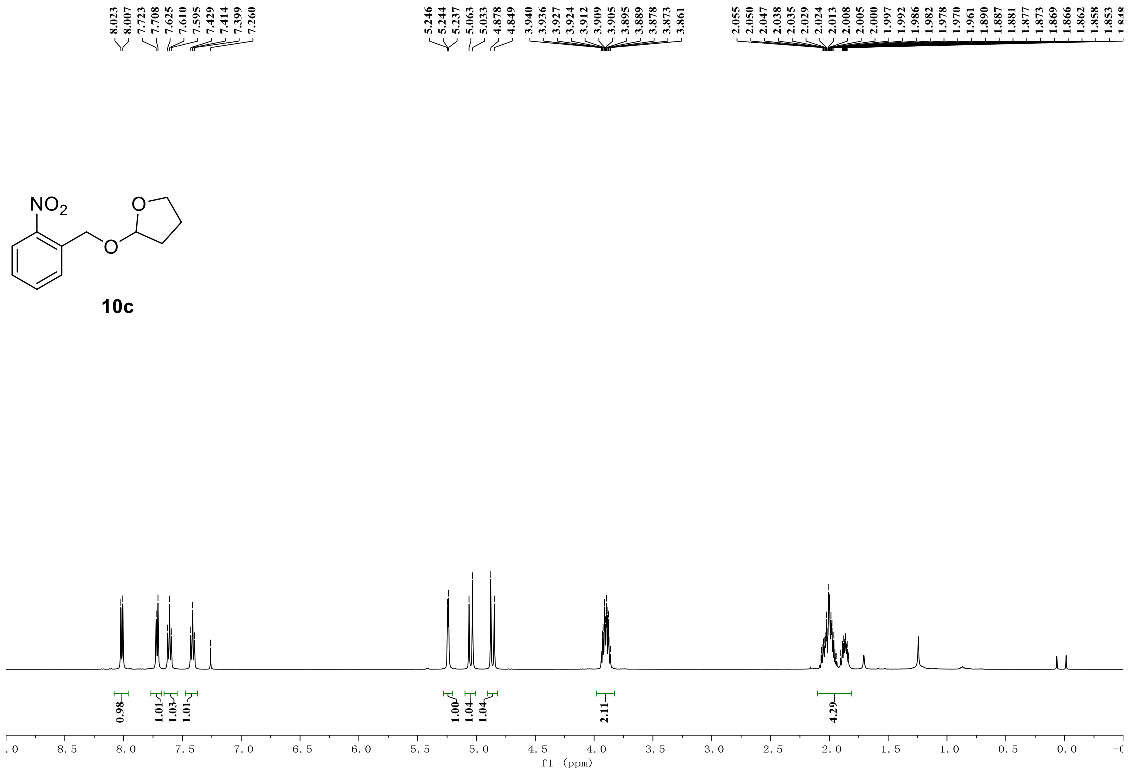

^{1}H NMR was recorded on Bruker 500 MHz; Solvent: $CDCl_3$

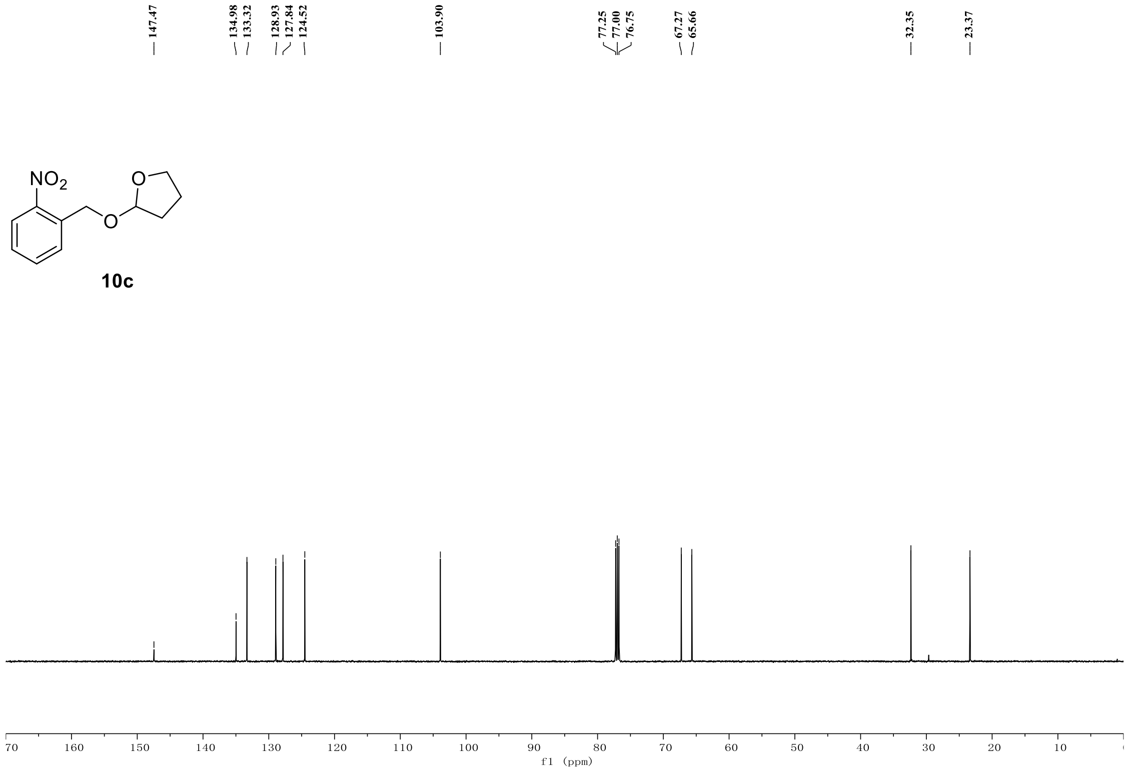

^{13}C NMR was recorded on Bruker 126 MHz; Solvent: $CDCl_3$

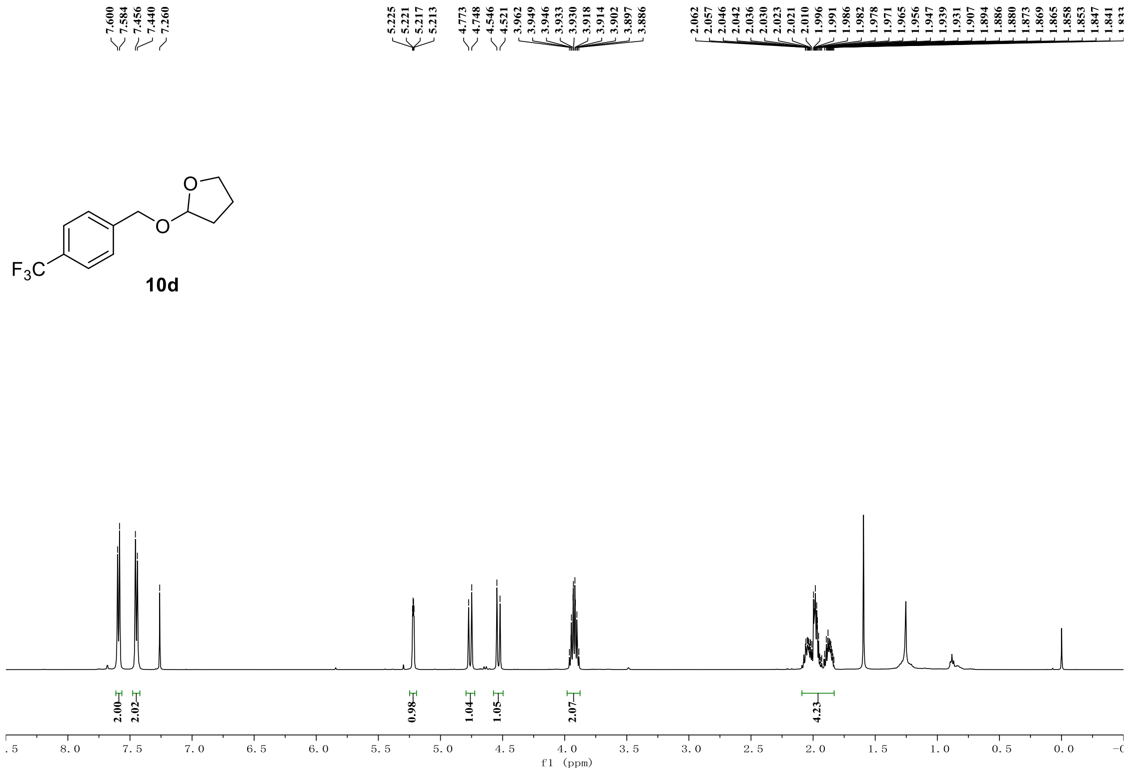

^{1}H NMR was recorded on Bruker 500 MHz; Solvent: $CDCl_3$

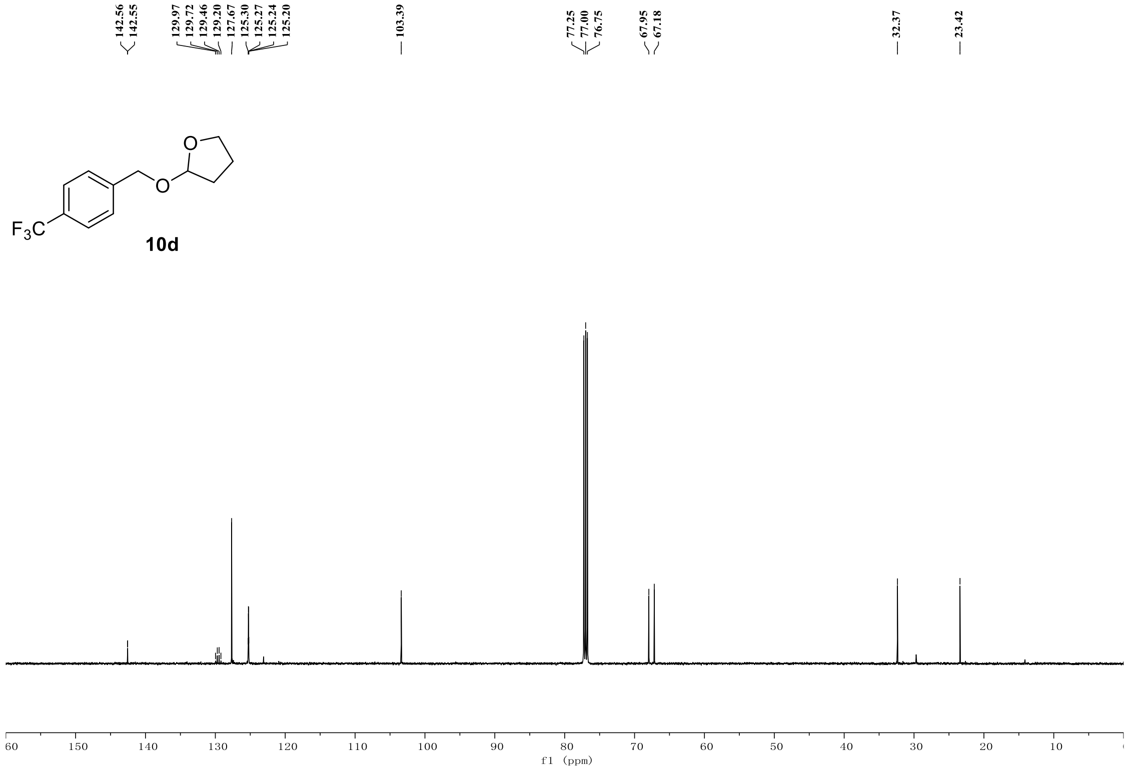

^{13}C NMR was recorded on Bruker 126 MHz; Solvent: $CDCl_3$

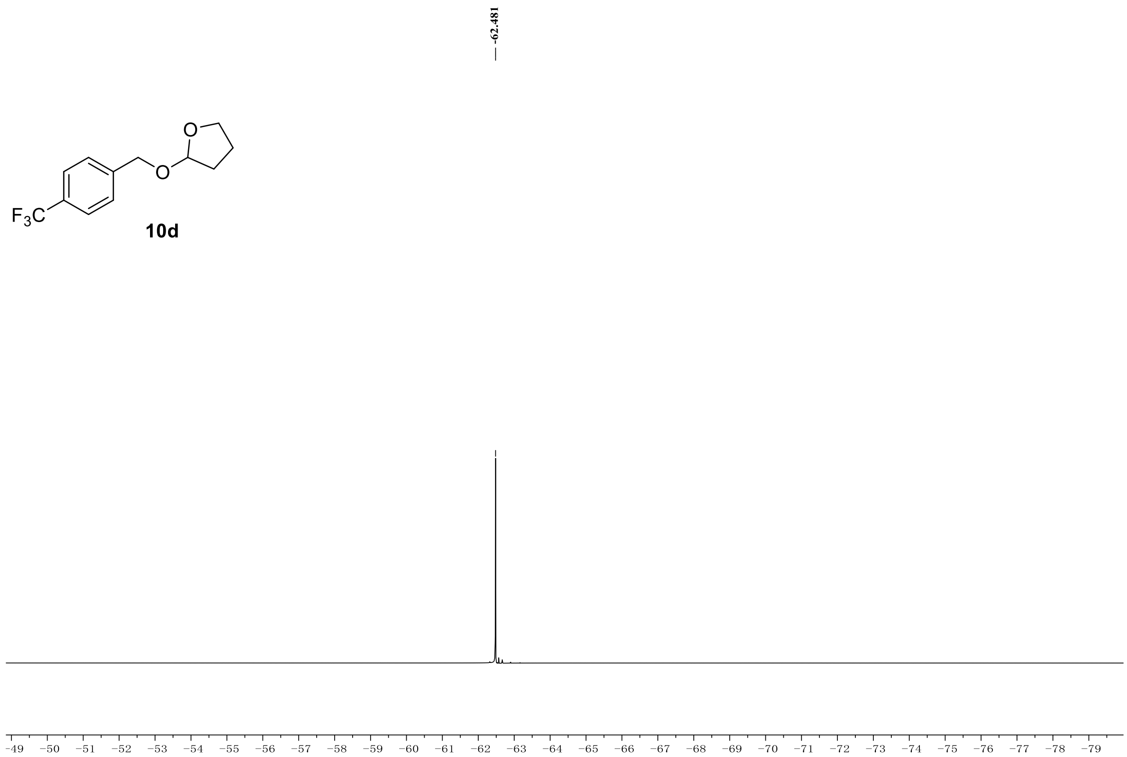

^{19}F NMR was recorded on Bruker 471 MHz; Solvent: $CDCl_3$

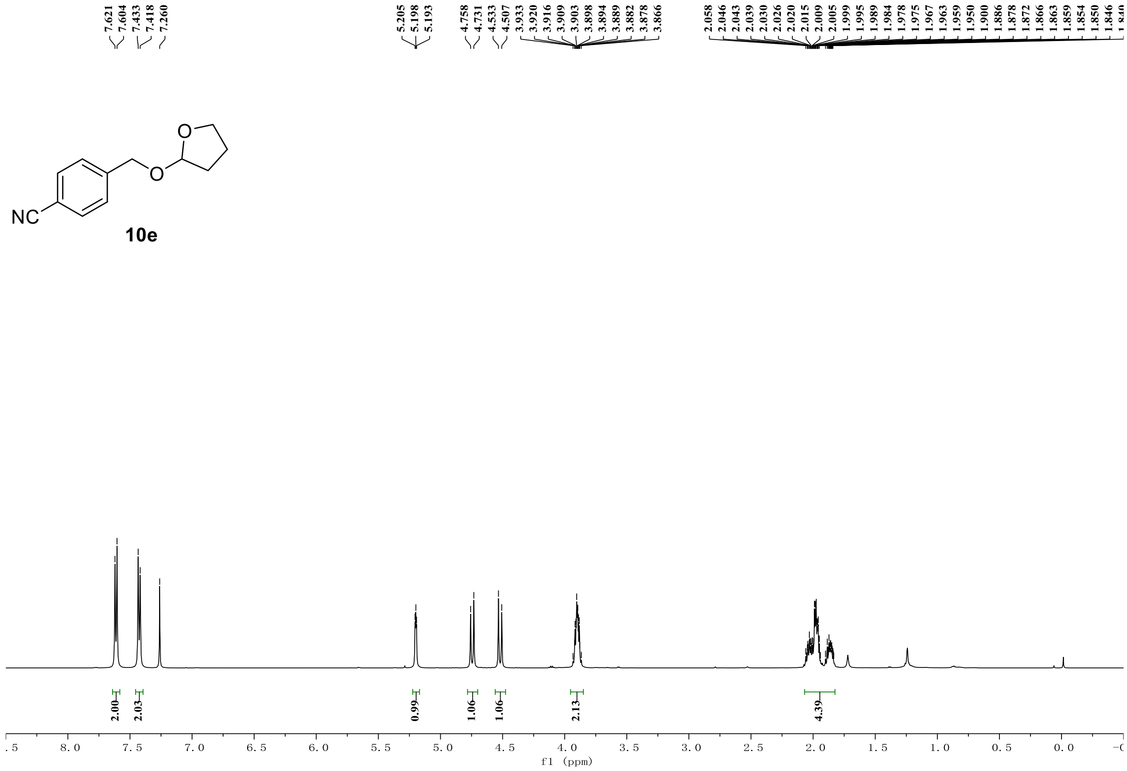

^{1}H NMR was recorded on Bruker 500 MHz; Solvent: $CDCl_3$

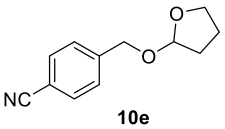

10e

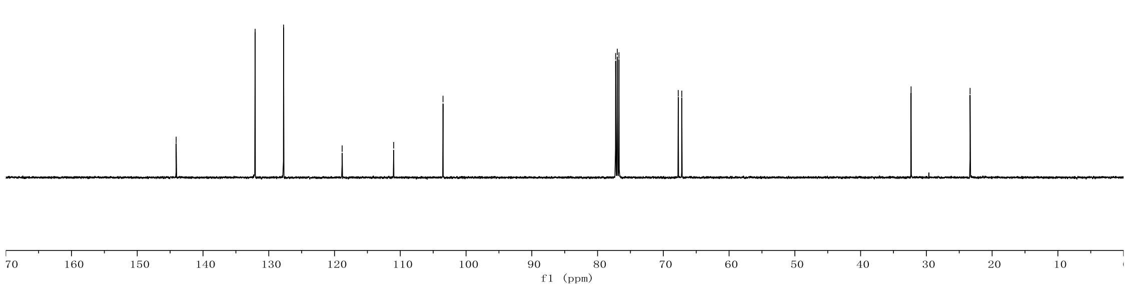

^{13}C NMR was recorded on Bruker 126 MHz; Solvent: $CDCl_3$

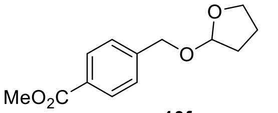

10f

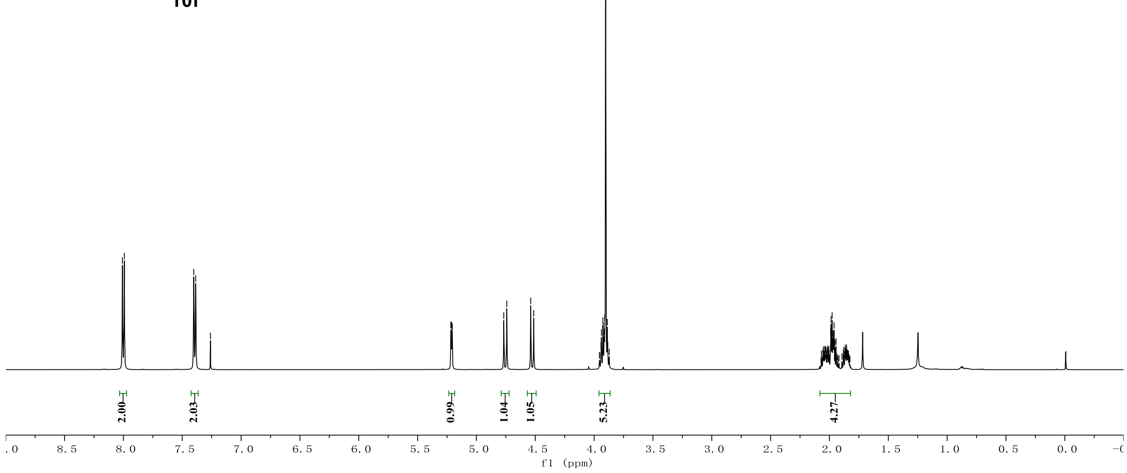

^{1}H NMR was recorded on Bruker 500 MHz; Solvent: $CDCl_3$

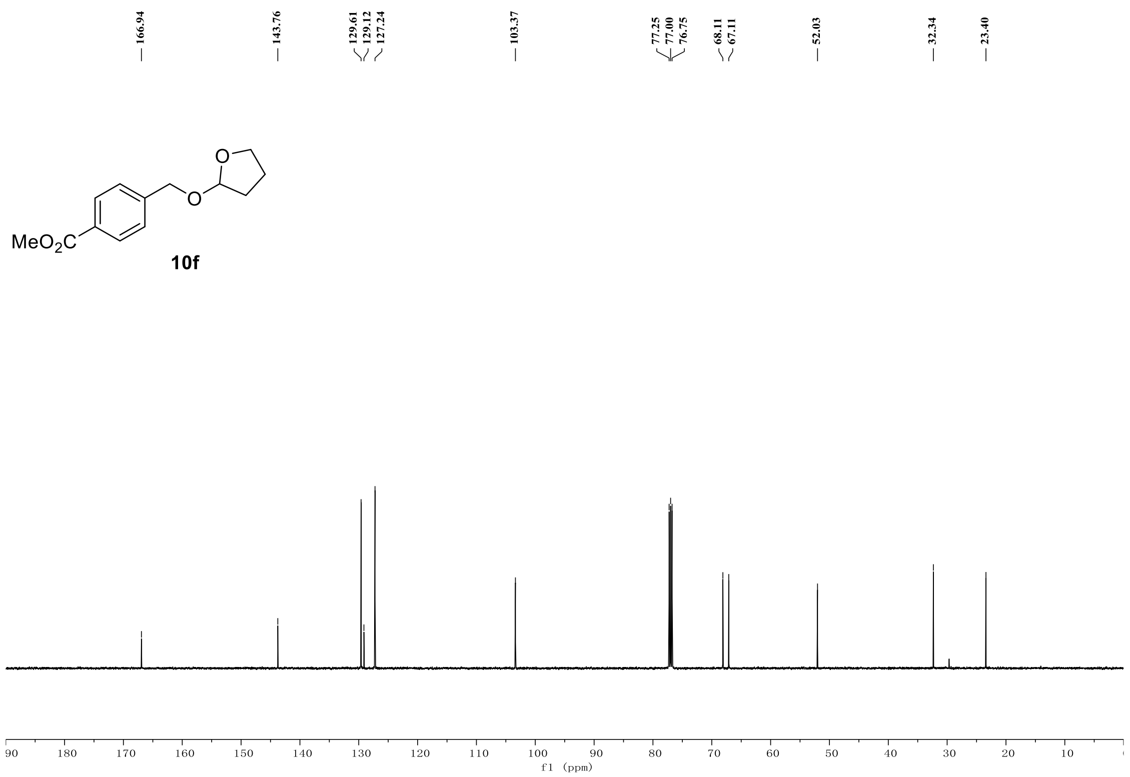

^{13}C NMR was recorded on Bruker 126 MHz; Solvent: $CDCl_3$

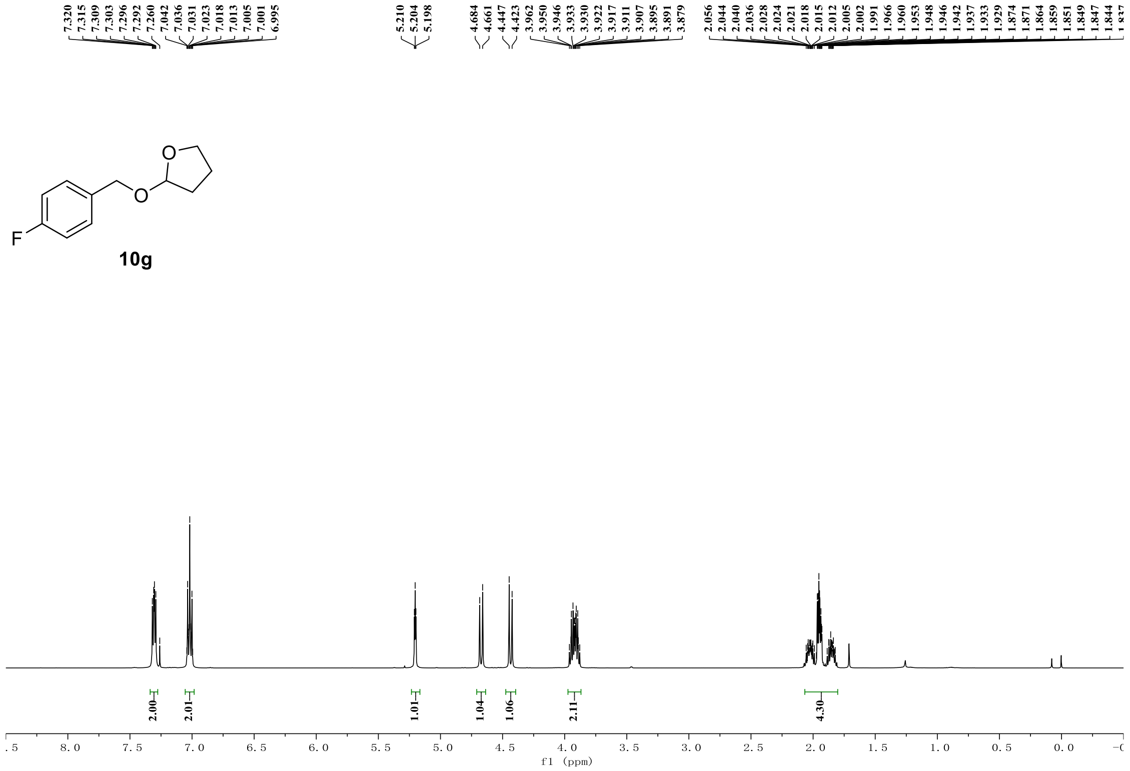

^{1}H NMR was recorded on Bruker 500 MHz; Solvent: $CDCl_3$

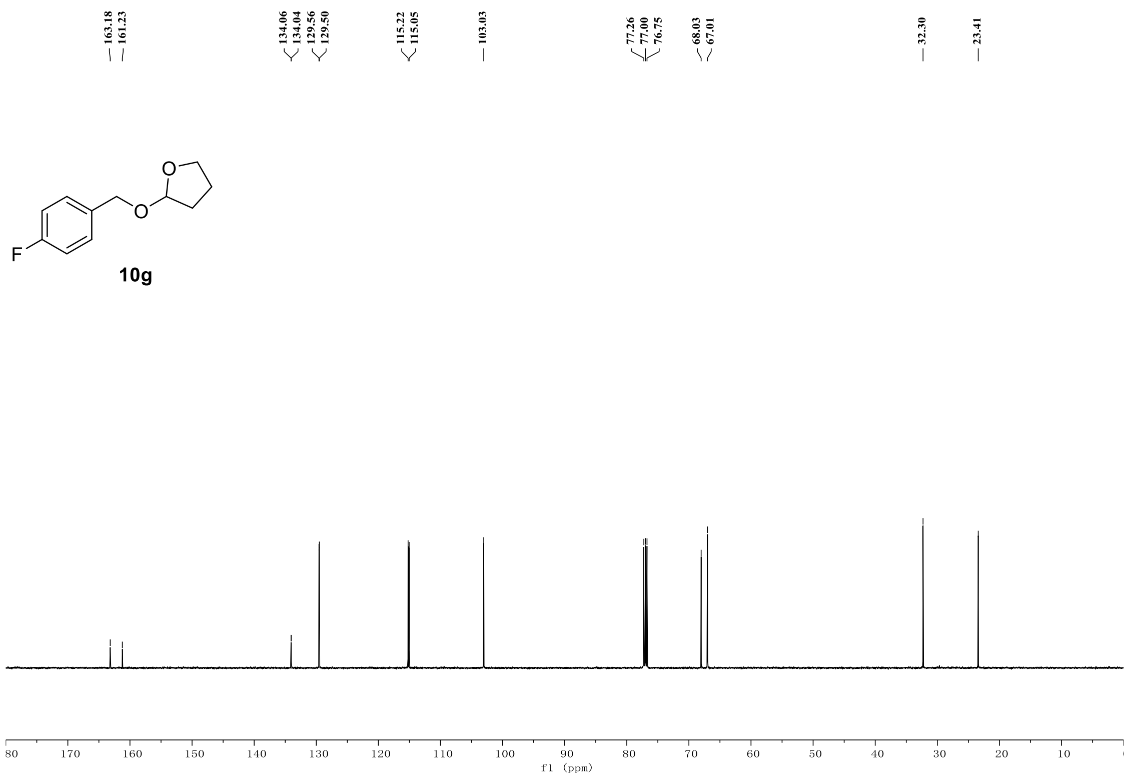

^{13}C NMR was recorded on Bruker 126 MHz; Solvent: $CDCl_3$

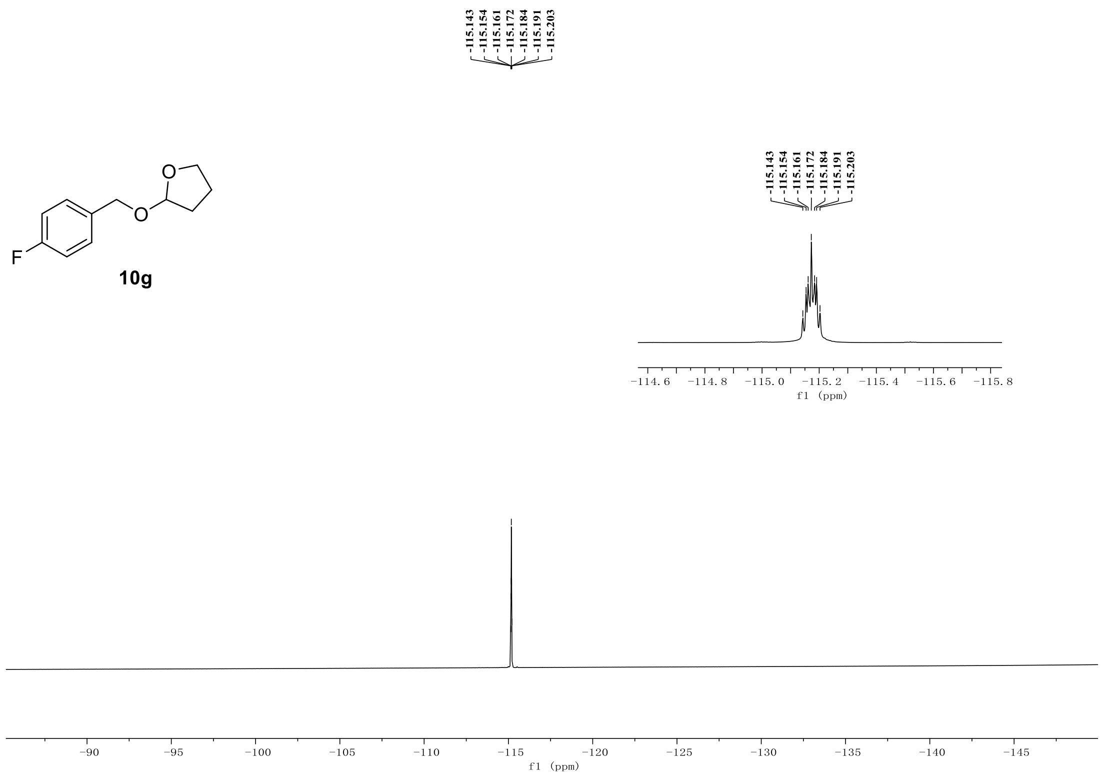

^{19}F NMR was recorded on Bruker 471 MHz; Solvent: $CDCl_3$

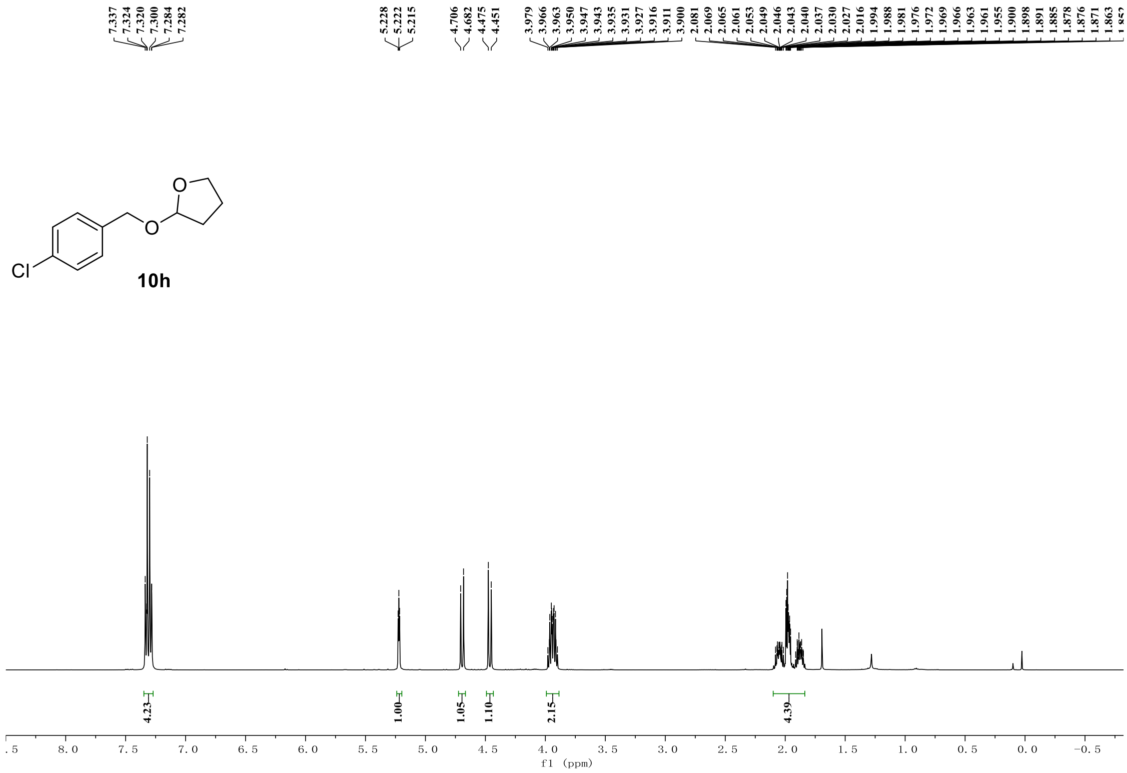

^{1}H NMR was recorded on Bruker 500 MHz; Solvent: $CDCl_3$

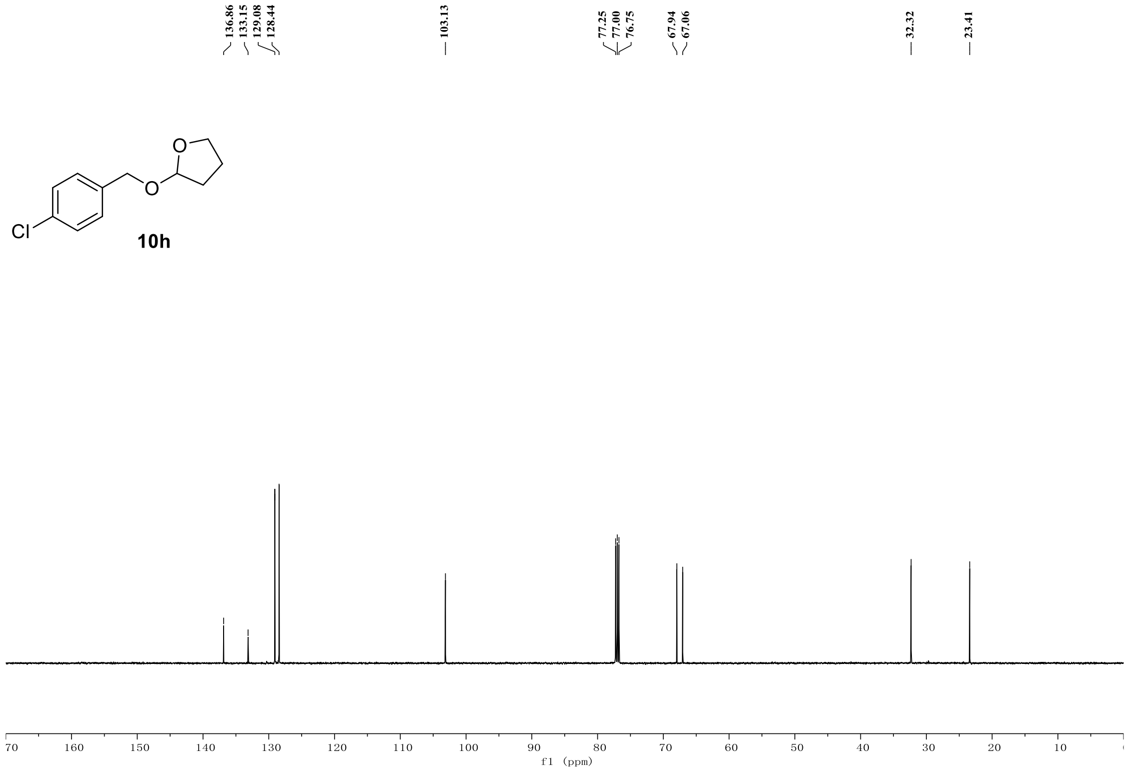

^{13}C NMR was recorded on Bruker 126 MHz; Solvent: $CDCl_3$

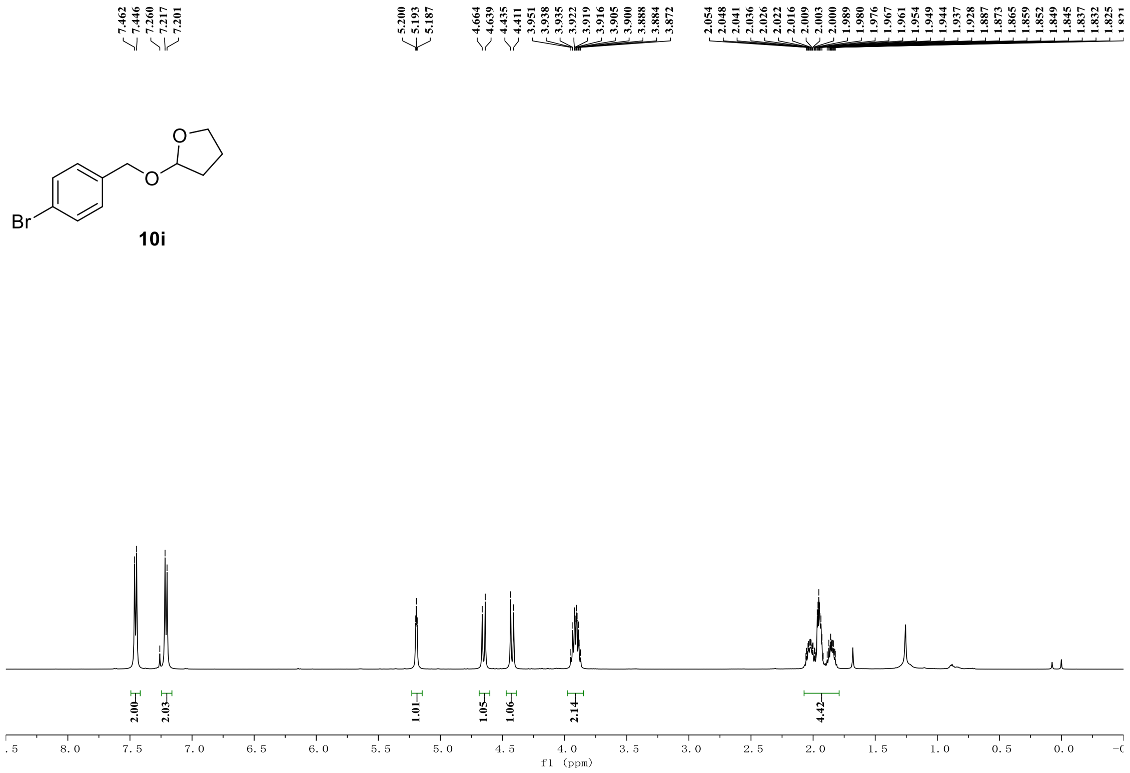

^{1}H NMR was recorded on Bruker 500 MHz; Solvent: $CDCl_3$

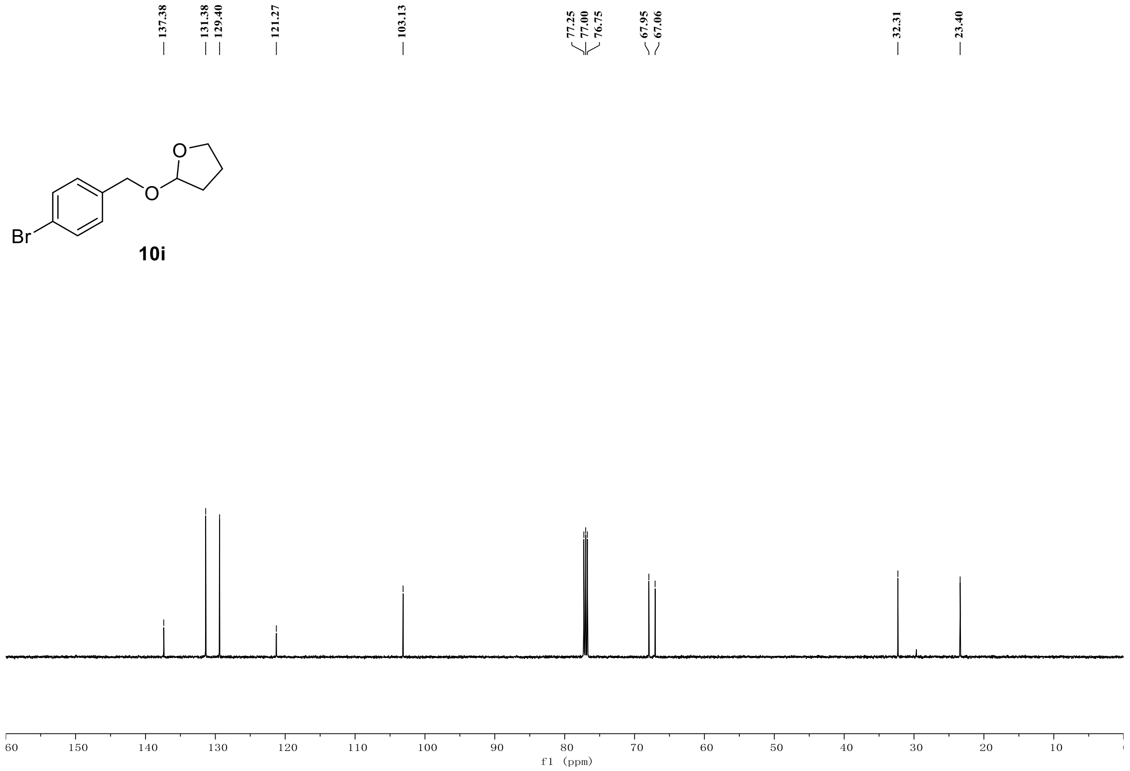

^{13}C NMR was recorded on Bruker 126 MHz; Solvent: $CDCl_3$

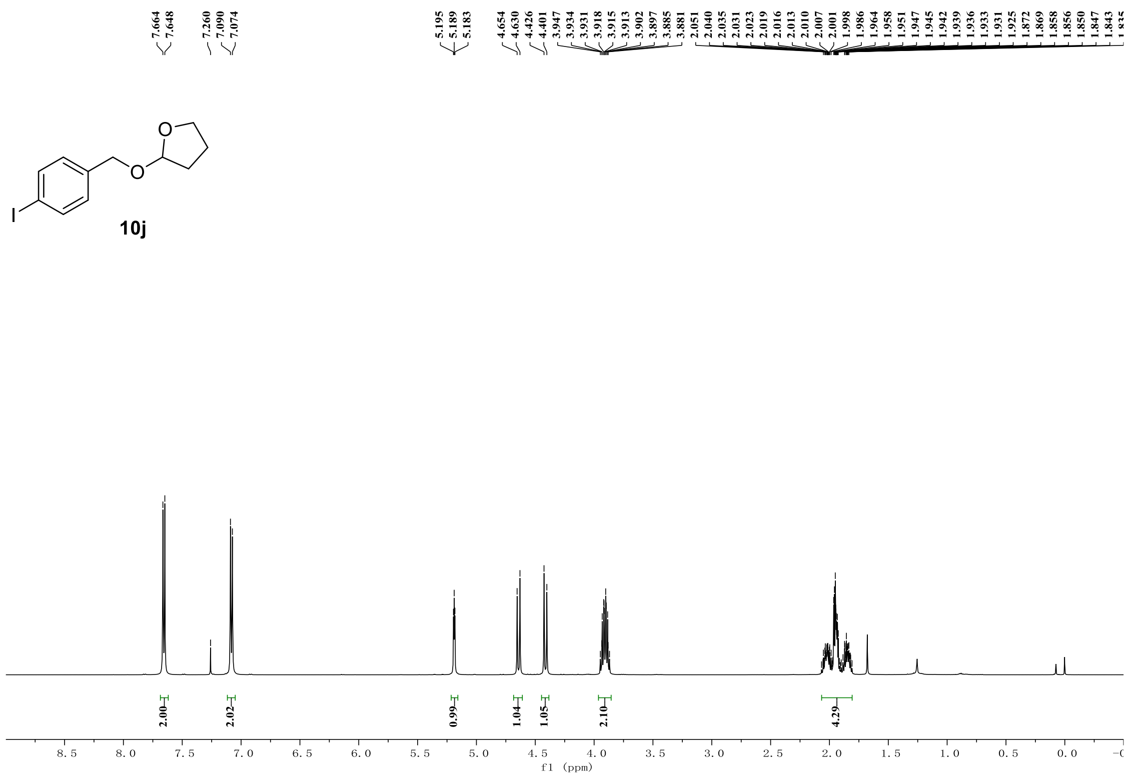

^{1}H NMR was recorded on Bruker 500 MHz; Solvent: $CDCl_3$

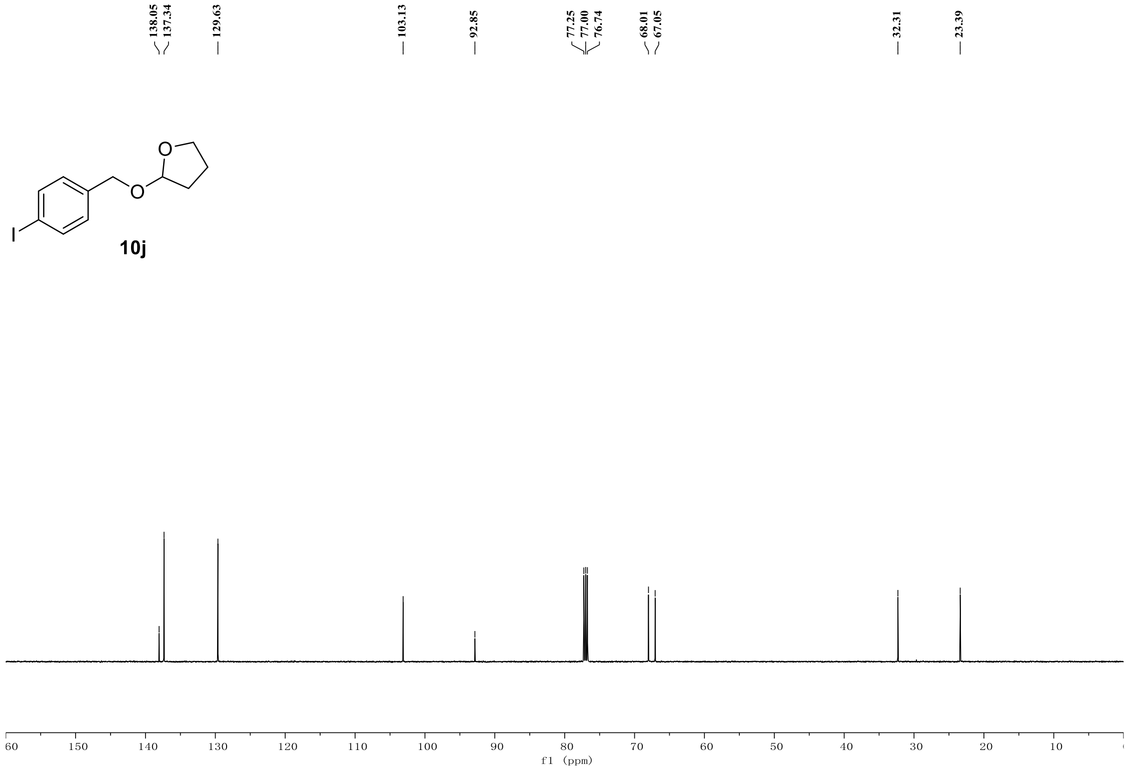

^{13}C NMR was recorded on Bruker 126 MHz; Solvent: $CDCl_3$

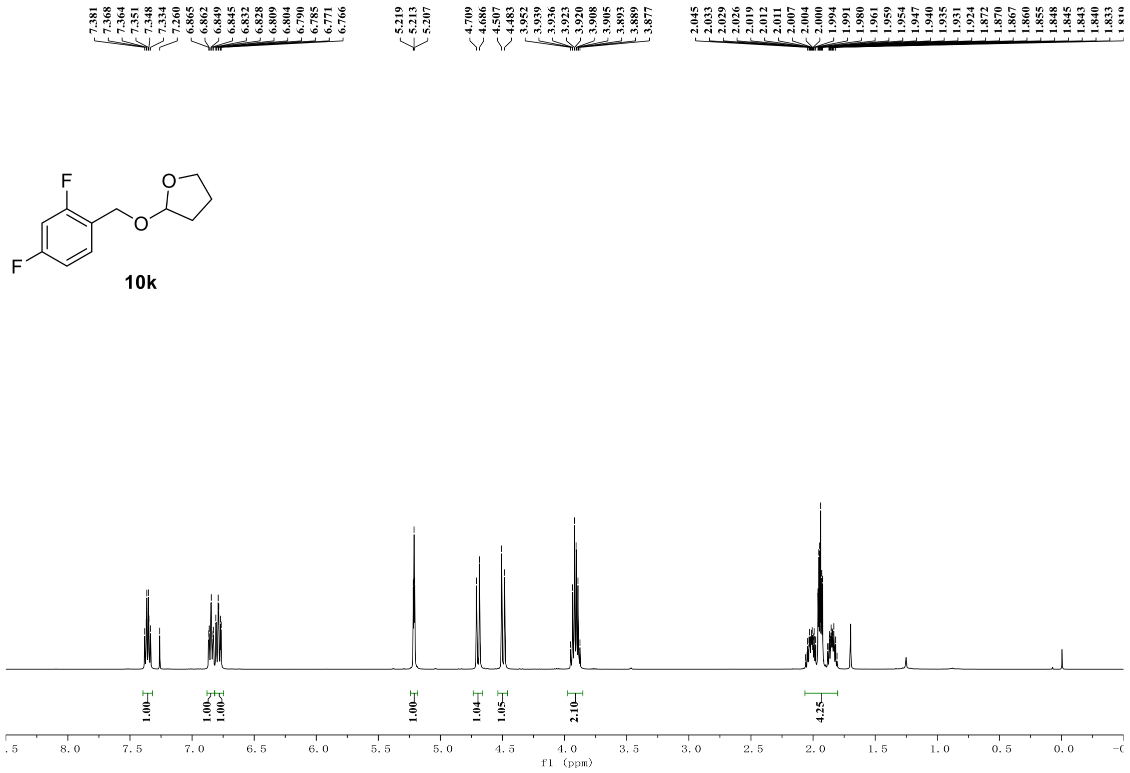

^{1}H NMR was recorded on Bruker 500 MHz; Solvent: $CDCl_3$

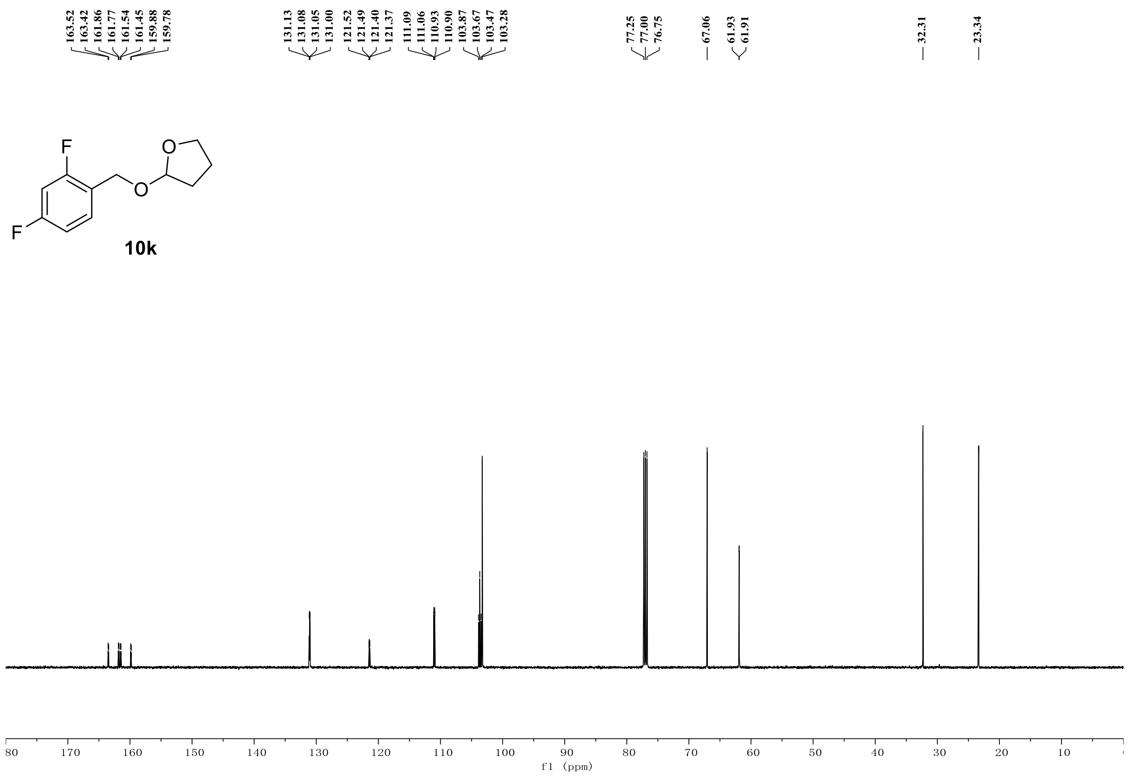

^{13}C NMR was recorded on Bruker 126 MHz; Solvent: $CDCl_3$

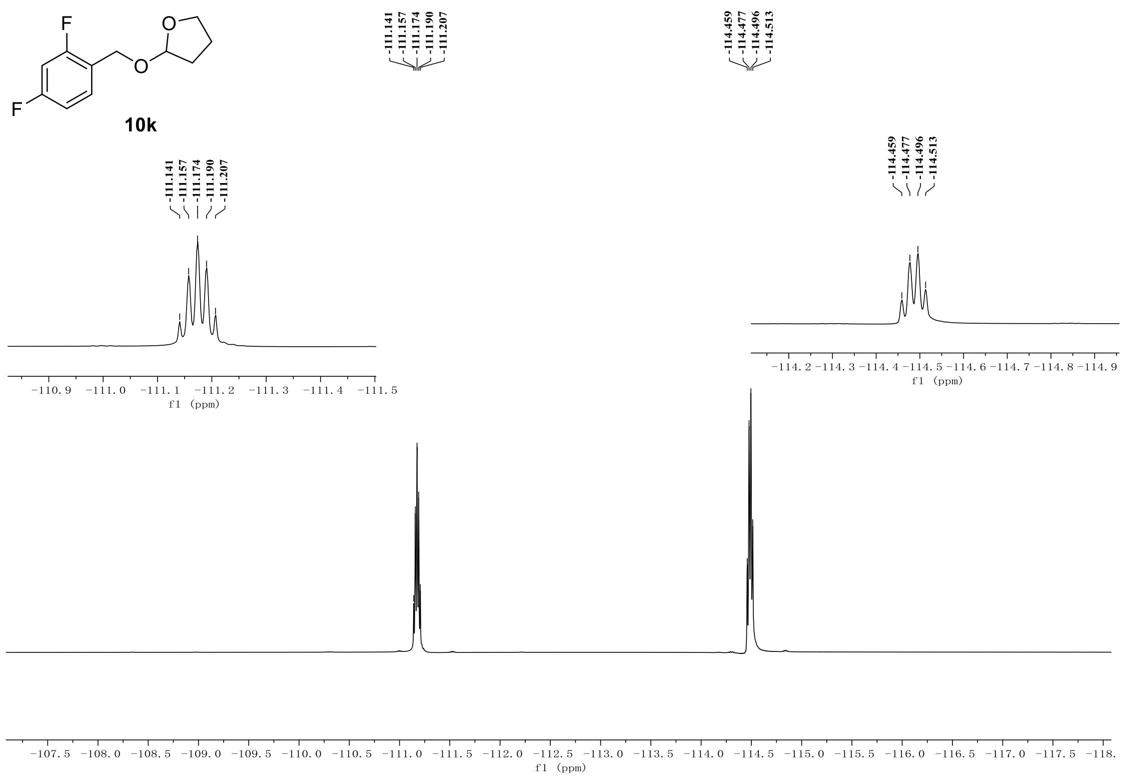

^{19}F NMR was recorded on Bruker 471 MHz; Solvent: $CDCl_3$

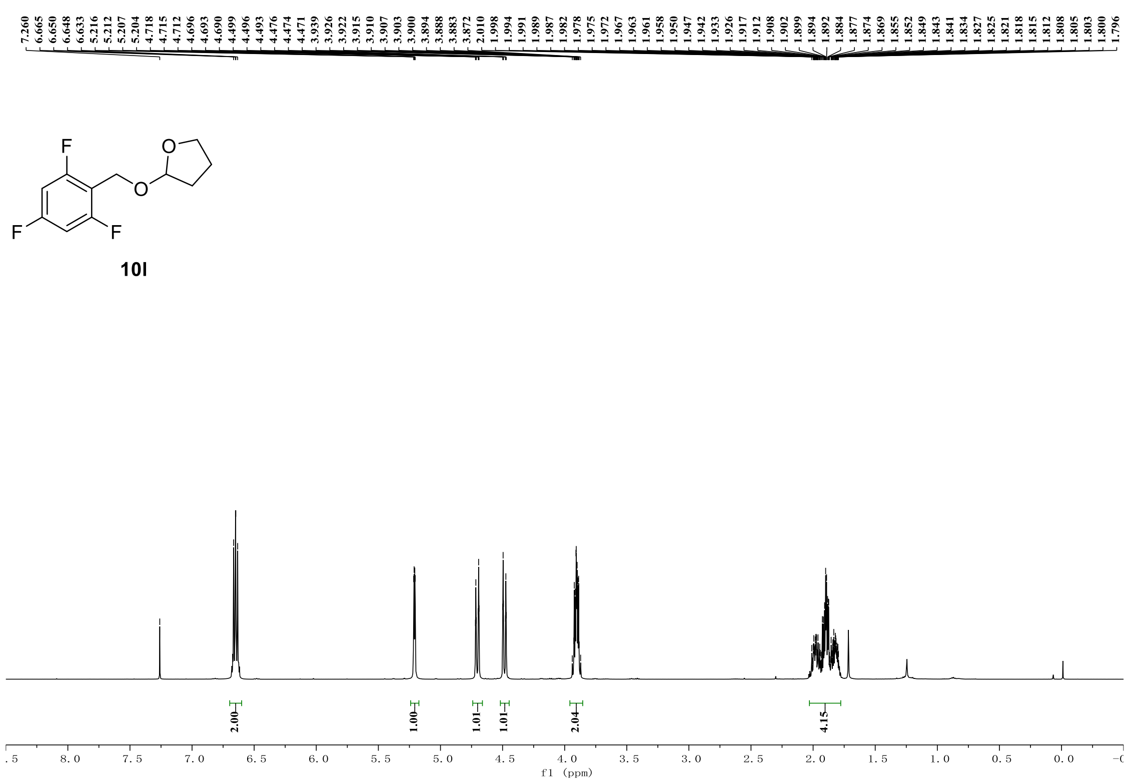

^{1}H NMR was recorded on Bruker 500 MHz; Solvent: $CDCl_3$

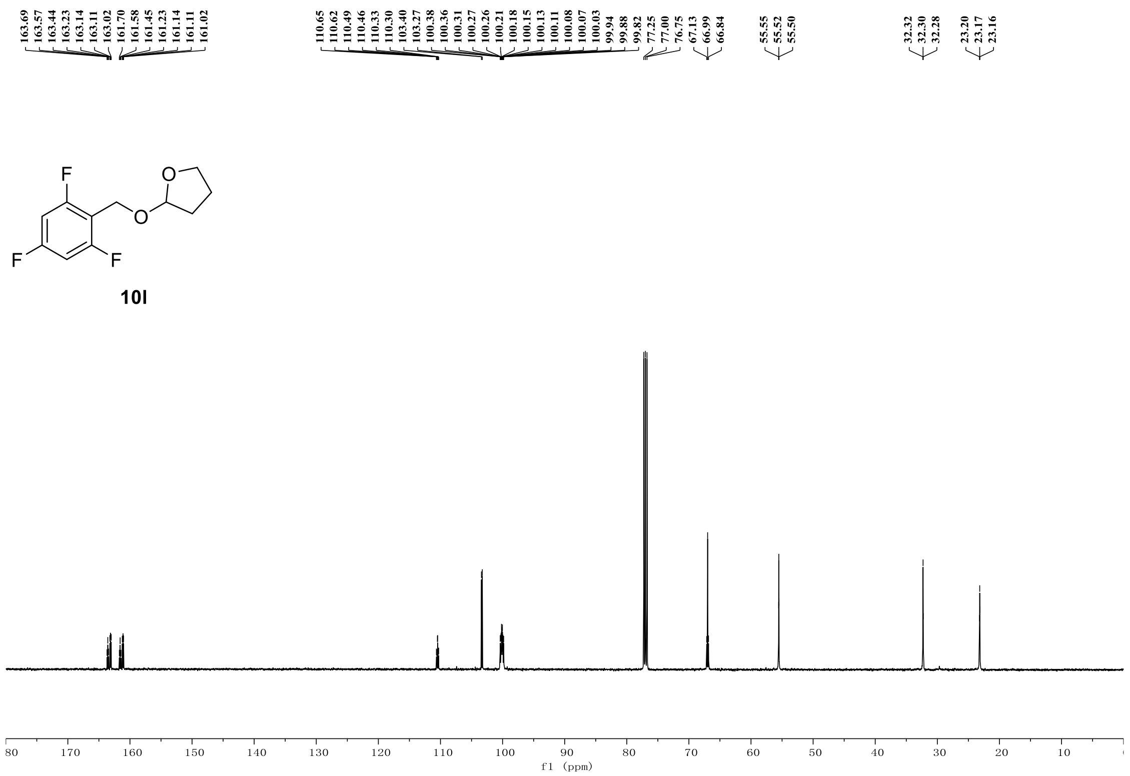

^{13}C NMR was recorded on Bruker 126 MHz; Solvent: $CDCl_3$

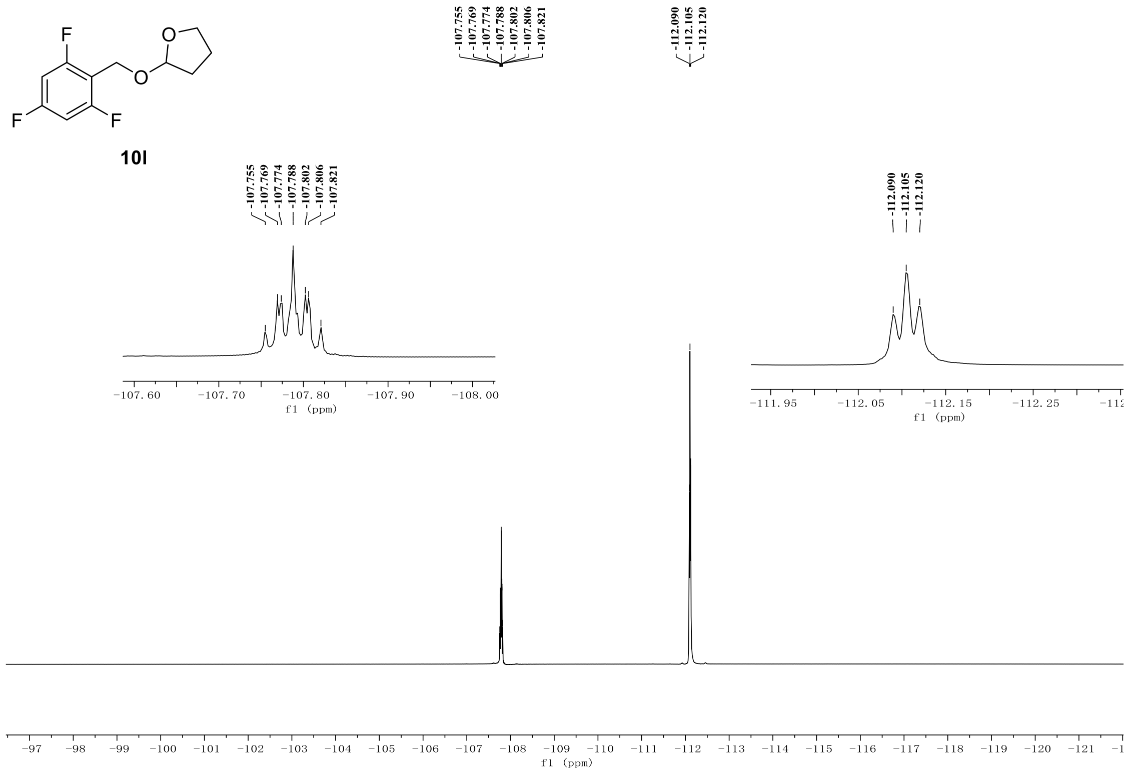

^{19}F NMR was recorded on Bruker 471 MHz; Solvent: $CDCl_3$

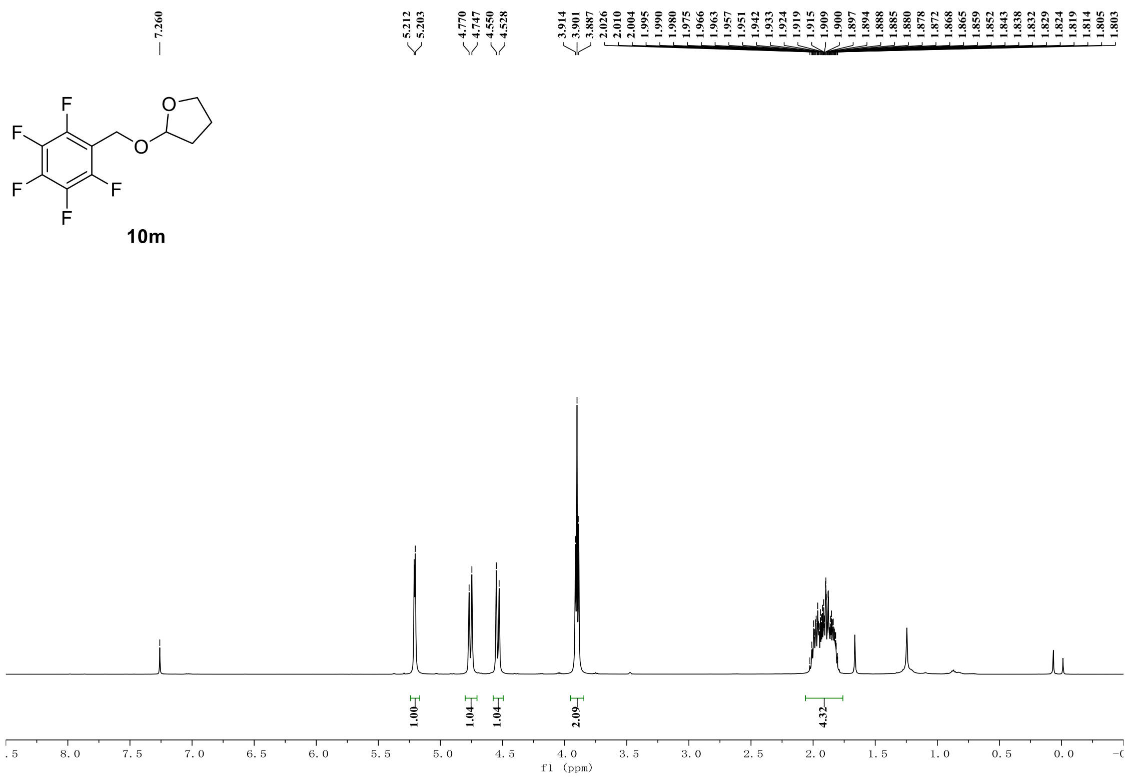

10m

^{1}H NMR was recorded on Bruker 500 MHz; Solvent: $CDCl_3$

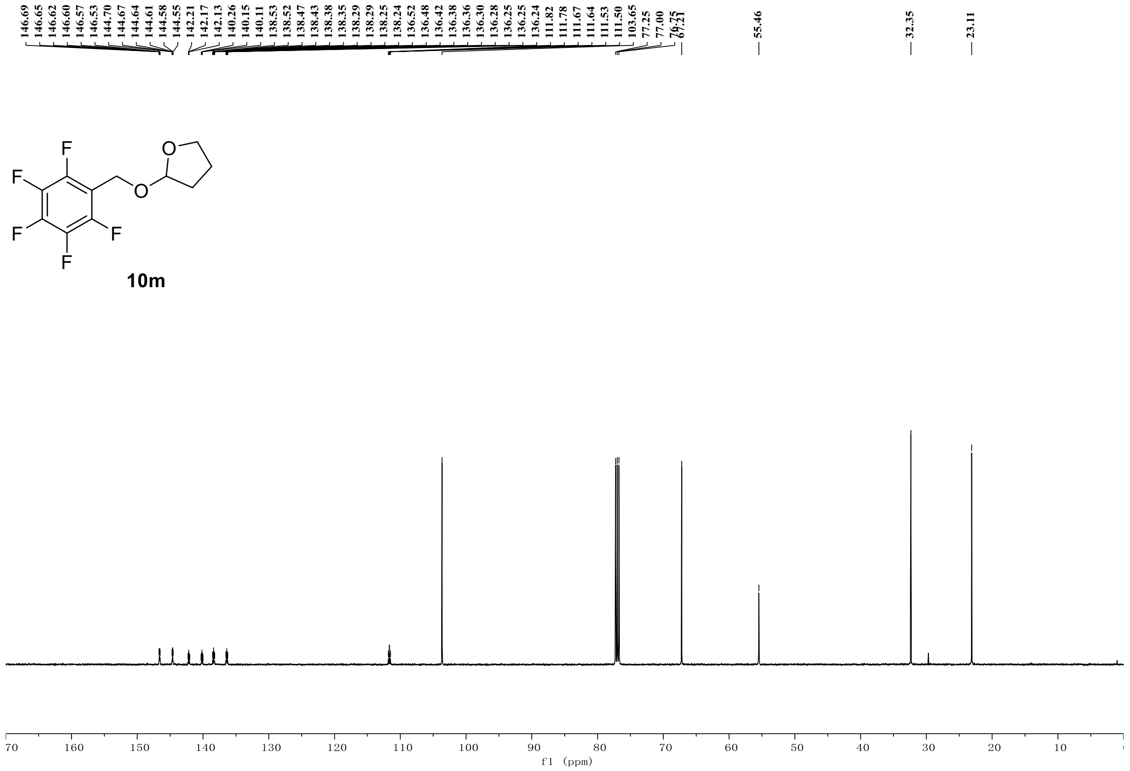

10m

^{13}C NMR was recorded on Bruker 126 MHz; Solvent: $CDCl_3$

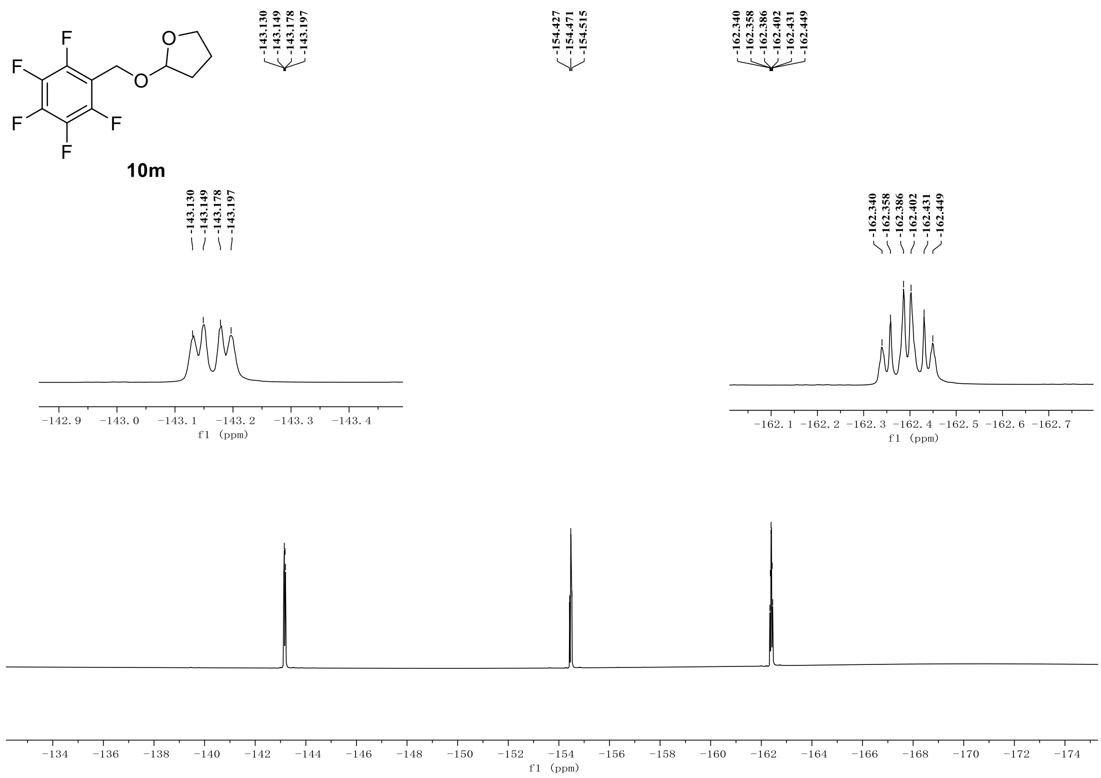

^{19}F NMR was recorded on Bruker 471 MHz; Solvent: $CDCl_3$

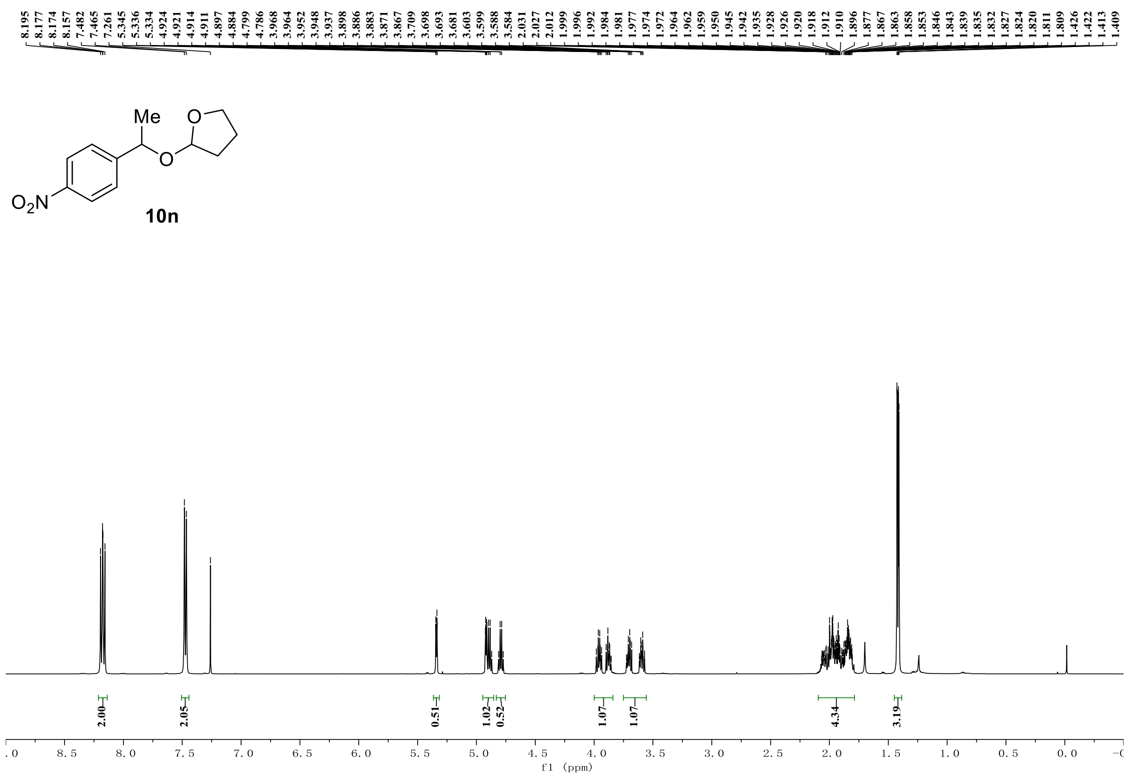

^{1}H NMR was recorded on Bruker 500 MHz; Solvent: $CDCl_3$

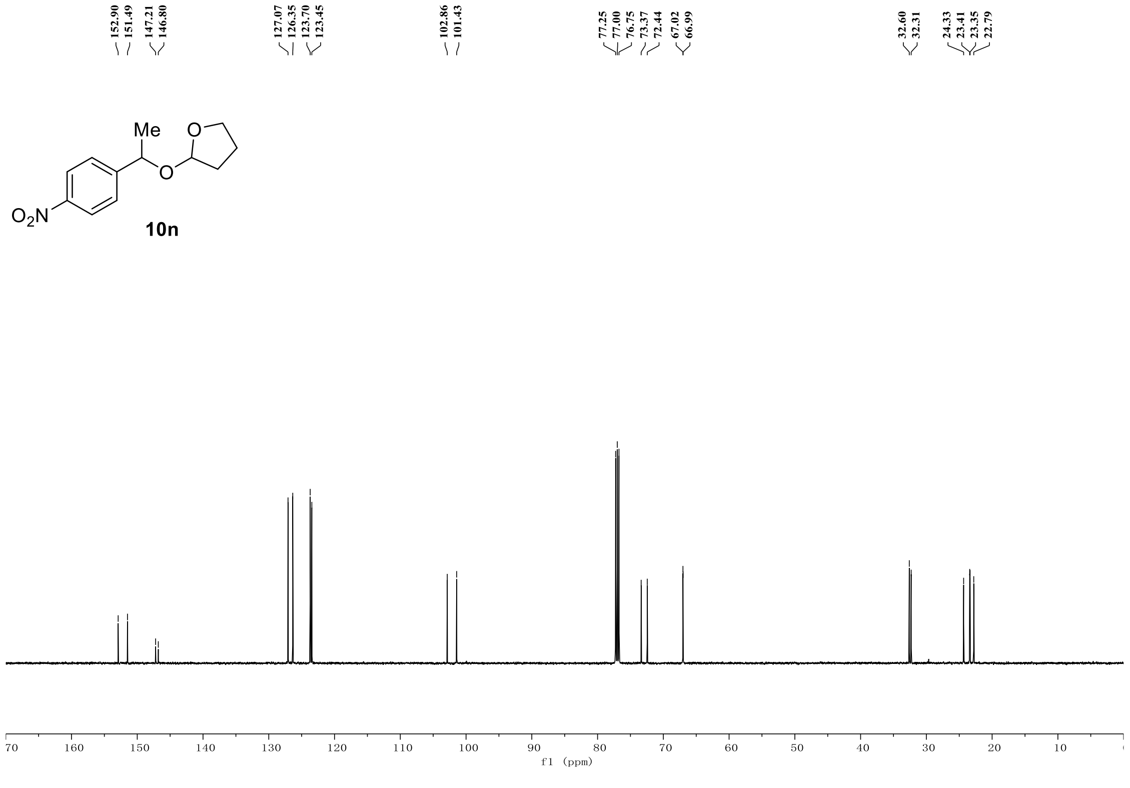

^{13}C NMR was recorded on Bruker 126 MHz; Solvent: $CDCl_3$

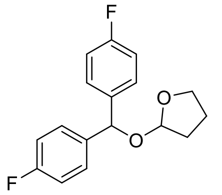

10o

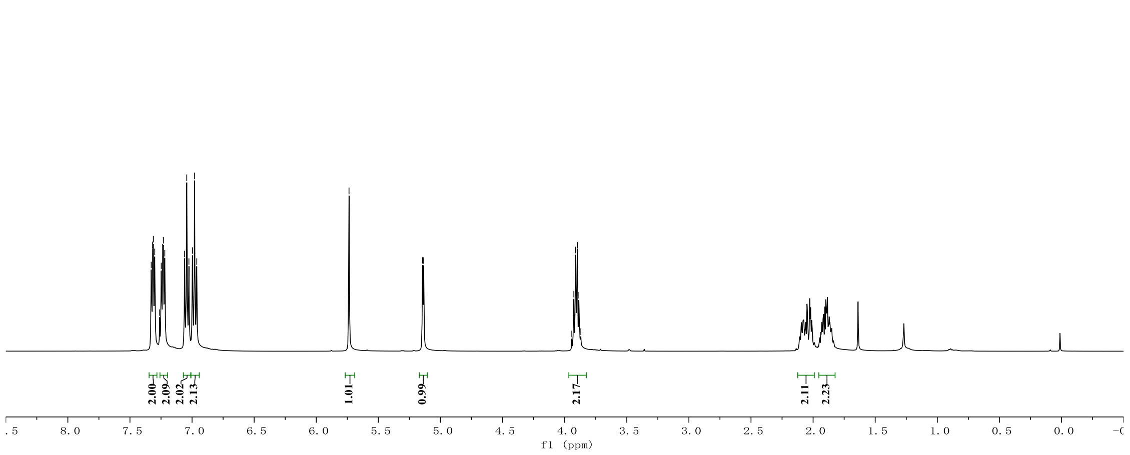

^{1}H NMR was recorded on Bruker 500 MHz; Solvent: $CDCl_3$

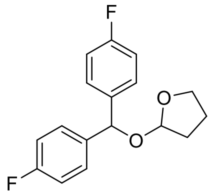

10o

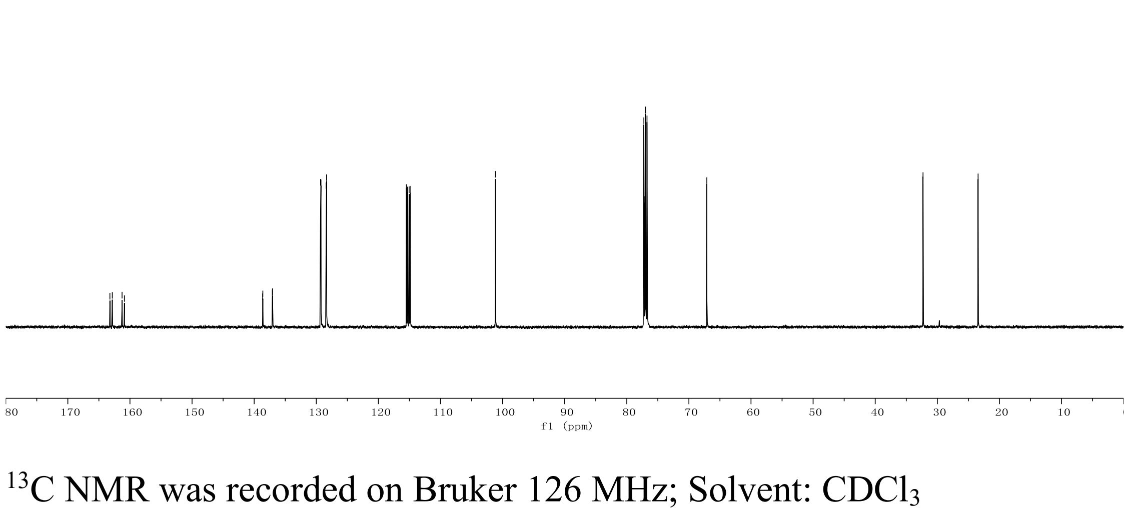

^{13}C NMR was recorded on Bruker 126 MHz; Solvent: $CDCl_3$

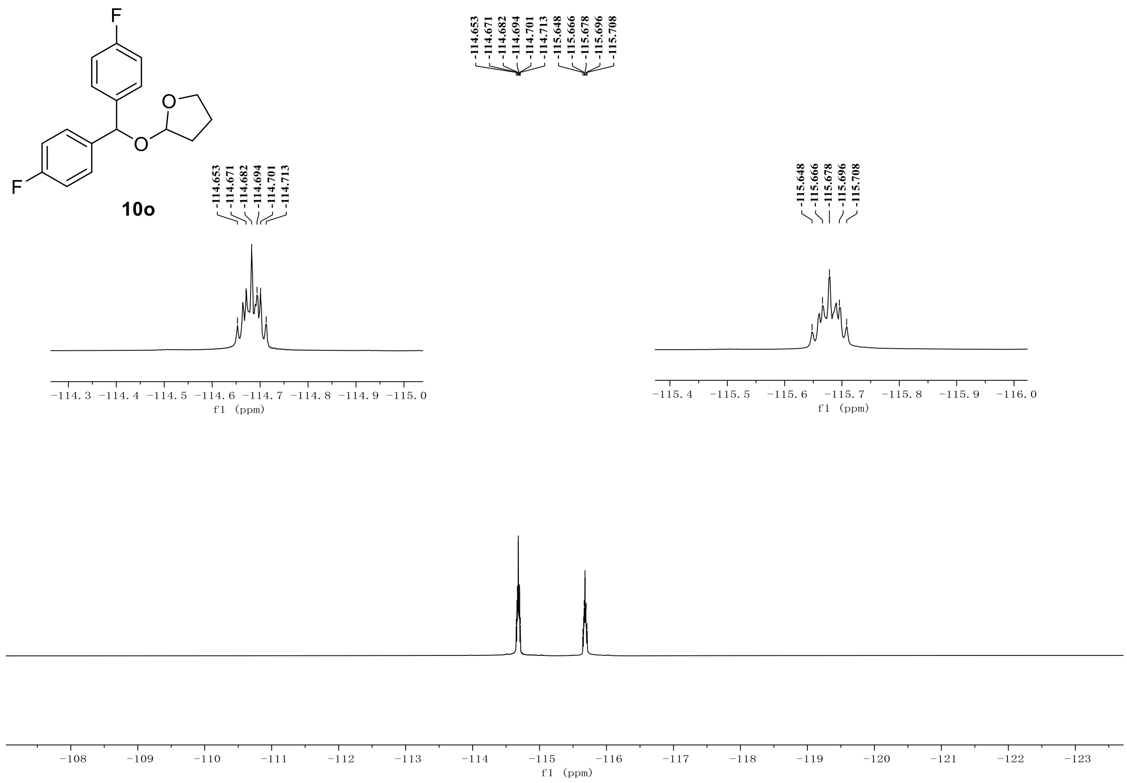

^{19}F NMR was recorded on Bruker 471 MHz; Solvent: $CDCl_3$

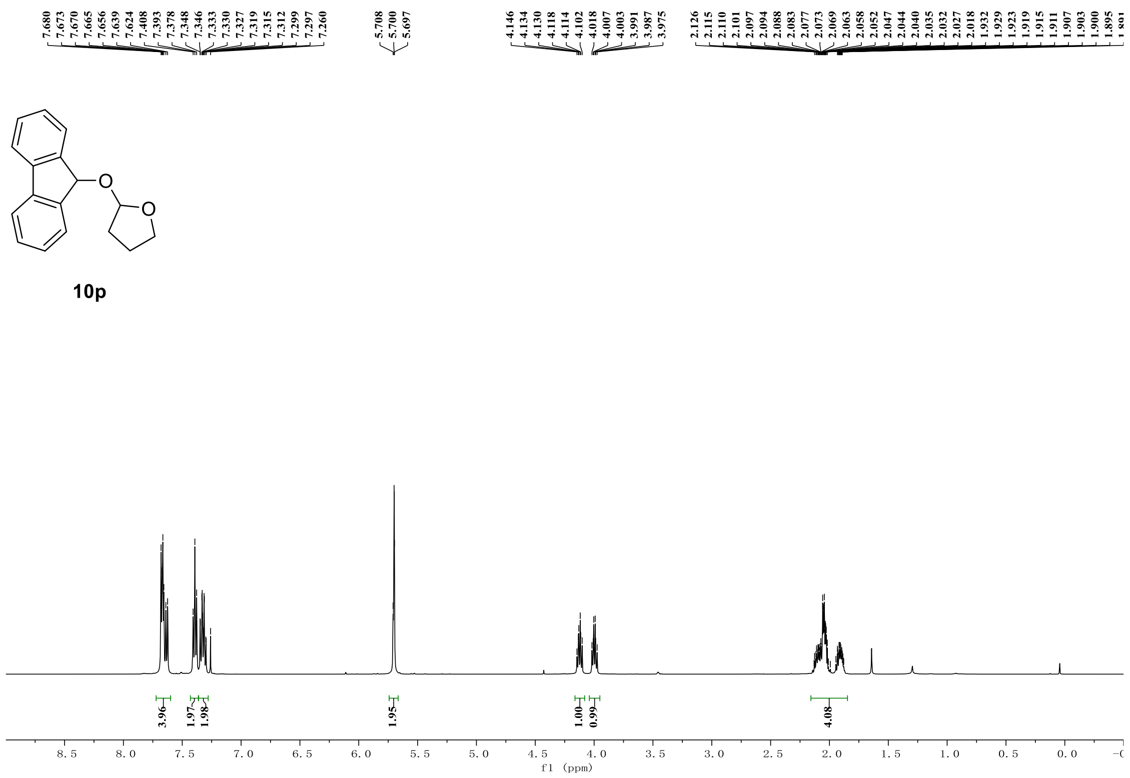

^{1}H NMR was recorded on Bruker 500 MHz; Solvent: $CDCl_3$

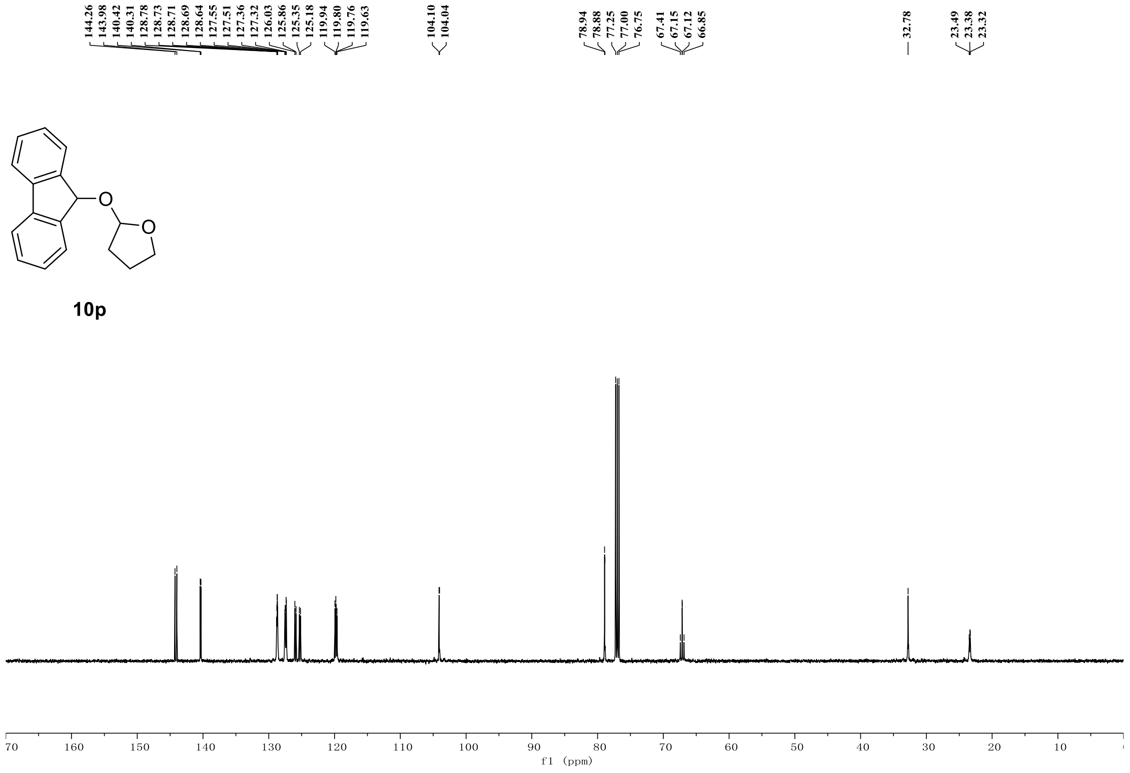

^{13}C NMR was recorded on Bruker 126 MHz; Solvent: $CDCl_3$

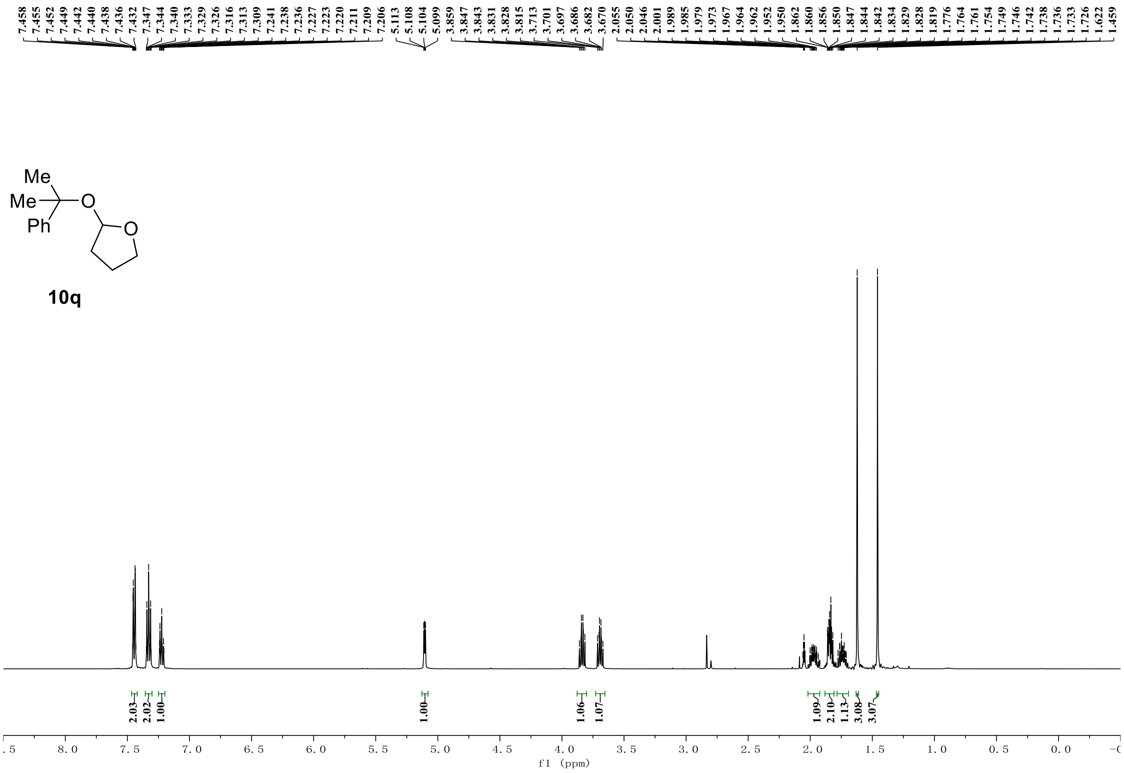

10q

^{1}H NMR was recorded on Bruker 500 MHz; Solvent: Acetone-d_6

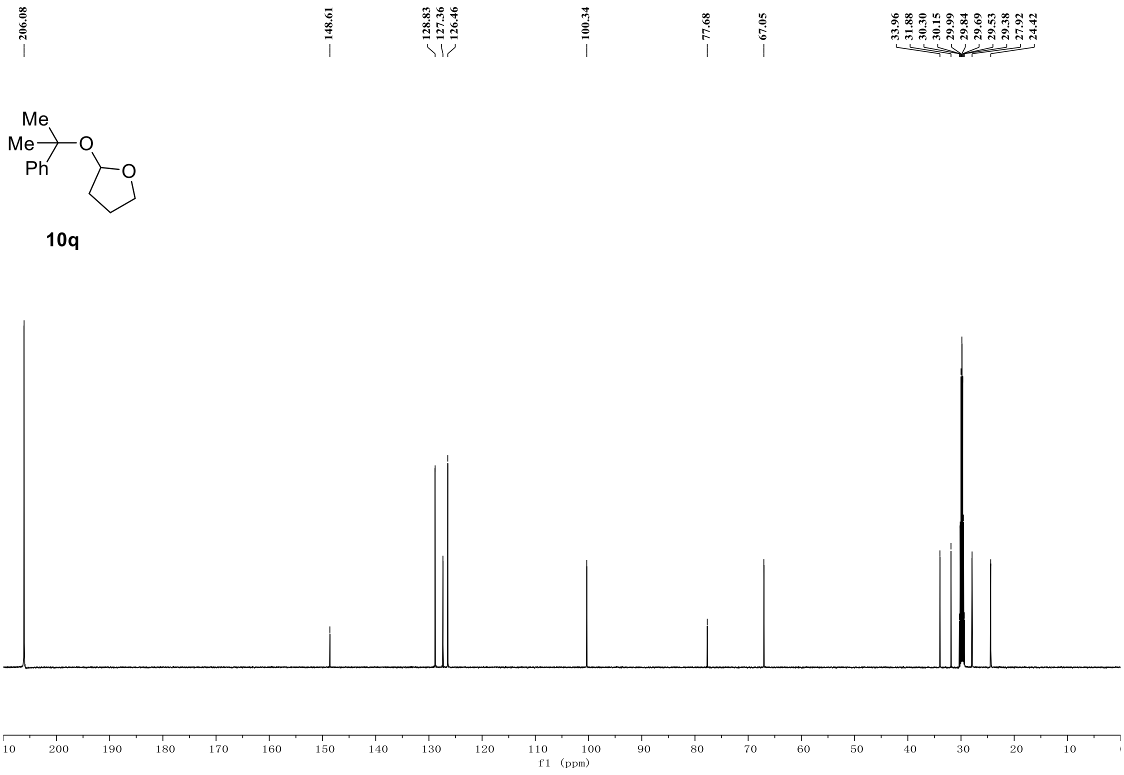

10q

^{13}C NMR was recorded on Bruker 126 MHz; Solvent: Acetone-d_6

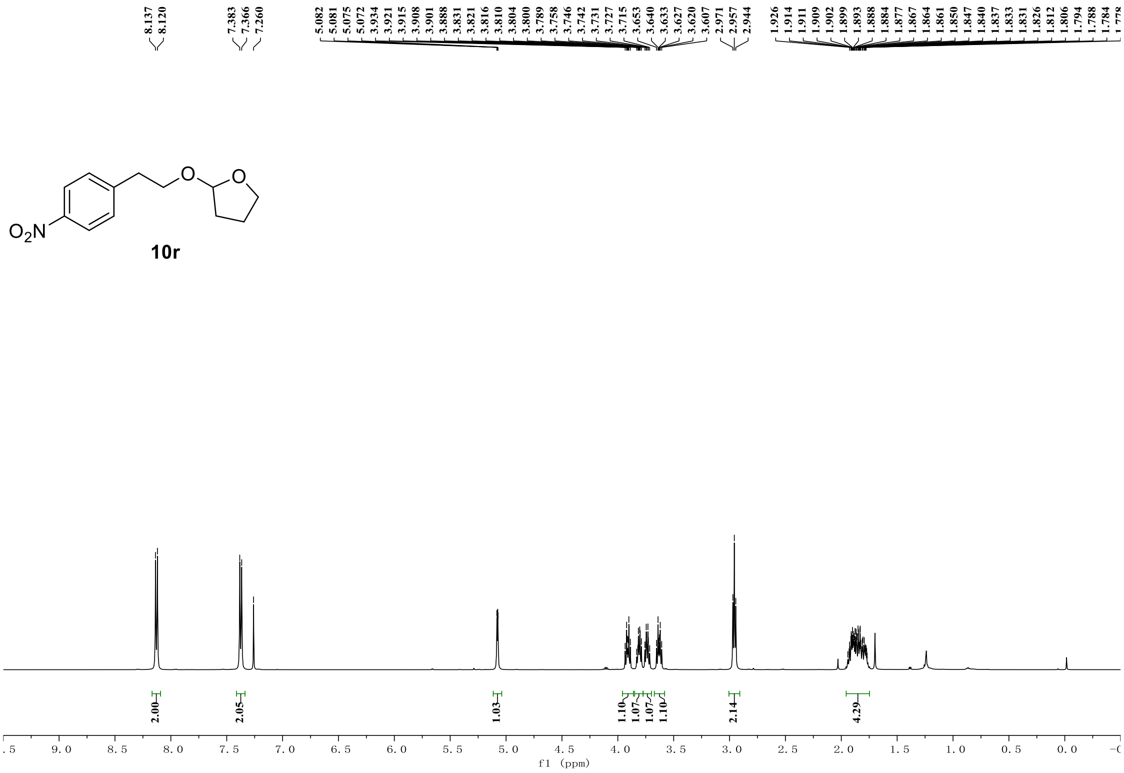

^{1}H NMR was recorded on Bruker 500 MHz; Solvent: $CDCl_3$

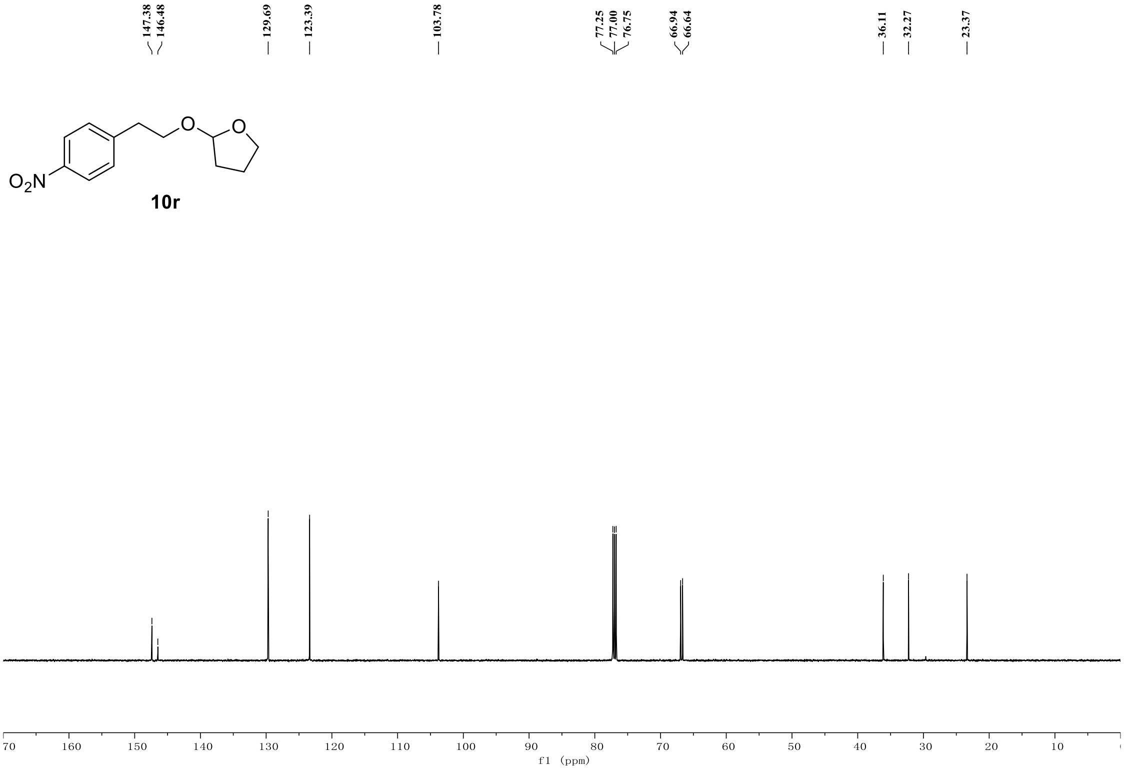

^{13}C NMR was recorded on Bruker 126 MHz; Solvent: $CDCl_3$

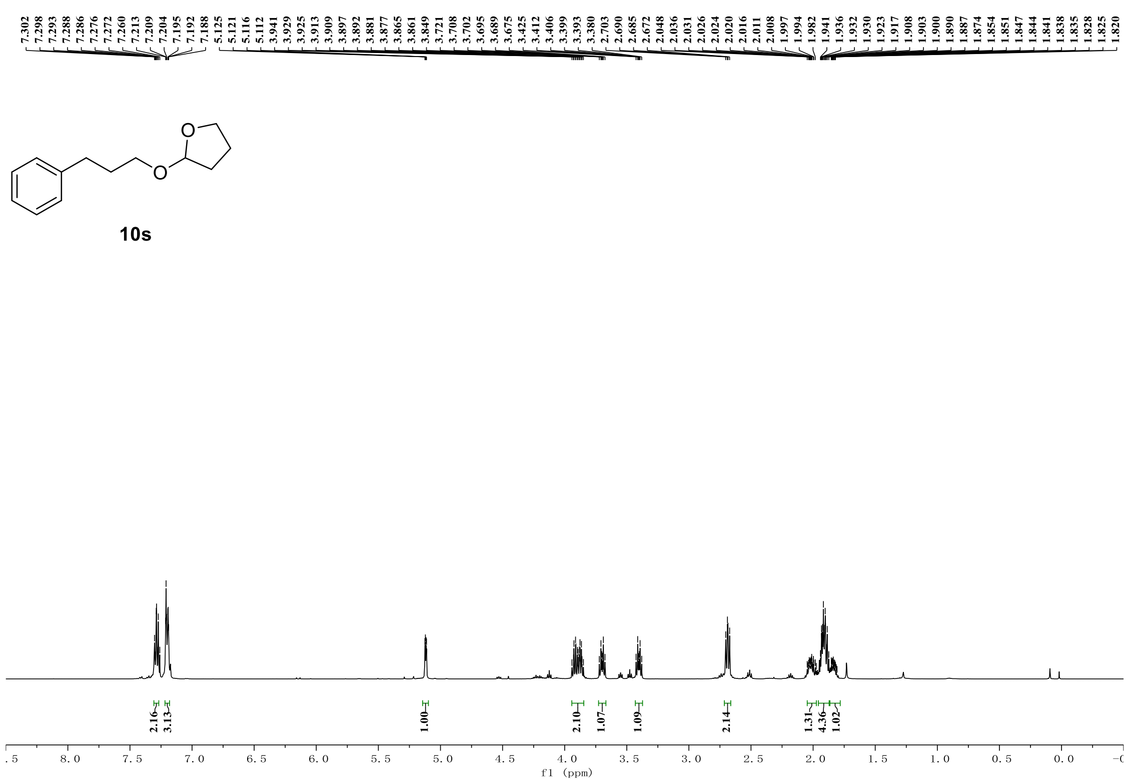

^{1}H NMR was recorded on Bruker 500 MHz; Solvent: $CDCl_3$

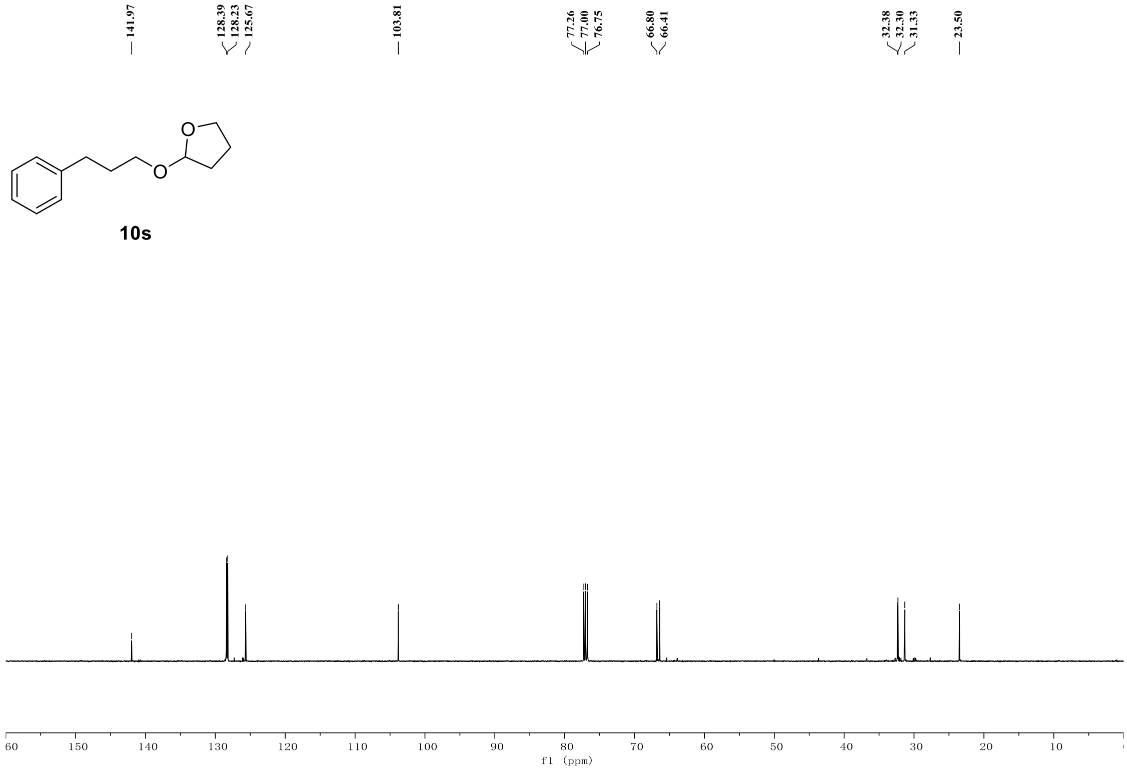

^{13}C NMR was recorded on Bruker 126 MHz; Solvent: $CDCl_3$

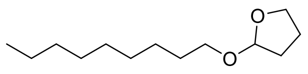

10t

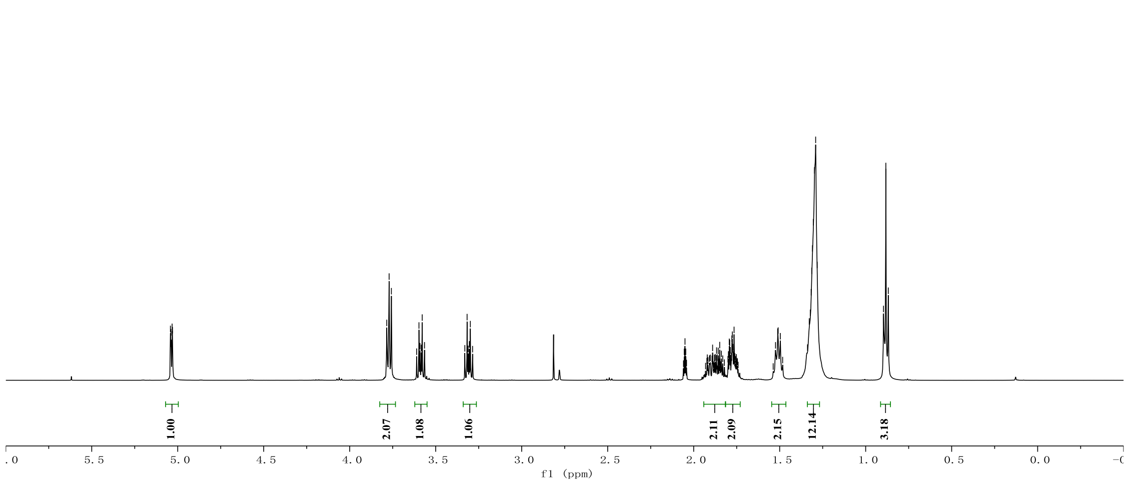

^{1}H NMR was recorded on Bruker 500 MHz; Solvent: Acetone-d_6

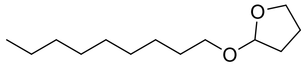

10t

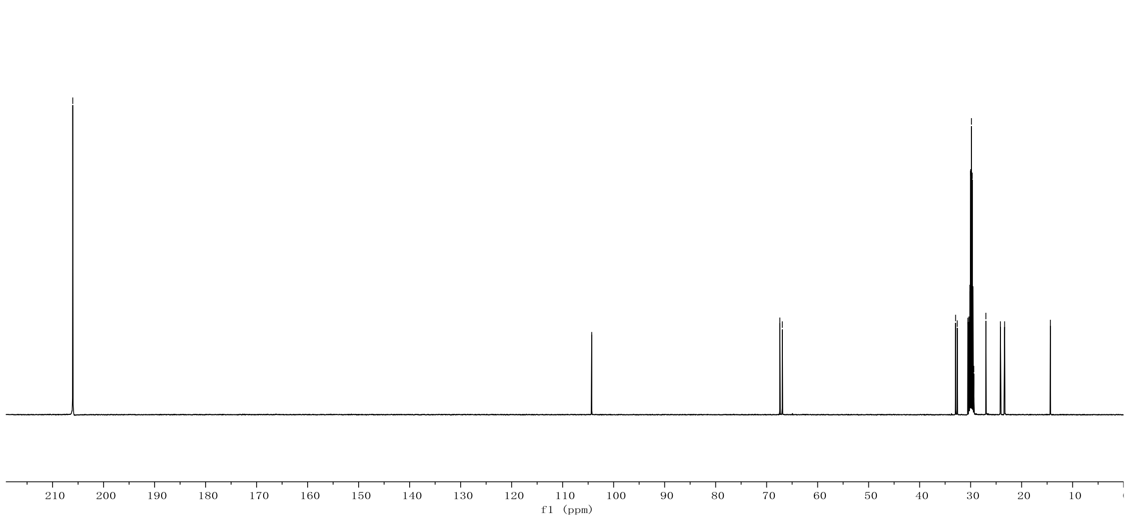

^{13}C NMR was recorded on Bruker 126 MHz; Solvent: Acetone-d_6

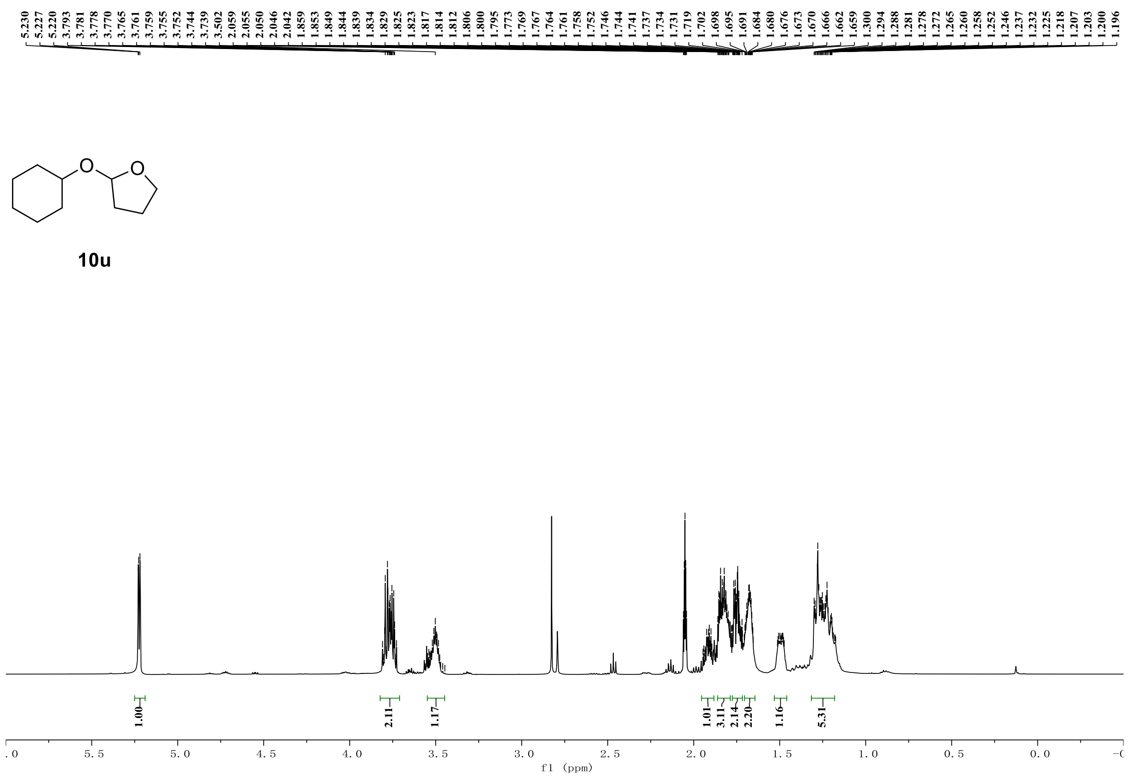

^{1}H NMR was recorded on Bruker 500 MHz; Solvent: Acetone-d_6

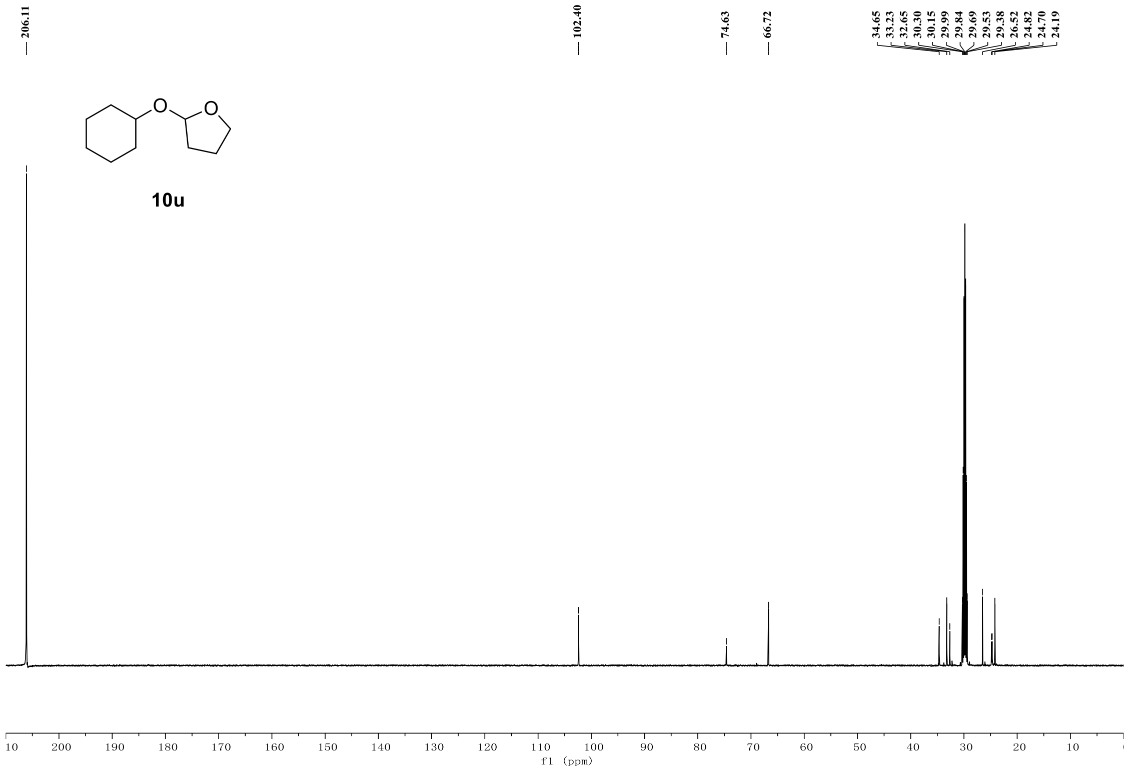

^{13}C NMR was recorded on Bruker 126 MHz; Solvent: Acetone-d_6

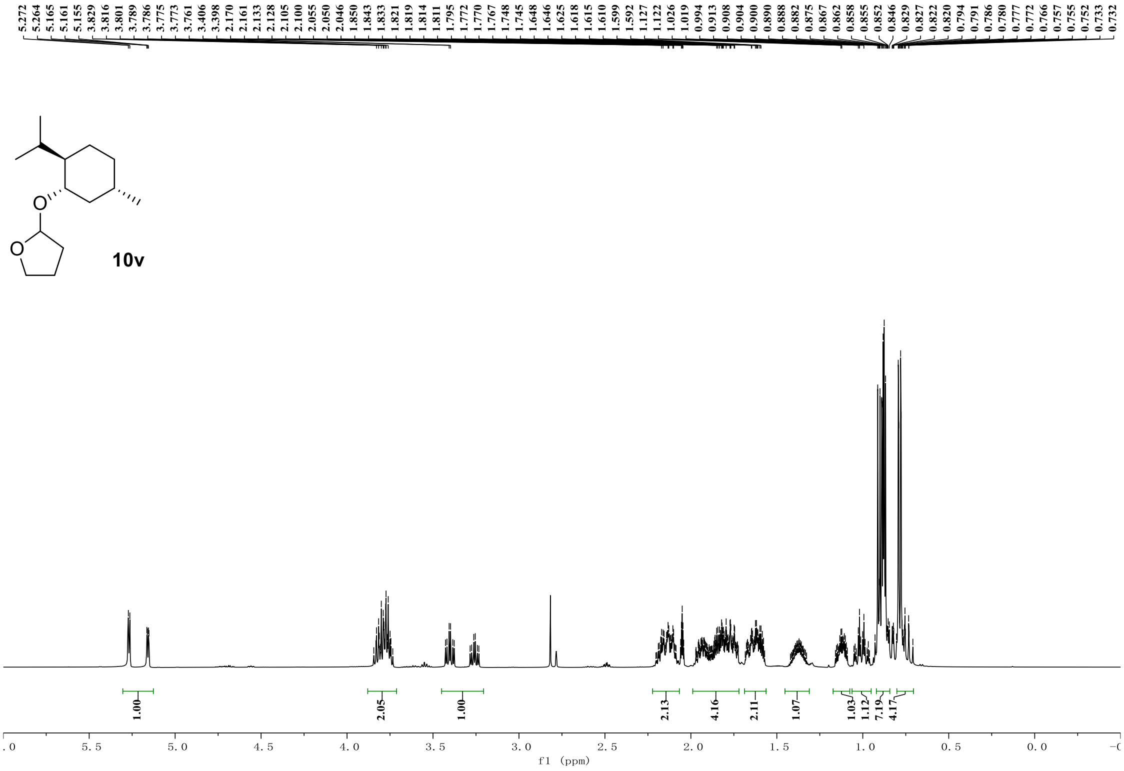

^{1}H NMR was recorded on Bruker 500 MHz; Solvent: Acetone-d_6

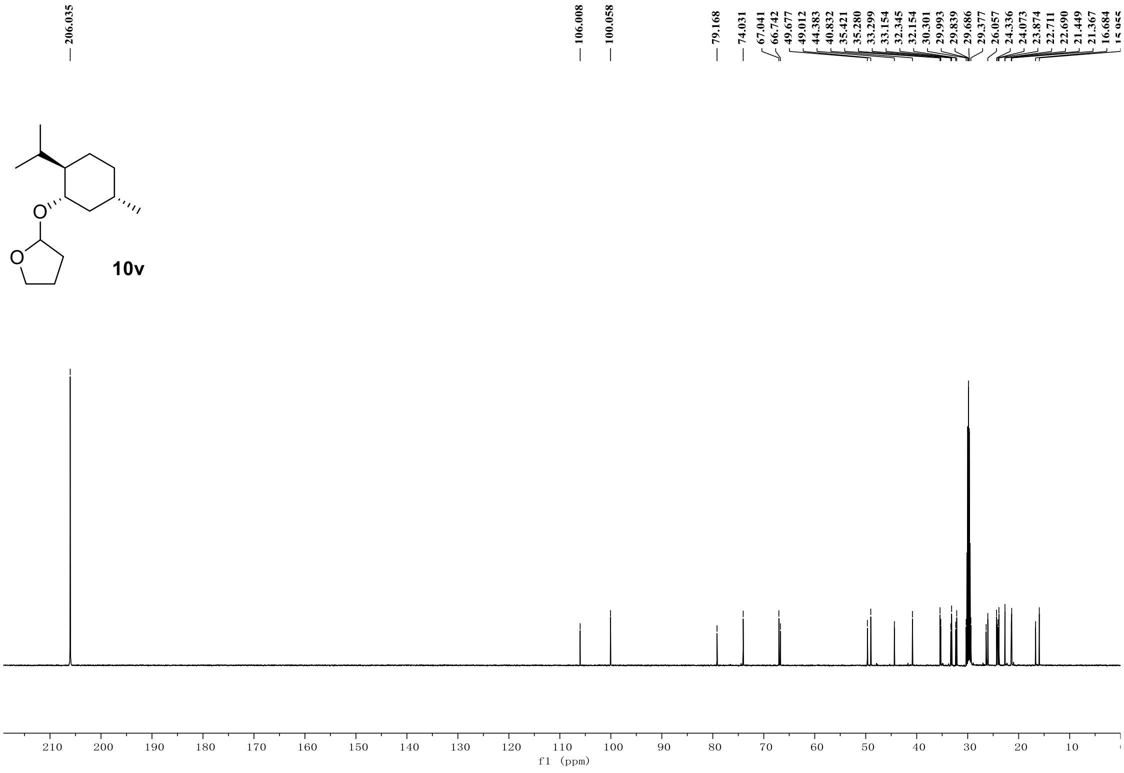

^{13}C NMR was recorded on Bruker 126 MHz; Solvent: Acetone-d_6

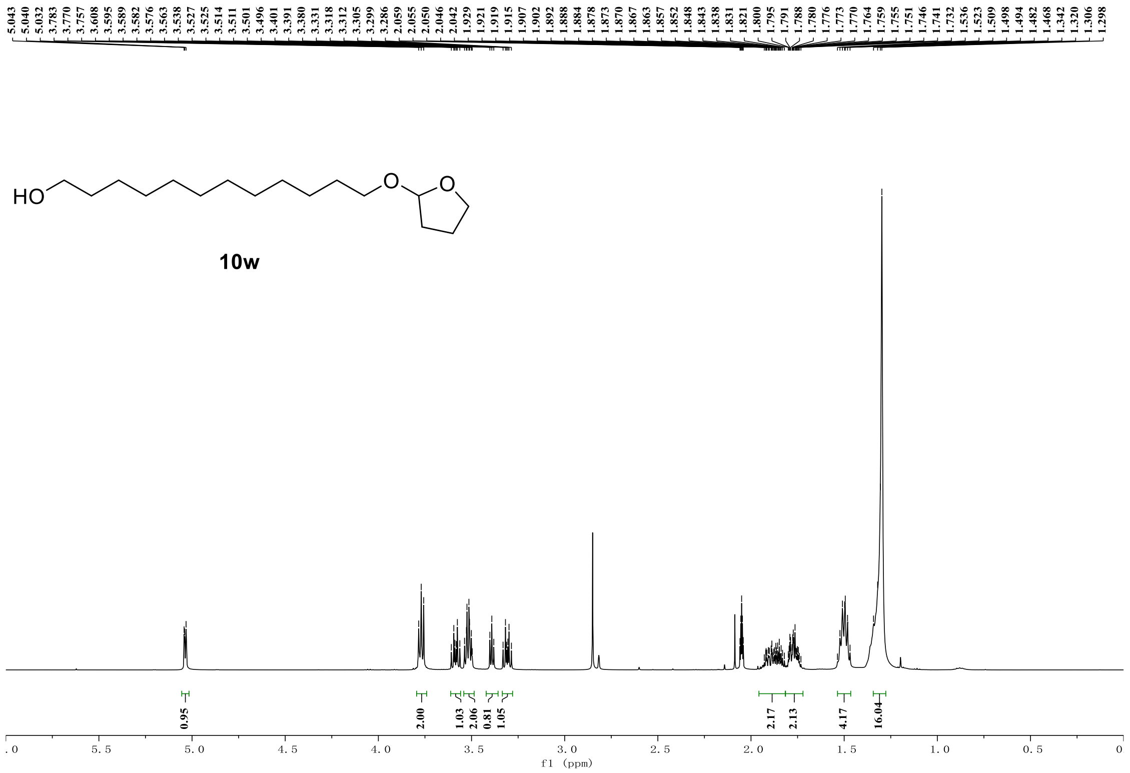

^{1}H NMR was recorded on Bruker 500 MHz; Solvent: Acetone-d_6

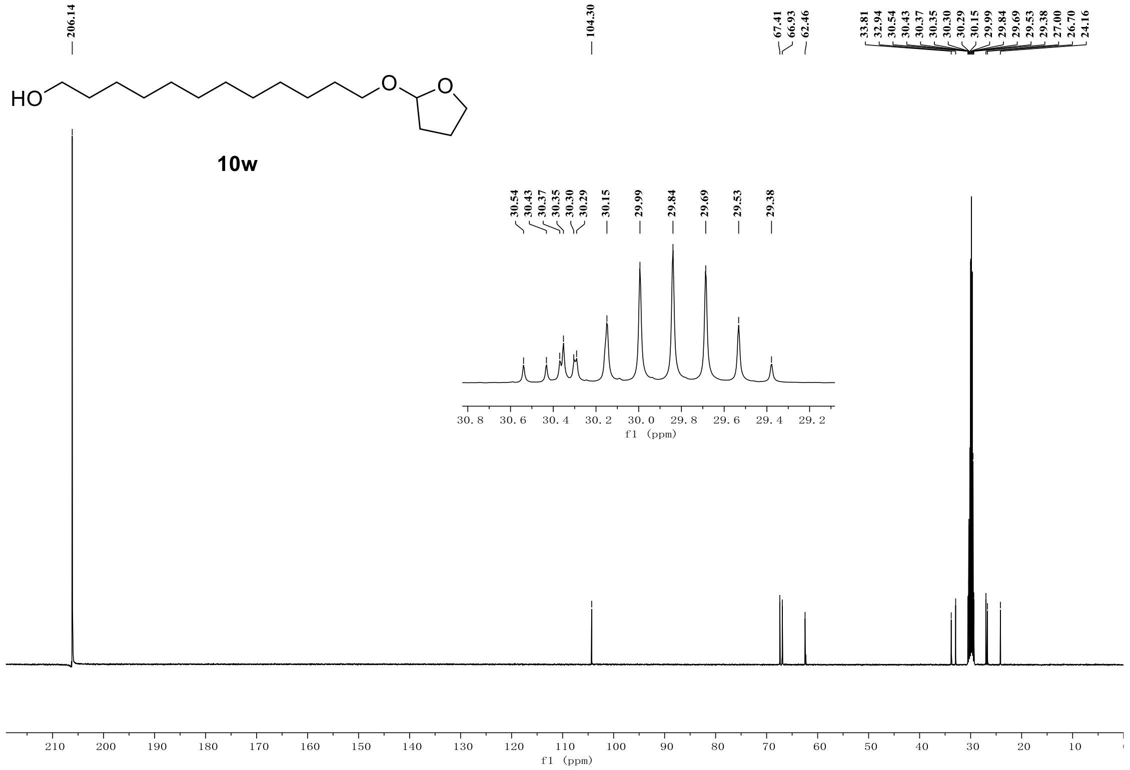

^{13}C NMR was recorded on Bruker 126 MHz; Solvent: Acetone-d_6

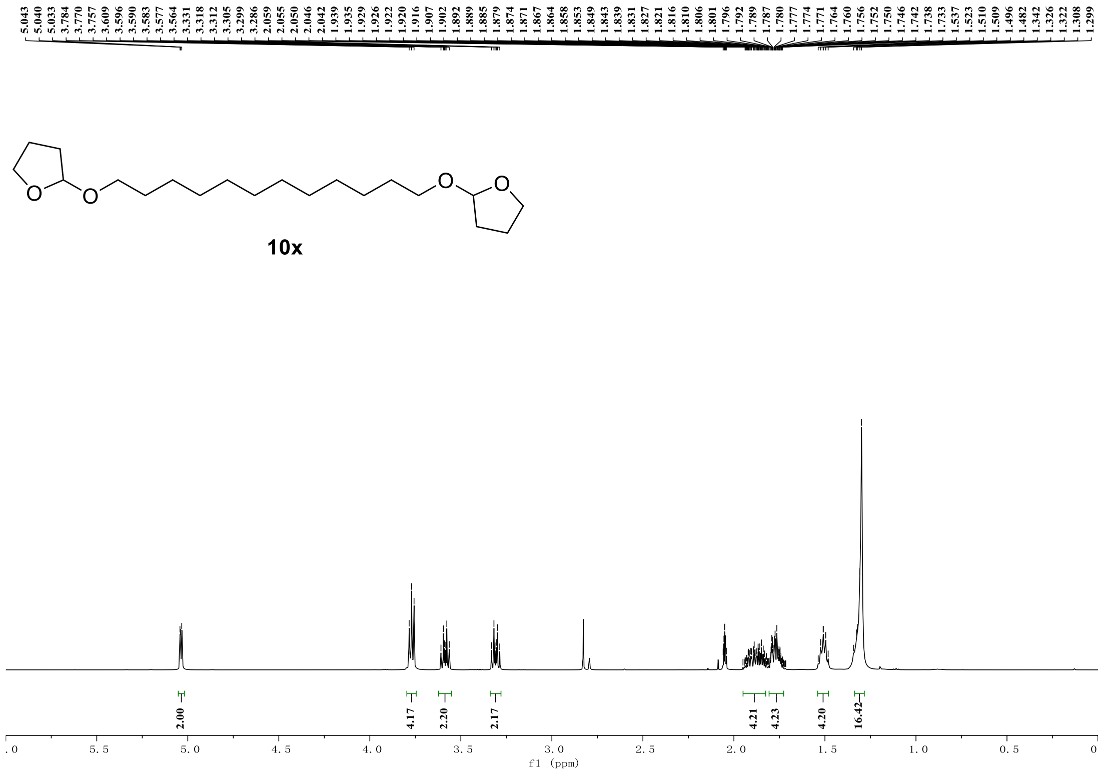

^{1}H NMR was recorded on Bruker 500 MHz; Solvent: Acetone-d_6

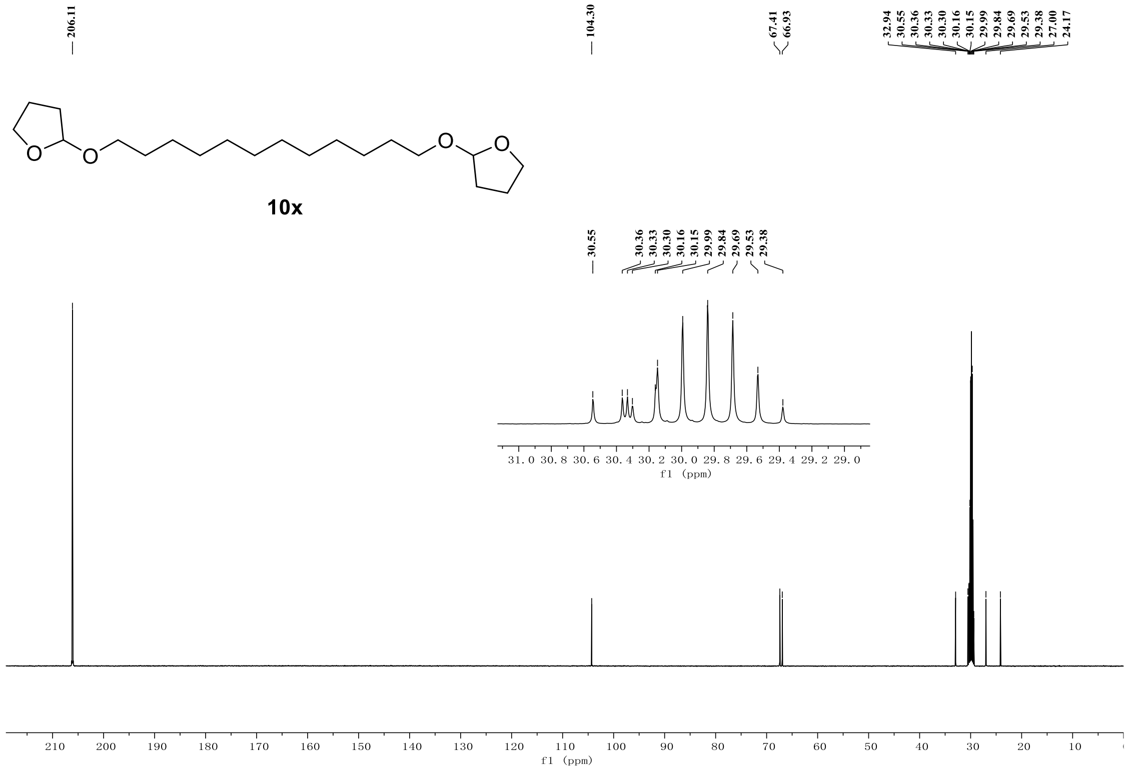

^{13}C NMR was recorded on Bruker 126 MHz; Solvent: Acetone-d_6

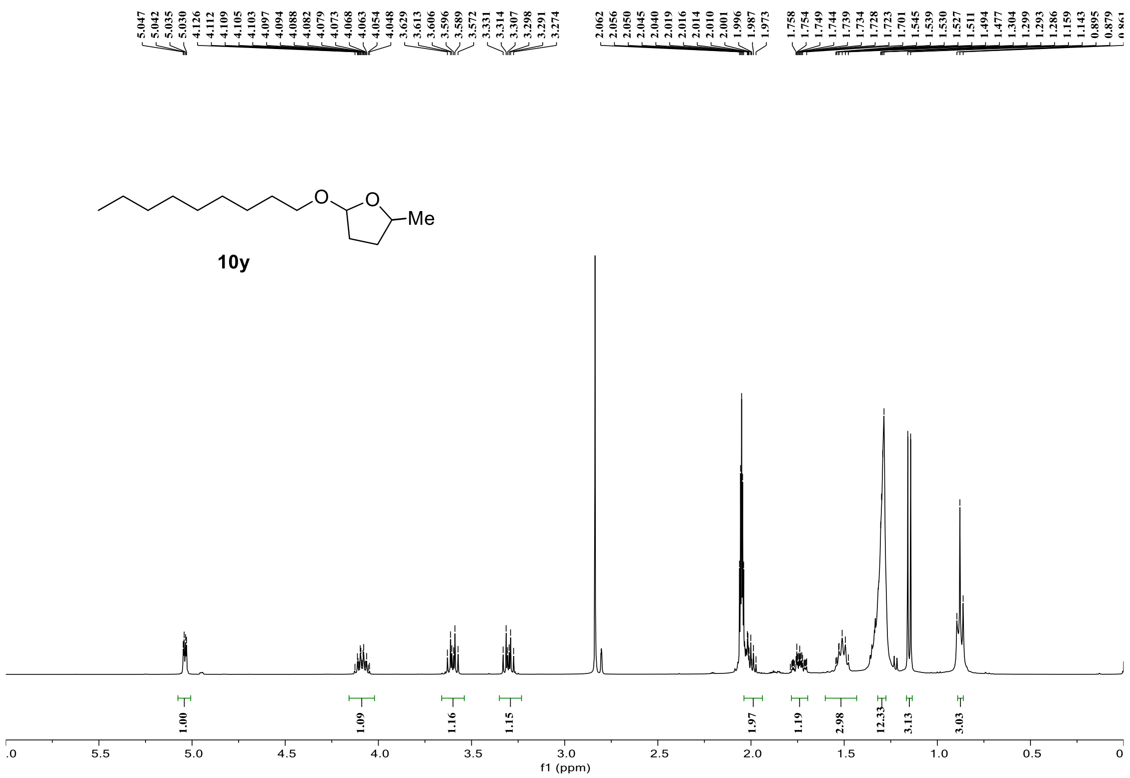

^{1}H NMR was recorded on Bruker 400 MHz; Solvent: Acetone-d_6

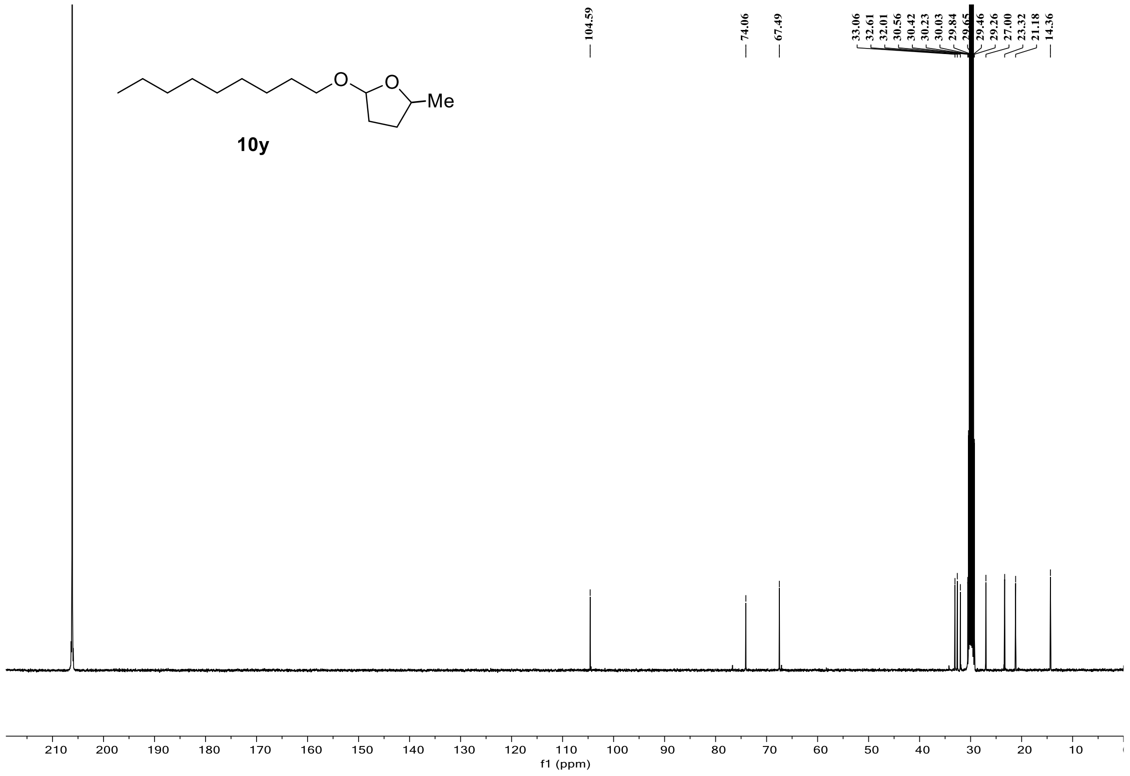

^{13}C NMR was recorded on Bruker 101 MHz; Solvent: Acetone-d_6

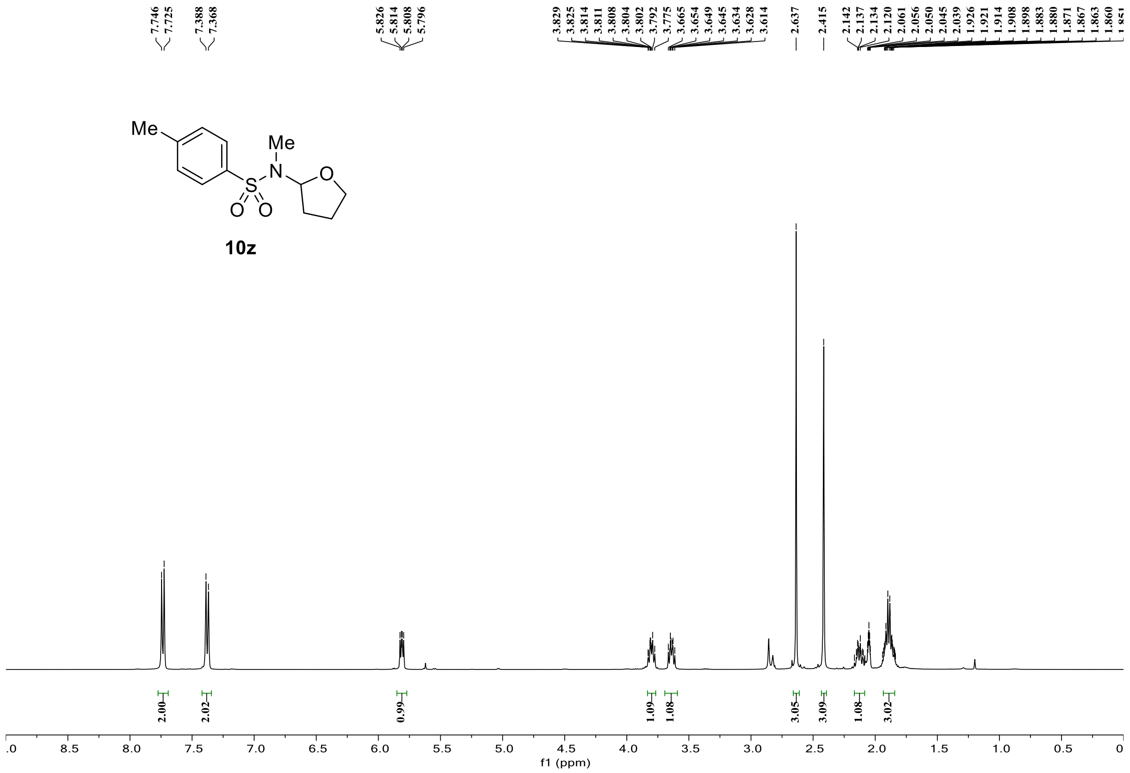

^{1}H NMR was recorded on Bruker 400 MHz; Solvent: Acetone-d_6

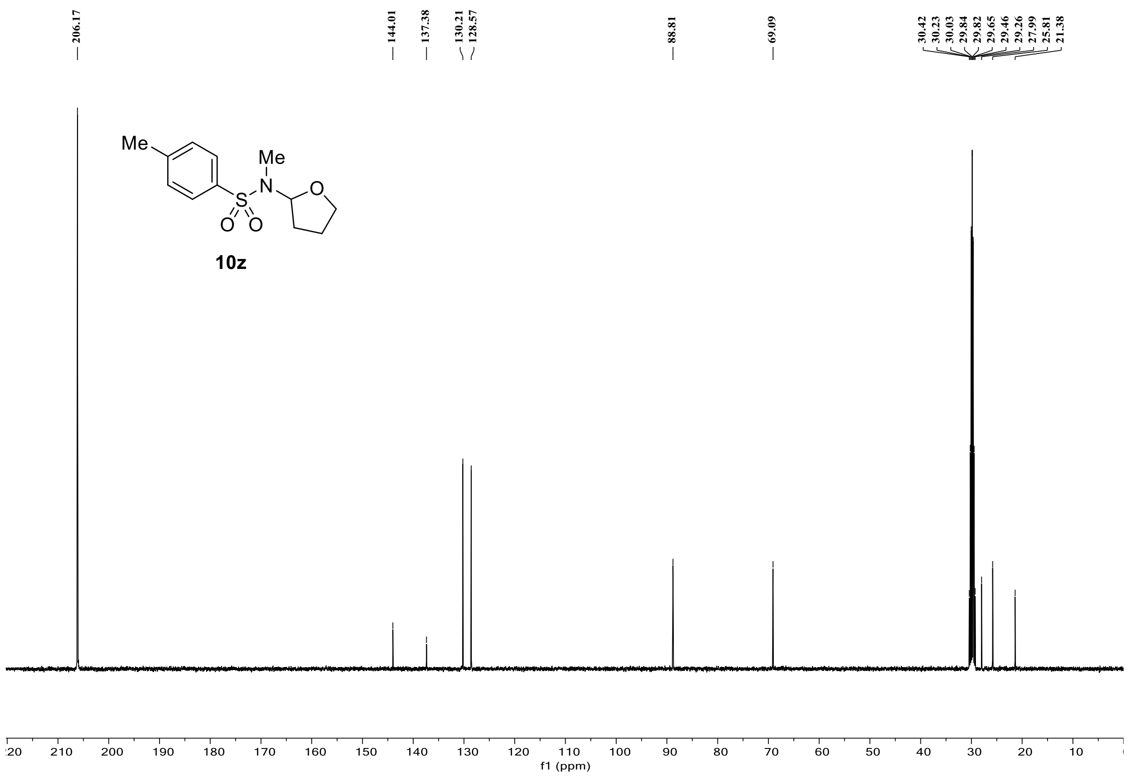

^{13}C NMR was recorded on Bruker 101 MHz; Solvent: Acetone-d_6

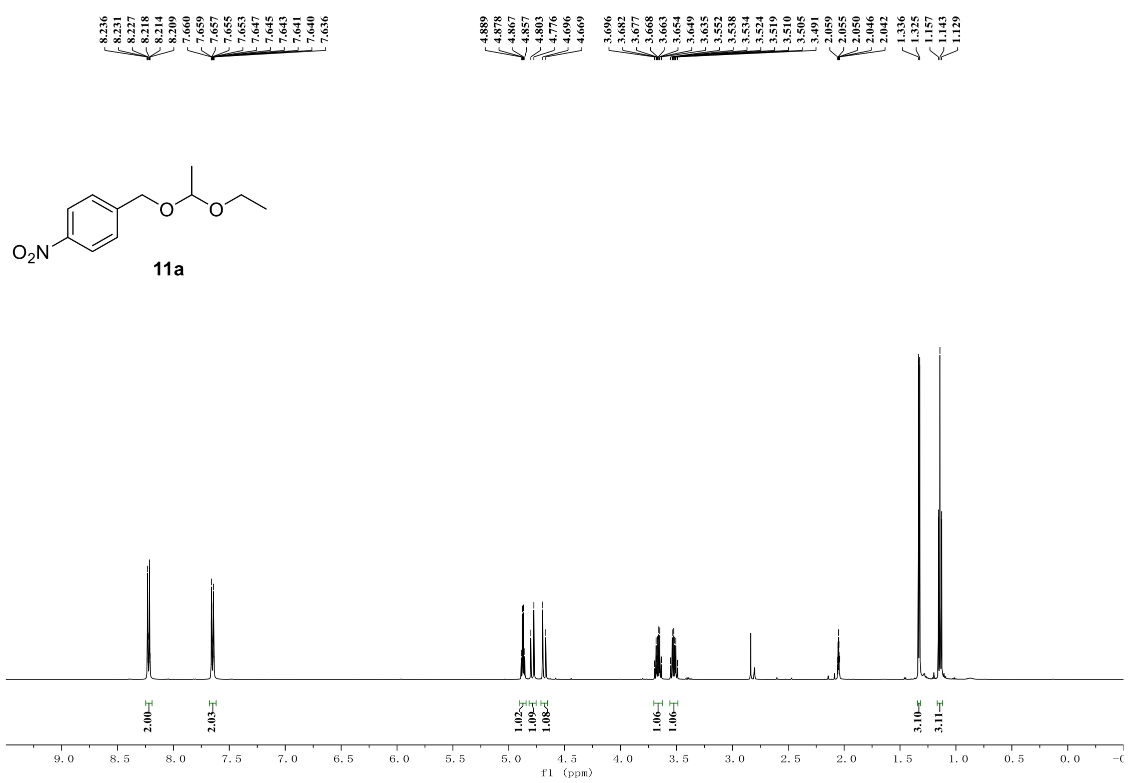

^{1}H NMR was recorded on Bruker 500 MHz; Solvent: Acetone-d_6

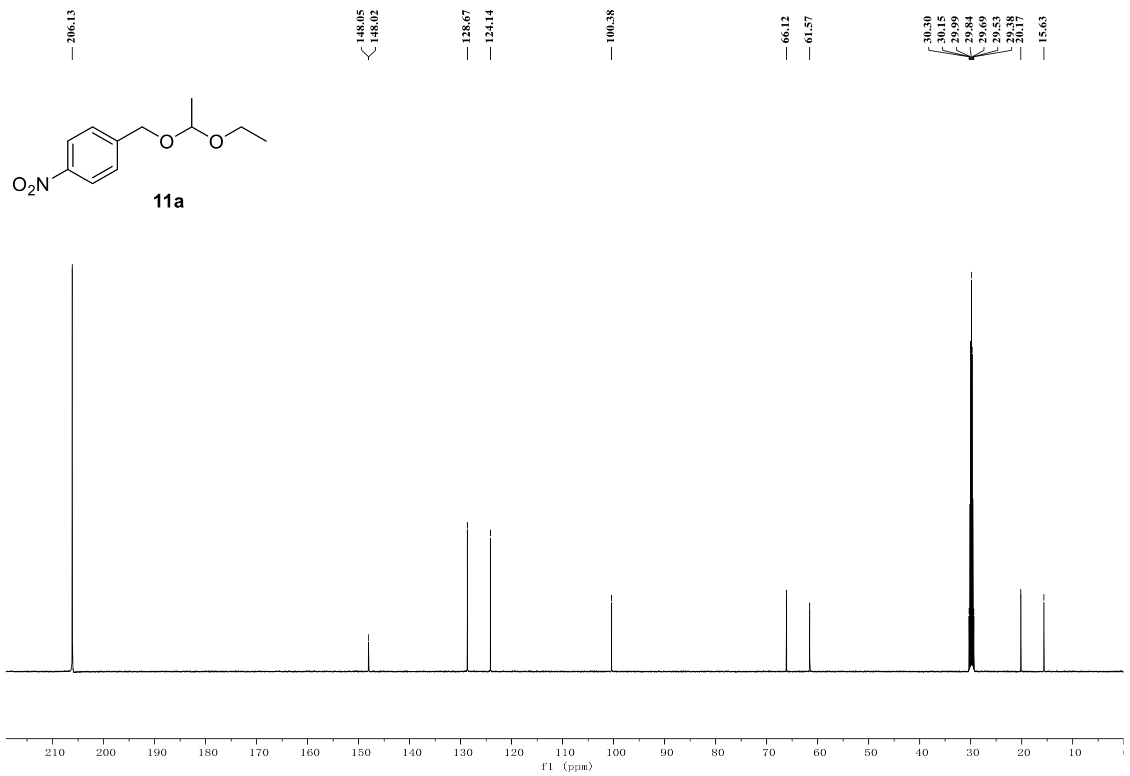

^{13}C NMR was recorded on Bruker 126 MHz; Solvent: Acetone-d_6

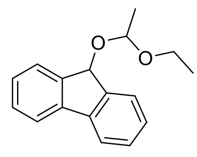

11b

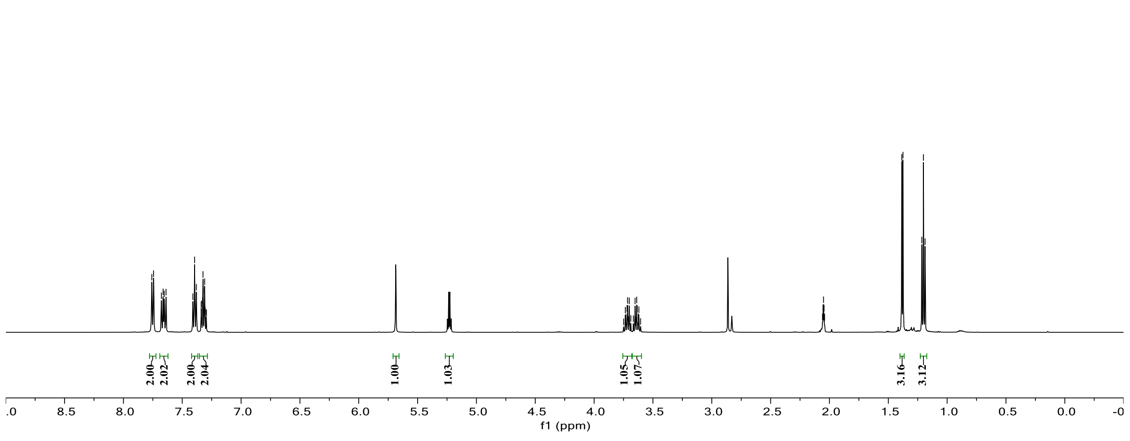

^{1}H NMR was recorded on Bruker 500 MHz; Solvent: Acetone-d_6

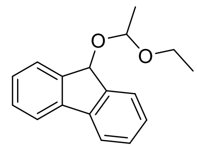

11b

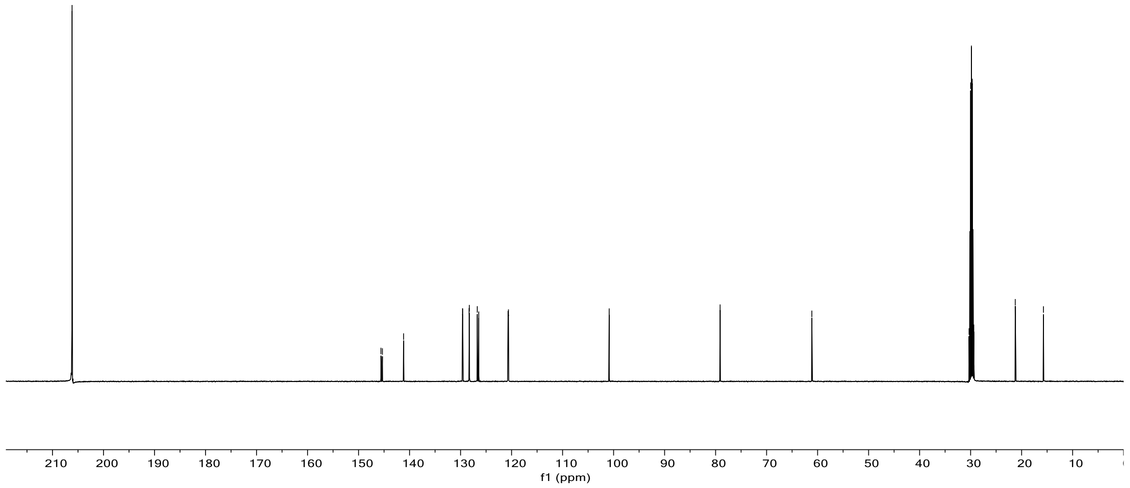

^{13}C NMR was recorded on Bruker 126 MHz; Solvent: Acetone-d_6

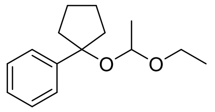

11c

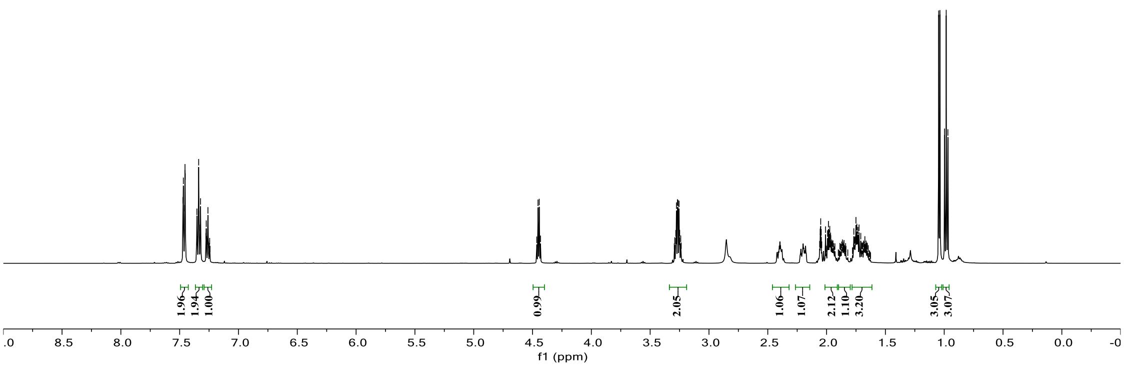

^{1}H NMR was recorded on Bruker 500 MHz; Solvent: Acetone-d_6

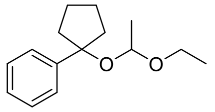

11c

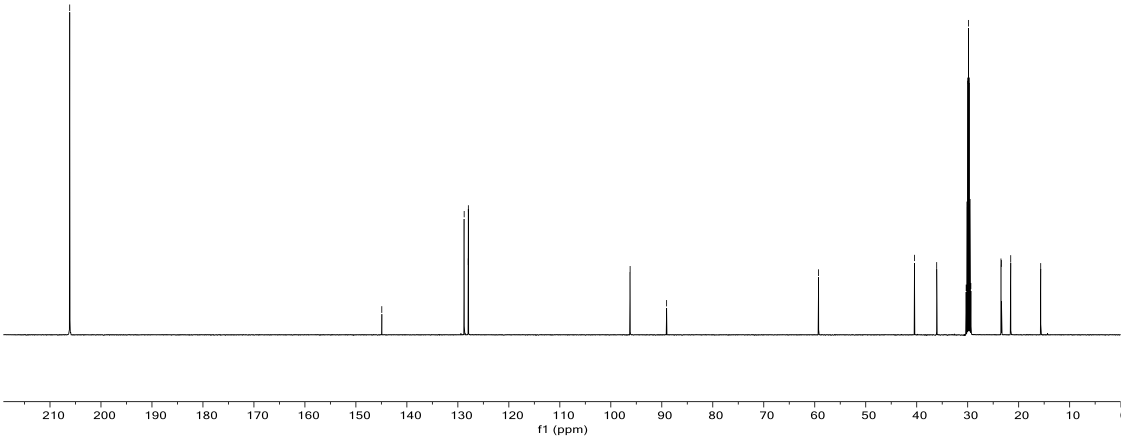

^{13}C NMR was recorded on Bruker 126 MHz; Solvent: Acetone-d_6

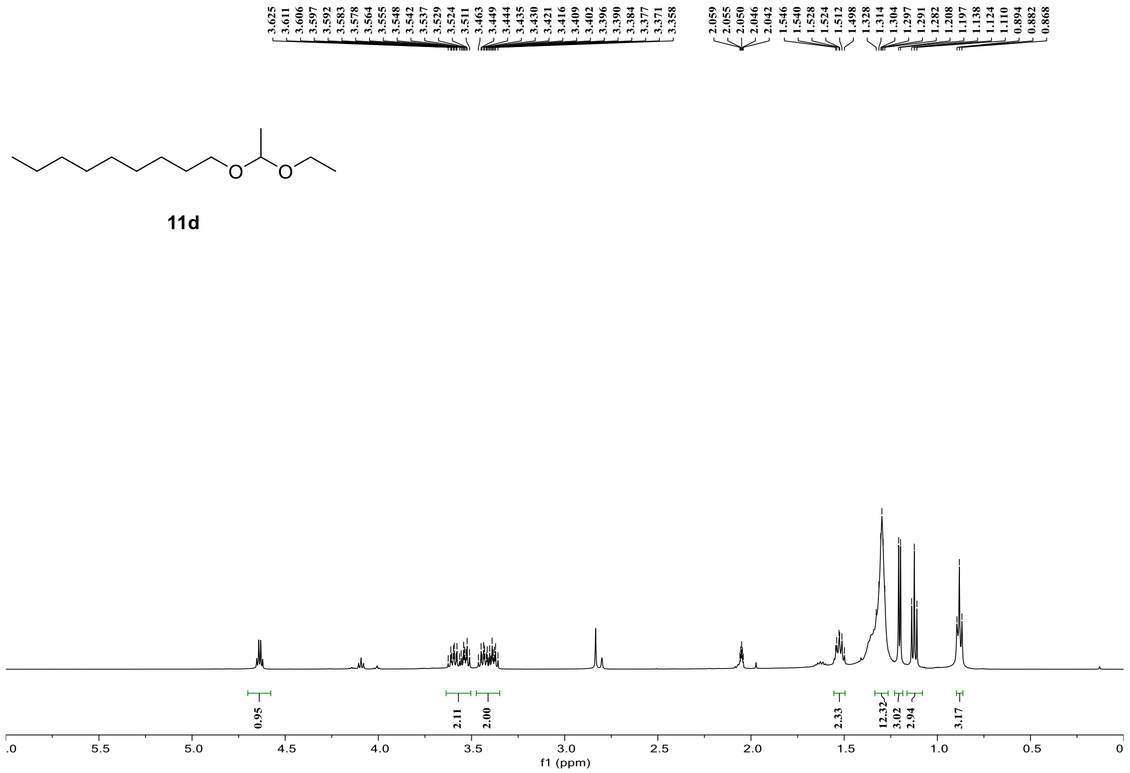

^{1}H NMR was recorded on Bruker 500 MHz; Solvent: Acetone-d_6

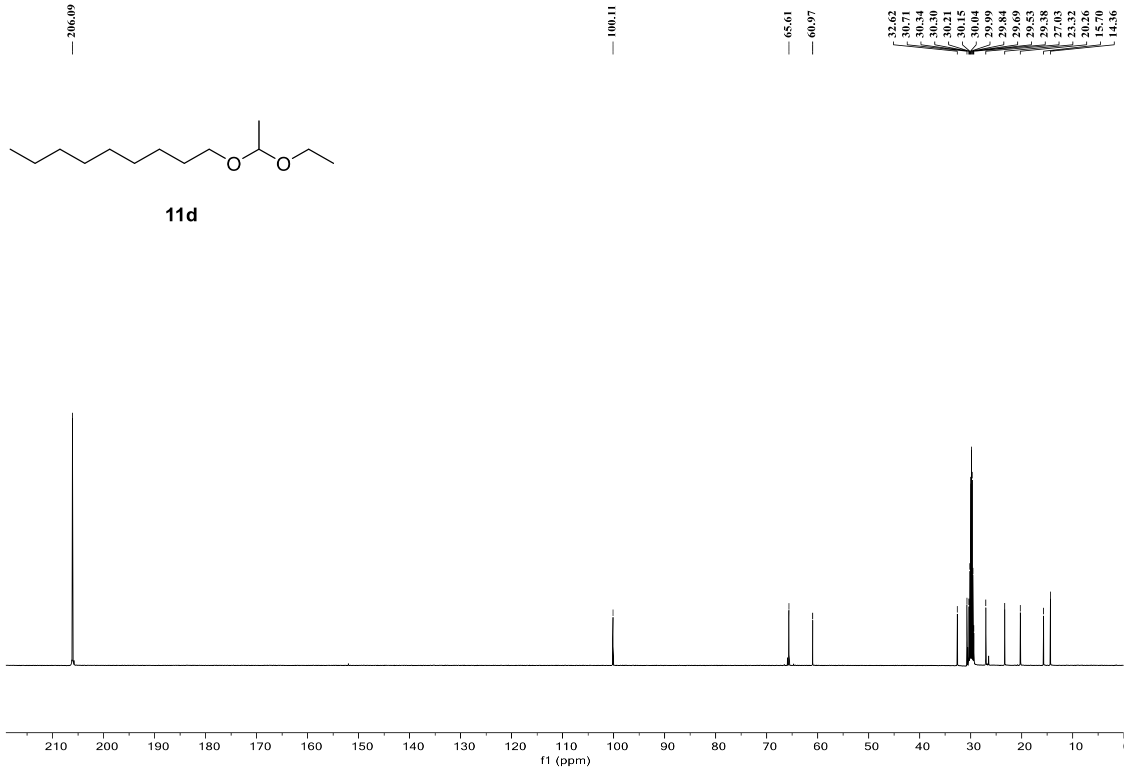

^{13}C NMR was recorded on Bruker 126 MHz; Solvent: Acetone-d_6

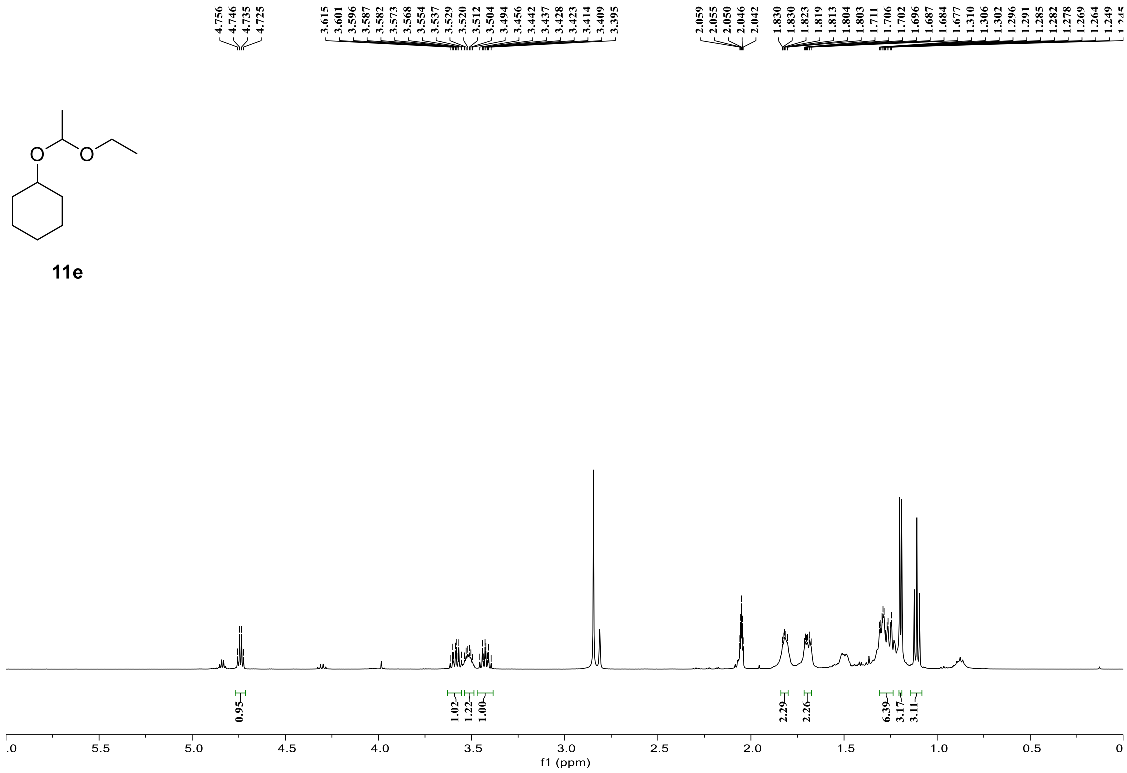

^{1}H NMR was recorded on Bruker 500 MHz; Solvent: Acetone-d_6

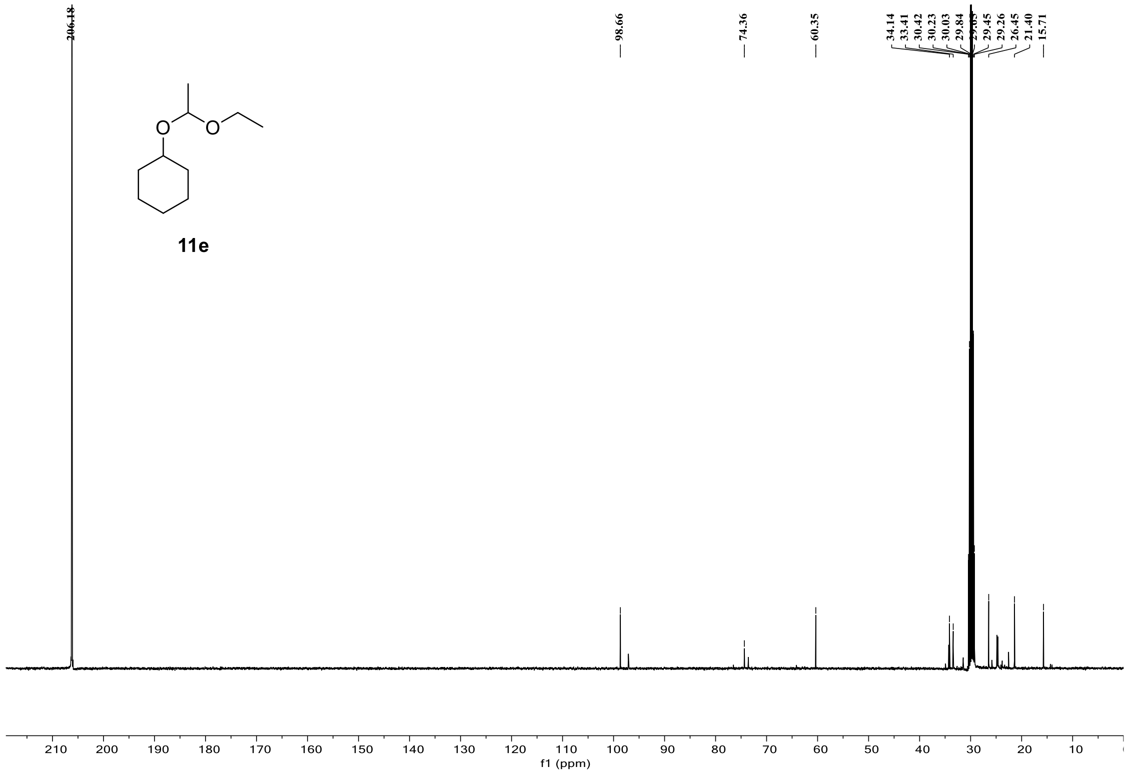

^{13}C NMR was recorded on Bruker 126 MHz; Solvent: Acetone-d_6

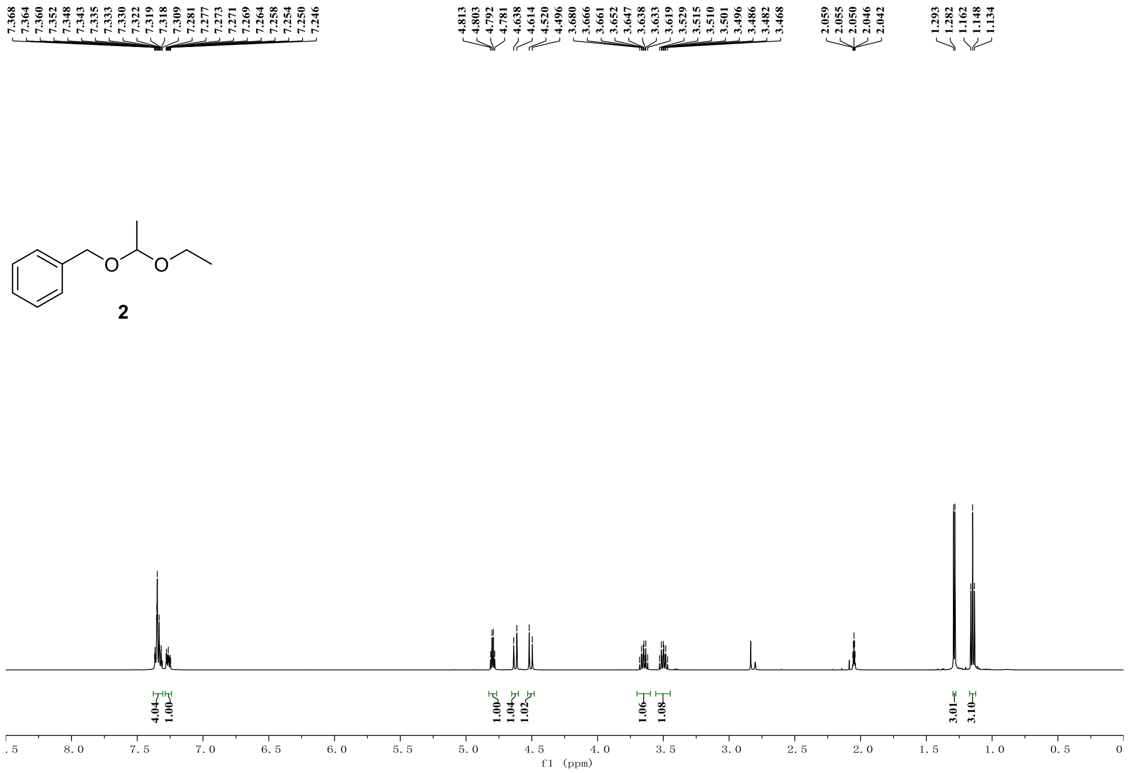

^{1}H NMR was recorded on Bruker 500 MHz; Solvent: Acetone-d_6

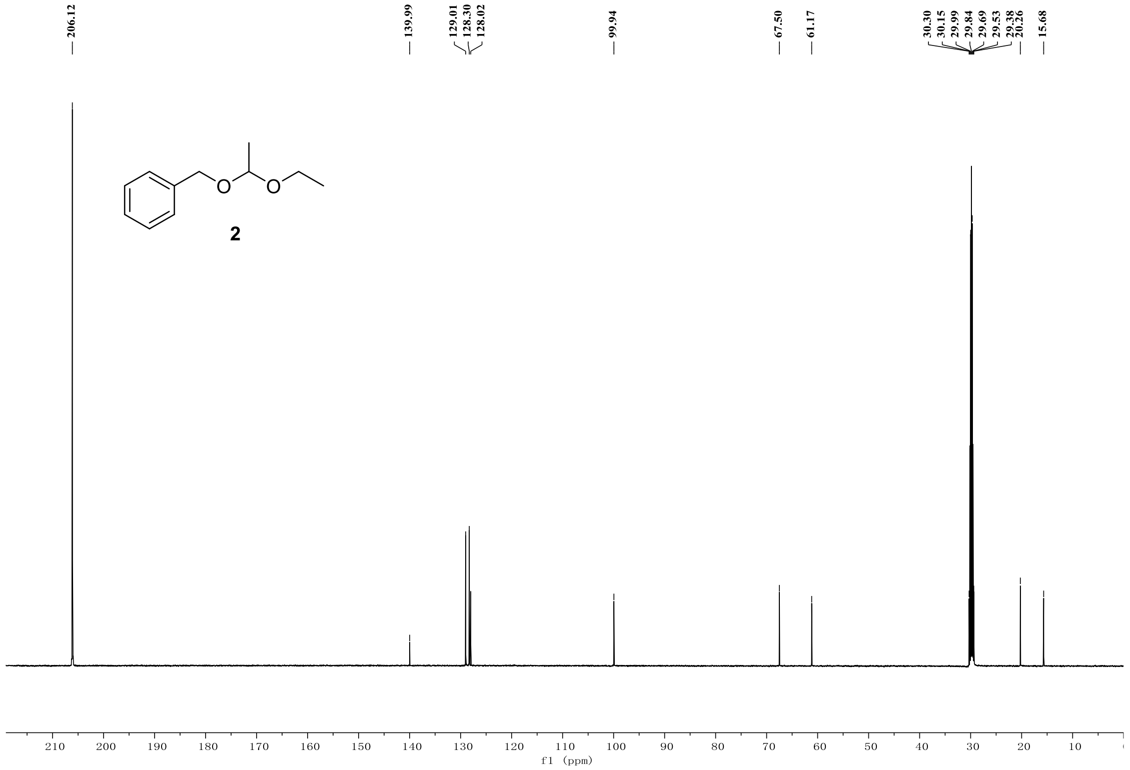

^{13}C NMR was recorded on Bruker 126 MHz; Solvent: Acetone-d_6

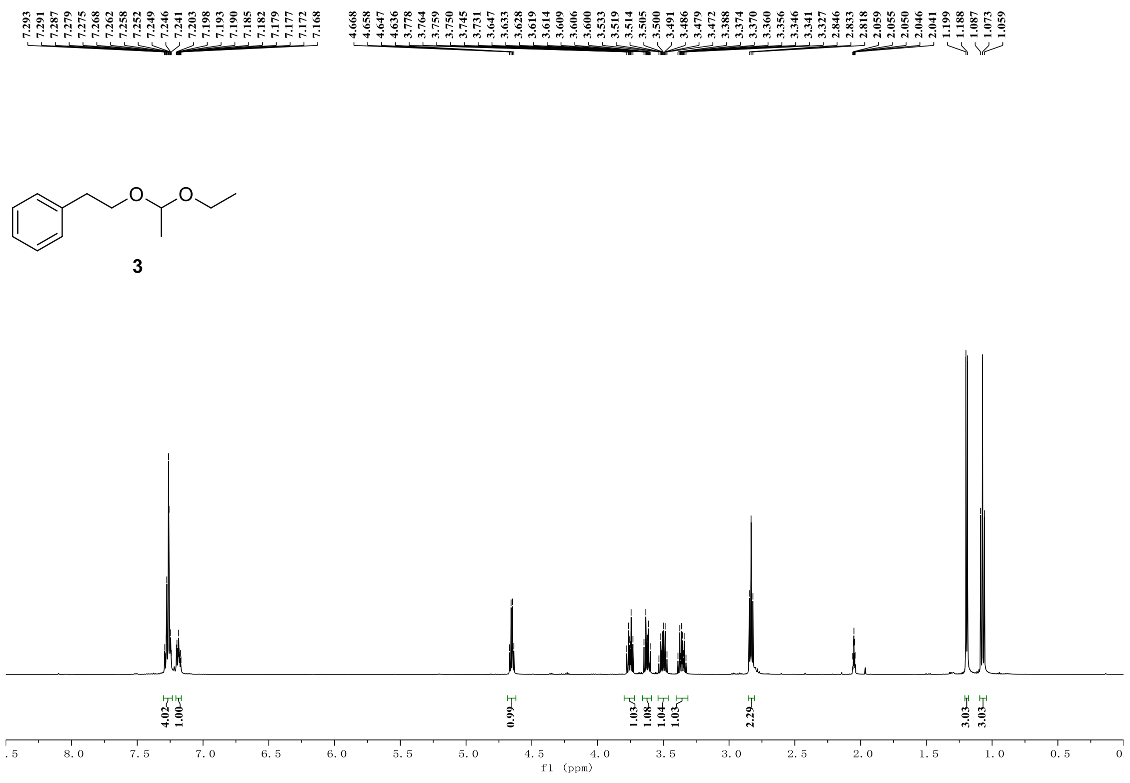

^{1}H NMR was recorded on Bruker 500 MHz; Solvent: Acetone-d_6

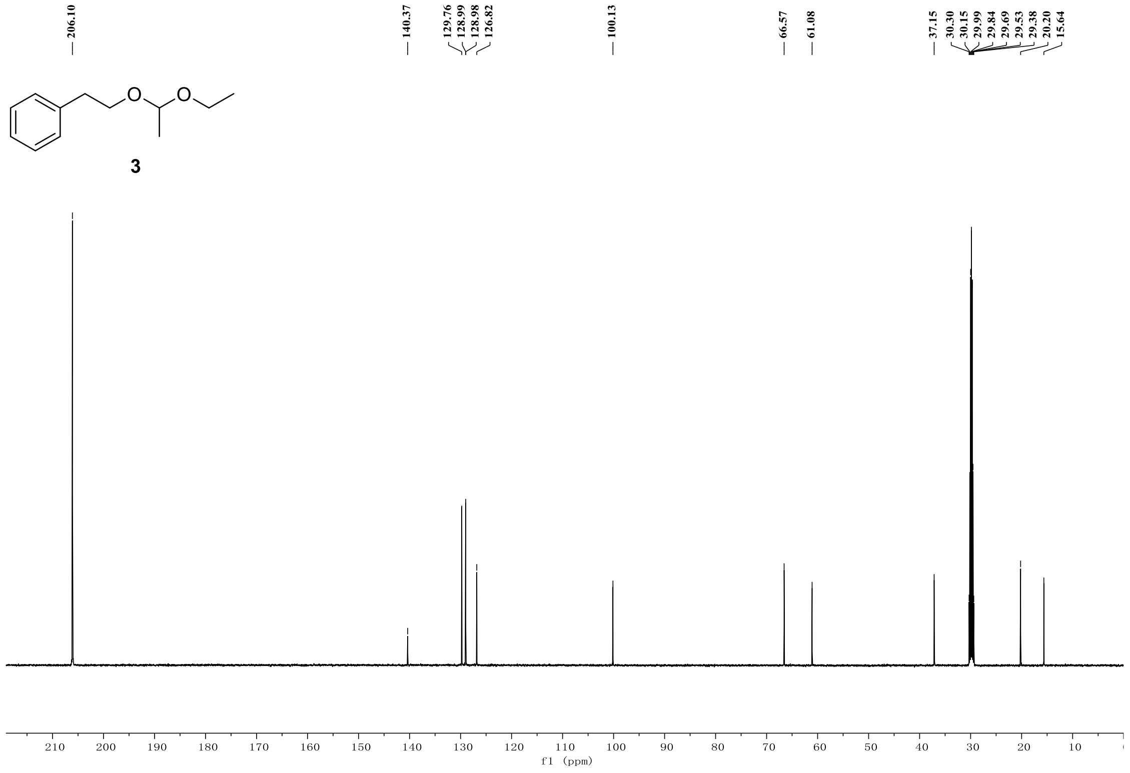

^{13}C NMR was recorded on Bruker 126 MHz; Solvent: Acetone-d_6
